# Site Density Governs Switching Reactivity Regimes During Aerobic C─H Oxidation in Mn Single‐Atom Photocatalysts

**DOI:** 10.1002/anie.3256491

**Published:** 2026-09-24

**Authors:** Viktoria Velichko, Agustín de Arriba, Areti Moutsiou, Elisabetta Inico, Andrea Olivati, Berta Pérez‐Román, Jesús López‐Sánchez, Ik Seon Kwon, Andrea Tonelli, Abhijit Roy, Valentina De Renzi, Raul Arenal, Annamaria Petrozza, Carlo Cavallotti, Gianvito Vilé

**Affiliations:** ^1^ Department of Chemistry, Materials and Chemical Engineering “Giulio Natta” Politecnico di Milano Milano Italy; ^2^ Center for Nanoscience and Technology Istituto Italiano di Tecnologia Milano Italy; ^3^ Electroceramics Department Consejo Superior de Investigaciones Científicas Institute of Ceramics and Glass (ICV‐CSIC) Madrid Spain; ^4^ Department of Energy Science and Engineering Kunsan National University Gunsan‐si Republic of Korea; ^5^ Department of Physical Computer and Mathematical Sciences University of Modena and Reggio Emilia Modena Italy; ^6^ National Research Council (CNR‐NANO) Nanoscience Institute Modena Italy; ^7^ Instituto de Nanociencia y Materiales de Aragón (INMA) CSIC‐University of Zaragoza Zaragoza Spain; ^8^ Laboratorio de Microscopias Avanzadas (LMA) University of Zaragoza Zaragoza Spain; ^9^ Aragonese Agency for Research and Development Foundation (ARAID) Zaragoza Spain

**Keywords:** adaptive catalysts, carbon nitride, catalyst design, heterogeneous catalysis, single‐atom catalysis

## Abstract

Understanding how the density of isolated metal sites affects the structure and function of single‐atom catalysts remains a central challenge, particularly for photocatalysts based on polymeric semiconductors. In this study, we show that Mn incorporation in carbon nitride‐supported single‐atom photocatalysts (Mn_1_@CN*_x_*) triggers a regime transition without detectable Mn aggregation. At low‐to‐intermediate loadings, isolated Mn–N*_x_* sites are accommodated within a partially delaminated CN*_x_* framework, promoting productive carrier trapping, oxygen activation, and selective aerobic benzylic C–H oxidation. Above a critical loading, however, Mn incorporation reorganizes the support into a distorted and densified architecture, modifies the Mn coordination environment, and shifts carrier dynamics toward unproductive recombination. Structural, spectroscopic, and computational analyses connect this transition to framework stacking, electronic disorder, and Mn‐support coupling. During selective aerobic benzylic C–H oxidation, we show that this regime transition translates into a nonmonotonic activity response, in which additional isolated Mn sites become detrimental once support reorganization overrides the benefit of increased site density. Mechanistic experiments and theory support a photoinduced oxygen activation, with the productive regime enabled by the balance between isolated Mn sites and a favorable CN*_x_* trap‐state landscape. These findings establish single‐atom site density as a structural and photophysical design parameter for polymer‐supported photocatalysts.

## Graphical abstract

Increasing the density of isolated manganese (Mn) atoms in carbon nitride (CN_*x*_) photocatalysts does not continuously improve performance. Instead, beyond a critical loading, the polymer support reorganizes, altering charge‐carrier dynamics and oxygen activation while preserving atomic dispersion. The resulting switch from productive to recombination‐dominated behavior defines a new design principle for single‐atom photocatalysts.

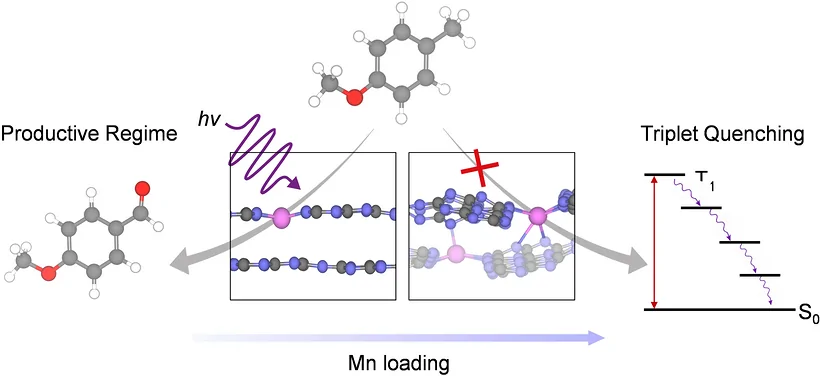

## 1 Introduction

Single‐atom catalysts (SACs) have emerged as a powerful platform for heterogeneous catalysis, offering maximal metal utilization and precise control of local coordination, thereby enabling thermal, electrochemical, and photochemical transformations that are difficult to achieve with conventional supported nanoparticles [1, 2, 3, 4]. While early efforts focused on noble metals, increasing attention has shifted toward earth‐abundant transition metals (i.e., Fe, Co, Ni, Cu, Mn) supported on carbon‐based and polymeric matrices for sustainable catalytic applications. A central unresolved question is how the density of nominally isolated sites influences not only the number of active centers but also the structure of the support that stabilizes them [5, 6, 7]. In fact, although SACs are often discussed in terms of “isolated” versus “aggregated” metal species, recent studies suggest that increasing metal loading can induce subtle but profound changes in the local coordination environment, electronic structure, and even the support architecture, without necessarily forming nanoparticles [8, 9]. These site‐density effects are particularly relevant for polymeric and soft supports, where metal incorporation can drive support reorganization, modify stacking and porosity, and reshape the distribution of electronic trap states [10]. Yet, systematic experimental investigations that correlate incremental metal loading with support reorganization and catalytic behavior remain scarce.

Polymeric semiconductors, and graphitic carbon nitride (CN*_x_*) in particular, represent an ideal platform to interrogate these effects. CN*_x_* combines chemical robustness, visible‐light absorption, and modular synthesis with a structurally flexible, layered framework that can respond sensitively to metal incorporation [11]. However, bulk CN*_x_* typically suffers from rapid charge‐carrier recombination and limited surface area, motivating extensive efforts to tune its structure and electronic properties through doping, exfoliation, and heterostructure formation [12, 13, 14, 15]. Among earth‐abundant metals, manganese (Mn) is particularly attractive due to its rich redox chemistry, multiple accessible oxidation states, and ability to activate molecular oxygen under mild conditions. Mn‐modified CN*_x_* materials have recently shown enhanced performance in photocatalytic reactions, attributed to improved charge separation and oxygen activation [16, 17, 18, 19]. Nevertheless, how incremental Mn loading affects atomic dispersion, coordination geometry, support stacking, and photophysical properties, and how these structural changes translate into photocatalytic function, remains poorly understood.

In this study, we systematically investigate metal site density‐induced regime transitions in Mn single‐atom catalysts supported on carbon nitride (Mn_1_@CN*_x_*). By synthesizing a series of catalysts with precisely controlled Mn loadings, we test the hypothesis that Mn site density acts as a tunable parameter that adaptively reorganizes the carbon nitride framework and thereby changes oxygen activation and photocatalytic performance. The aerobic benzylic C(sp^3^)–H oxidation is used as a model reaction to probe oxygen activation pathways and structure‐function relationships. This transformation is important in organic synthesis and chemical manufacturing, because aldehydes are key intermediates for fine chemicals, fragrances, and pharmaceuticals [20, 21]. Traditionally, the reaction requires harsh conditions such as elevated temperatures, high pressures, and stoichiometric amounts of toxic oxidants (e.g., chromium‐ or selenium‐based reagents) to overcome high bond‐dissociation energies (70‐130 kcal mol^−1^) [22], resulting in poor atom economy, limited selectivity, and significant environmental concerns [23, 24, 25, 26]. In contrast, photocatalytic aerobic oxidation provides a sustainable alternative under mild conditions [27]. We combine structural, spectroscopic, catalytic, and computational analyses to connect Mn site density with atomic dispersion, support organization, charge‐carrier dynamics, and oxygen‐activation pathways.

## 2 Materials and Methods

### 2.1 Catalyst Synthesis

A series of Mn single‐atom catalysts supported on mesoporous graphitic carbon nitride (denoted *y*‐Mn_1_@CN*_x_*, where *y* indicates the Mn loading in wt.%, the subscript “1” indicates the single‐atomic nature of the active Mn centers, and CN*_x_* denotes the mesoporous graphitic carbon nitride support) was synthesized using a hard‐template approach [28]. Briefly, cyanamide was dissolved in an aqueous dispersion of silica nanoparticles (SiO_2_), which served as the hard template to generate mesoporosity in the carbon nitride framework. A defined amount of Mn(II) chloride was then added as the metal precursor to achieve target Mn loadings ranging from 0.1 to 1.9 wt.%. The resulting mixture was evaporated overnight and subsequently calcined at 550°C for 4 h under air to promote polymerization of cyanamide and immobilization of the metal species. After calcination, the silica template was removed by treatment with an aqueous ammonium bifluoride solution (12 g of NH_4_HF_2_ in 50 mL of H_2_O) for 24 h, followed by filtration, washing with ethanol and water, and drying at 80°C overnight. Typically, light‐orange powders (1.4 g) were obtained.

### 2.2 Catalyst Characterization

The chemical composition of the catalysts was investigated by CHNS elemental analysis using a Vario Micro Element Analyzer. The Mn content was quantified by inductively coupled plasma optical emission spectroscopy (ICP‐OES) using an Optima 8000 spectrometer from PerkinElmer. Powder X‐ray diffraction (XRD) patterns were collected on a Bruker D2 Phaser diffractometer equipped with a Cu Kα radiation source (*λ* = 0.15405 nm), operating over a 5‐60° 2*θ* range, with a step size of 0.016° and a counting time of 0.4 s per step. Samples were analyzed on flat PMMA holders under ambient conditions without further treatment. Nitrogen physisorption measurements were conducted using a 3P Sync 400 instrument at −196°C to assess the porous nature of the samples and specific surface areas. Prior to nitrogen adsorption, samples were degassed at 150°C for 24 h to remove any residual moisture or adsorbed contaminants. The Brunauer‐Emmett‐Teller (BET) method was applied to determine the specific surface areas on the adsorption branch of the isotherms within the pressure range of 0.05 < *p/p*_0_ < 0.3. Vibrational properties were investigated by Raman spectroscopy using an InVia Renishaw instrument with a 785 nm excitation wavelength from a diode laser. Measurements were carried out in the spectral range 100‐3500 cm^−1^, averaging 10 scans with 10 s integration per scan. The sharp silicon line located at 520 cm^−1^ was employed as a reference for the Raman shift scale. The samples were focused with an optical microscope equipped with a 50× objective (Leica, Germany). Diffuse reflectance UV‐Vis spectroscopy (DRS) measurements were carried out in the 300‐1000 nm wavelength range using a PerkinElmer Lambda 1050 spectrophotometer equipped with an integrating sphere. Powder samples were sandwiched between a microscopy glass slide and a thin glass cover to ensure a homogeneous optical path. Photoluminescence (PL) spectra were recorded using excitation at 355 nm provided by the third harmonic of a Q‐switched Nd:YVO_4_ laser (Innolas Picolo). The emitted light was collected and guided via an optical fiber to a Maya 2000 Pro spectrometer (Ocean Optics) for spectral analysis. Transient absorption spectroscopy (TAS) measurements were conducted in transmission geometry for both short (≤3 ns) and long (>3 ns) delay times. The white‐light probe beam was generated using the fundamental emission of a femtosecond laser (Light Conversion Pharos) centered at 1030 nm focused on a sapphire crystal, leading to a broadband light source between 500 and 950 nm. The excitation source for short delays was the third harmonic (343 nm) of the femtosecond laser (HIRO module, Light Conversion), whereas for long delays the pump beam (355 nm) was provided by a Q‐switched Nd:YVO_4_ laser (Innolas Picolo), electronically synchronized with the femtosecond source. Measurements were carried out on 5 mg mL^−1^ suspensions of carbon nitride‐based materials in acetone. X‐ray photoelectron spectroscopy (XPS) measurements were performed in a custom‐designed ultra‐high vacuum (UHV) system operating at the base pressure of 2 × 10^−10^ mbar. XPS core‐level spectra were collected using a nonmonochromatic X‐ray source (Mg Kα 1253.6 eV) and an Omicron EA‐125 hemispherical analyzer. Samples were prepared by dispersing approximately 5‐10 mg of powder in 2 mL of ethanol, followed by sonication. The resulting suspension was drop‐cast onto a clean SiO_2_ substrate, previously annealed in UHV at 500°C to remove surface contaminants. After solvent evaporation, a thin film was obtained, and the sample was transferred into the UHV chamber and allowed to outgas overnight. As the Mn content is below one atomic percent, a relatively thick film was deposited to achieve a measurable Mn 2p signal, thick enough to saturate the film contribution. For C 1s, N 1s, and Mn 2p detail spectra, the analyzer pass energy and the energy step were set to 30 and 0.1 eV, respectively. Charging effects due to the low conductivity of the sample were corrected by referencing all binding energy (BE) values to the main component of the N 1s peak in CN*_x_* at 398.7 eV, attributed to the bridging nitrogen atoms in the triazine rings (C═N–C). XPS spectra were fitted using Igor Pro with Voigt profiles, after Shirley background subtraction. High‐angle annular dark field (HAADF) scanning transmission electron microscopy (STEM) imaging and STEM energy‐dispersive X‐ray spectroscopy (EDS) analyses were carried out using a ThermoFisher Scientific Titan Low‐Base probe‐corrected microscope. For visualization purposes, selected HAADF‐STEM micrographs were processed by contrast enhancement to improve the visibility of isolated bright features associated with Mn single atoms. The studies were conducted at 300 kV. The X‐ray absorption spectroscopy (XAS) measurements at the Mn K‐edge were performed at the 8C NanoProbe XAFS beamline of the Pohang Light Source‐II (PLS‐II), using an undulator as the incident X‐ray source. A cryogenically cooled Si(111) double‐crystal monochromator was used for energy selection, and approximately 30% detuning was applied at the Mn K‐edge to suppress higher‐order harmonics. The incident beam was defined by slits upstream of the sample chamber, yielding a beam size of approximately 1.0 mm (horizontal) × 0.5 mm (vertical). All XAS spectra were recorded in transmission mode using gas ionization chambers filled with a mixture of Ar and N_2_ gases. A standard Mn foil was simultaneously measured and used for energy calibration. After data acquisition, normalization, and Fourier transform of the EXAFS region were performed using the Demeter software package (version 0.9.26).

### 2.3 Catalytic Tests

Photocatalytic oxidation reactions were carried out in a PhotoCube reactor (ThalesNano Inc.), equipped with interchangeable light‐emitting diodes (LEDs). Unless otherwise stated, all experiments were performed using the 395 nm LED. In a typical experiment, the benzylic substrate (10 µL, 0.08 mmol), photocatalyst (5 mg), and solvent (1 mL) were added to a 4 mL quartz vial, which was sealed with a screw‐cap septum. The sealed vial was purged with O_2_ for 1 min by inserting an oxygen‐filled balloon needle through the septum while venting through a second needle. After purging, the vial was sealed and irradiated under stirring (800 rpm) at a fixed distance of 1 cm from the LED source. After each reaction, the reaction suspension was filtered through a PTFE syringe filter (0.45 µm) to remove catalyst particles prior to analysis. Conversion and product yields were quantified by high‐performance liquid chromatography (HPLC) using an Agilent 1200 system equipped with a UV‐Vis detector set at 210 nm. A 50 µL aliquot of the filtrate was diluted in 650 µL of acetonitrile before injection. Samples and standards (5 µL) were injected directly into a 250 mm × 4.6 mm Hypersil GOLD aQ 5 µm 175 Å column (Thermo Scientific). The mobile phase was comprised of a 60:40 (v:v) acetonitrile/water mixture, with a total flow rate of 0.7 mL min^−1^ at 40°C. Product identification and quantification were carried out after calibration with commercial standards of *p*‐methoxytoluene, *p*‐methoxybenzaldehyde, and *p*‐anisic acid. For recycling studies, after reaction completion, the catalyst was separated by filtration, washed with water (15 mL) and ethanol (15 mL), dried in an oven at 60°C, and then used as the catalyst for the next cycle. Mechanistic studies were carried out with the addition of 1 equiv. (0.08 mmol) of the corresponding scavenger (benzoquinone, furfuryl alcohol, isopropanol, or ascorbic acid) prior to O_2_ purging.

### 2.4 Computational Details

Structural investigations were carried out using density functional theory (DFT) calculations implemented in the Vienna *ab initio* Simulation Package (VASP) [29, 30, 31]. The exchange‐correlation functional was described using the Perdew‐Burke‐Ernzerhof (PBE)+*U* approach [32, 33], with the Hubbard *U* parameter taken from the literature [34, 35]. The use of periodic boundary conditions in VASP minimizes artifacts associated with nonperiodic cluster models, while the Hubbard *U* correction improves the description of localized electronic states without the computational expense of hybrid functionals during geometry optimization. Dispersion interactions were accounted for using Grimme's D3 correction scheme [36]. Valence electrons were described using plane waves with a kinetic energy cutoff of 400 eV, whereas core electrons were represented by projector augmented wave (PAW) pseudopotentials [37, 38]. Brillouin‐zone sampling was chosen to ensure convergence of the calculated properties [39]. The simulation cell parameters were *a* = 15.59 Å, *b* = 20.70 Å, and *c* = 30.00 Å.

The reactivity was investigated using DFT with atom‐centered Gaussian basis sets, as implemented in Gaussian16 [40], since this theory is better suited for describing the kinetics of molecular systems in different spin states. Energies, structures, and Hessians were calculated using the PBE0 hybrid functional, corrected for dispersion with Grimme's D3(BJ) model, and the def2‐TZVP basis set [41], as this has shown to provide good agreement with high‐level CCSD(T) results [42]. Solvation effects in acetone were described using the SMD implicit continuum model [43]. Thermodynamic properties, including entropies and Gibbs free energies, were evaluated at 298 K and 1 bar within the rigid‐rotor harmonic‐oscillator (RRHO) approximation. EStokTP [44] was used to automate the calculations.

## 3 Results and Discussion

### 3.1 Controlled Synthesis and Persistence of Mn Single‐Atom Sites

The physicochemical properties of the catalysts were assessed using a set of complementary characterization techniques covering both bulk and surface properties. Elemental analyses (Table 1) of the Mn single‐atom catalysts revealed C/N ratios of ca. 0.60 for all samples, consistently below the theoretical mass C/N for C_3_N_4_ is of 0.643. This indicates a partial content of structural defects (e.g., terminal N–H and O‐containing functionalities) [13, 14, 19]. ICP‐OES analyses confirmed the effective incorporation of Mn, yielding metal loadings between 0.10 and 1.90 wt.%. The linear correlation between Mn content and the amount of precursor added during the synthesis demonstrates reliable control over metal loading within the carbon nitride framework (see Figure S1).

**TABLE 1:** Elemental composition and textural properties of CN*_x_* and Mn_1_@CN*_x_*.

Catalyst	C^a^ (wt.%)	N^a^ (wt.%)	H^a^ (wt.%)	C/N (‐)	Mn^b^(wt.%)	*S*_BET_ ^c^(m^2^ g^−1^)
CN*_x_*	30.4	52.6	1.8	0.58	—	185
0.10‐Mn_1_@CN*_x_*	33.0	56.0	2.6	0.59	0.10	256
0.35‐Mn_1_@CN*_x_*	33.2	54.2	2.8	0.61	0.35	250
0.65‐Mn_1_@CN*_x_*	32.6	53.3	2.7	0.61	0.65	163
1.00‐Mn_1_@CN*_x_*	32.8	53.4	2.7	0.61	1.00	219
1.50‐Mn_1_@CN*_x_*	32.6	52.5	2.6	0.62	1.50	221
1.90‐Mn_1_@CN*_x_*	30.1	50.2	2.9	0.60	1.90	39

^a^ CHNS.

^b^ ICP‐OES.

^c^ N_2_ isotherm collected at 77 K.

The morphology and microstructure of representative Mn_1_@CN*_x_* catalysts were examined by aberration‐corrected HAADF‐STEM (Figure 1; Figures S2 and S3). EDS elemental mapping revealed a homogeneous distribution of Mn, C, and N throughout the framework (Figure 1a), while the characteristic porous, pseudo‐crystalline texture of the carbon nitride, composed of interconnected nanosheets typical of mesoporous CN*_x_* materials, was also observed (Figure 1b). Such morphology is consistent with previous reports on mesoporous and graphitic carbon nitride frameworks [45, 46]. High‐resolution HAADF‐STEM micrographs display isolated bright spots attributed to atomically dispersed Mn atoms (Figure 1c,d; Figures S2 and S3), with no detectable evidence of Mn nanoparticles or clusters, even at the highest Mn loading (1.90 wt.%), in good agreement with earlier studies on single‐atom catalysts [47, 48].

**FIGURE 1:**
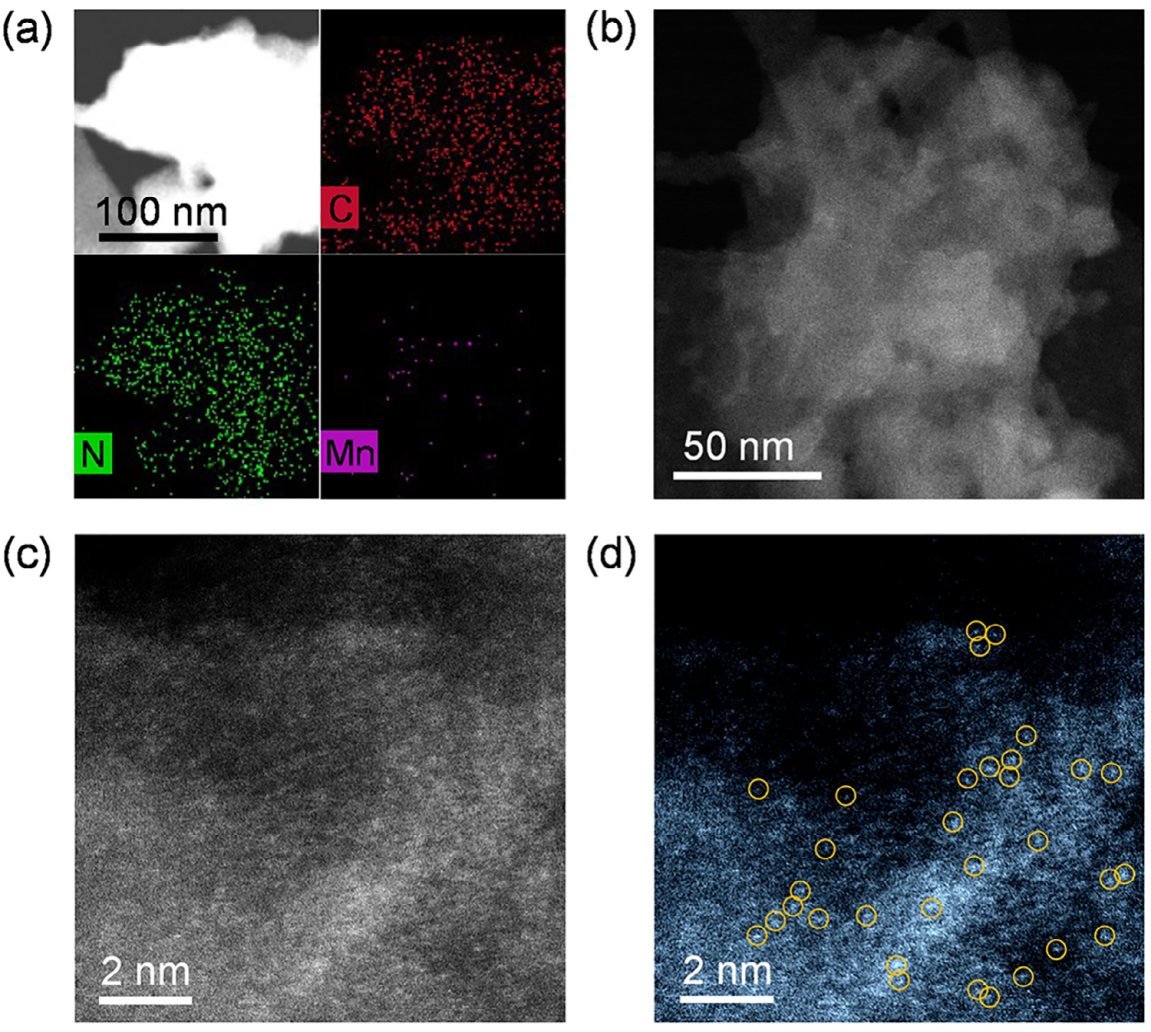
HAADF‐STEM image and corresponding EDS elemental maps (C, N, Mn) of 1.90‐Mn_1_@CN*_x_* (a). Low‐magnification HAADF‐STEM image of 1.90‐Mn_1_@CN_*x*_ highlighting the porous nanosheet morphology (b). High‐resolution HAADF‐STEM image (c) with selected single atoms marked with yellow circles (d) of 1.90‐Mn_1_@CN*_x_*.

To verify whether Mn remains atomically dispersed across the loading series, synchrotron‐based X‐ray absorption spectroscopy (XAS) was performed at the Mn K‐edge (Figure 2). Figure 2a shows the X‐ray Absorption Near Edge Structure (XANES) spectra for three Mn_1_@CN*_x_* samples (1.00‐Mn_1_@CN*_x_*, 1.50‐Mn_1_@CN*_x_*, and 1.90‐Mn_1_@CN*_x_*) together with Mn foil and MnO_2_ as references. For all Mn_1_@CN*_x_* samples, the main absorption edge, which corresponds to the 1s to 4p transition, rises sharply near 6545 eV and sits between those of Mn foil and MnO_2_. The k‐space Extended X‐ray Absorption Fine Structure (EXAFS) spectra of all Mn_1_@CN*_x_* samples (Figure 2c) differ markedly from those of Mn foil and MnO_2_, exhibiting oscillations predominantly at low k values (0 to 8 Å^−1^) characteristic of scattering from light atoms, whereas Mn foil and MnO_2_ have signals above 10 Å^−1^. This demonstrates that the Mn samples coordinate primarily with light atoms involving nitrogen or oxygen, while Mn foil and MnO_2_ feature Mn–Mn bonds that give rise to their characteristic high‐k signals [49]. Consistently, the Fourier‐transformed EXAFS spectra in R‐space (Figure 2d) display a dominant first‐shell contribution assigned to Mn–N/O coordination in the Mn_1_@CN*_x_* samples, whereas no higher‐shell Mn–Mn scattering features are observed, indicating that Mn predominantly exists as isolated single atoms. This agrees well with our HAADF‐STEM images. The absence of Mn–Mn coordination is further confirmed by WT‐EXAFS analysis (Figure 2e), which shows a clear intensity maximum around 1.5 Å in R‐space and between 4 and 6 Å^−1^ in k‐space. This signature is typical of scattering by light atoms and closely resembles the Mn–O feature in MnO_2_, suggesting that Mn coordinates chiefly with nitrogen or oxygen in the CN*_x_* lattice. Notably, the WT‐EXAFS map for Mn_1_@CN*_x_* shows no trace of the Mn–Mn envelope observed in the Mn foil, thereby confirming the single‐atom nature of our materials. These observations agree with the HAADF‐STEM results and demonstrate that Mn remains atomically dispersed even at the highest loading investigated (1.90 wt.%), although sub‐nanometric aggregation cannot be completely excluded within the detection limits of the present techniques.

**FIGURE 2:**
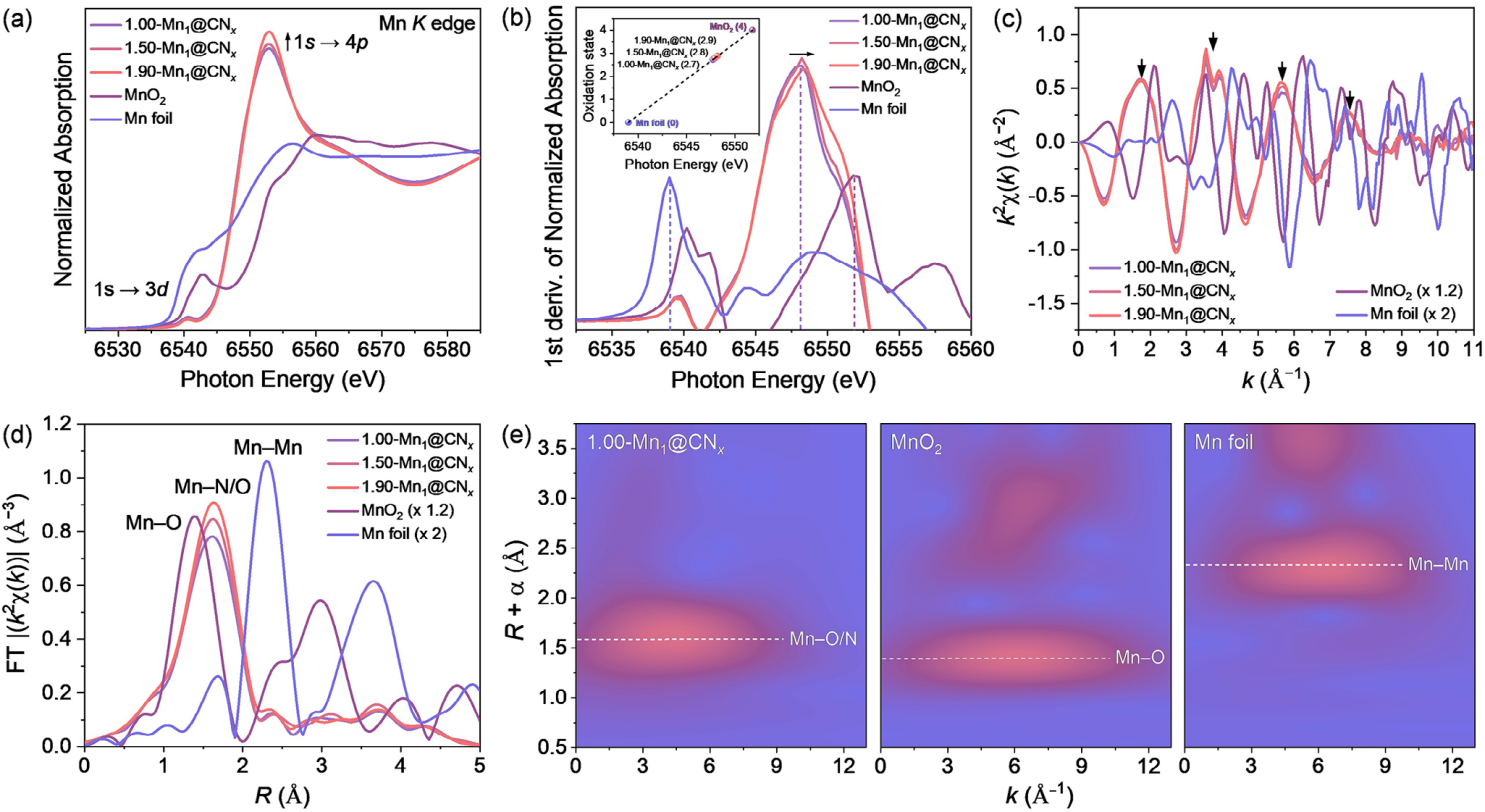
XAS data for Mn_1_@CN*_x_* samples as a function of Mn content. (a) Mn K‐edge XANES data, (b) first derivative plot of XANES, (c) k^2^‐weighted EXAFS data with reference samples (MnO_2_, Mn foil), (d) Fourier‐Transformed (FT)‐EXAFS data, and (e) WT‐EXAFS data shown in both k‐space and R‐space.

### 3.2 Mn Site Density‐Induced Structural Reorganization

Having established that Mn remains atomically dispersed throughout the loading series, we next investigated whether increasing site density alters the local coordination environment of the isolated Mn centers and the structure of the CN*_x_* host. XRD patterns (Figure S4) exhibit two well‐defined reflections at 2*θ* = 12.75° and 27.50°, corresponding to the (100) and (002) planes of carbon nitride, respectively. The main diffraction peak at 27.50° is associated with interlayer stacking of conjugated aromatic units, whereas the peak at 12.75° originates from in‐plane periodicity of the tri‐*s*‐triazine motifs (i.e., trigonal N linkage of the tri‐*s*‐triazine units) [45, 46]. While all catalysts display these two reflections, their relative intensities evolve systematically with increasing Mn loading. In particular, a progressive broadening of the (002) reflection is observed, and the effect becomes more pronounced above 1.00 wt.% Mn, indicating a gradual loss of long‐range stacking order. To evaluate the surface area and porosity of the catalysts, N_2_ physisorption studies at –196°C were conducted (Table 1). While moderate Mn loadings preserve mesoporosity (160‐250 m^2^ g^−1^), higher loadings progressively reduce accessible surface area, culminating in a sharp drop for 1.90‐Mn_1_@CN*_x_* (39 m^2^ g^−1^), consistent with cumulative framework densification and partial pore collapse.

These findings are supported by Raman spectroscopy, which provides insight into the local bonding environment and layer organization as a function of Mn loading. The spectra are consistent with those reported for CN*_x_*‐based single‐atom systems [50], featuring a broad fluorescence background between 1050 and 1600 cm^−1^, associated with interlayer interactions within the carbon nitride framework (Figure 3a). The pristine CN*_x_* support exhibits two main Raman bands centered at around 712 and 1238 cm^−1^, assigned to breathing modes of the triazine rings [50] and C(sp^2^)–N bending vibrations within the extended conjugated triazine framework [50], respectively. Compared to bulk CN*_x_*, where these bands typically appear at slightly lower frequencies (705 and 1233 cm^−1^) [50], the observed blue shift toward higher frequencies suggests phonon confinement and quantum size effects arising from thinner carbon nitride nanolayers [51]. Upon Mn incorporation (Figure 3a), the Raman response evolves systematically. An overall increase in intensity and gradual blue shift of the main features is observed, indicating modifications to the local bonding environment. At higher Mn loadings (>1.00 wt.%), additional bands emerge at *ca*. 470 and 750 cm^−1^, associated with distortions of the triazine framework [51], while a new Raman feature at approximately 985 cm^−1^ appears, increasing in intensity with the Mn content. This band is attributed to vibrational modes involving Mn–N or Mn–O–N coordination, as also reported for Ho‐doped CN*_x_* systems [52]. Its evolution is consistent with local structural rearrangements around the Mn centers and the progressive loss of long‐range order inferred from XRD (Figure S4), while the absence of additional diffraction peaks suggests that no new crystalline phases are formed. To quantify these structural changes, we analyzed the intensity ratio between the Raman modes at approximately 750 (*I*_750_) and at 712 cm^−1^ (*I*_712_), which has been proposed as a descriptor of layer stacking in CN*_x_* systems [51]. As shown in Figure 3b, this ratio was extracted by Voigt function fitting of the Raman spectra. The evolution of the *I*_750_/*I*_712_ ratio reveals a nonmonotonic structural response to Mn incorporation. At low‐to‐moderate loadings (0.10‐1.00 wt.%), the increase in *I*_750_/*I*_712_ up to ∼1 suggests enhanced layer delamination or reduced interlayer coupling, consistent with a more monolayer‐like organization [51]. Beyond this regime, the subsequent decrease in the ratio (∼0.6 at higher loadings) indicates re‐stacking or structural densification, likely driven by cumulative framework distortion and increased cross‐linking induced by Mn coordination. This behavior highlights that Mn does not simply disrupt stacking but actively reshapes the balance between exfoliation and layer re‐aggregation (Figure 3a).

**FIGURE 3:**
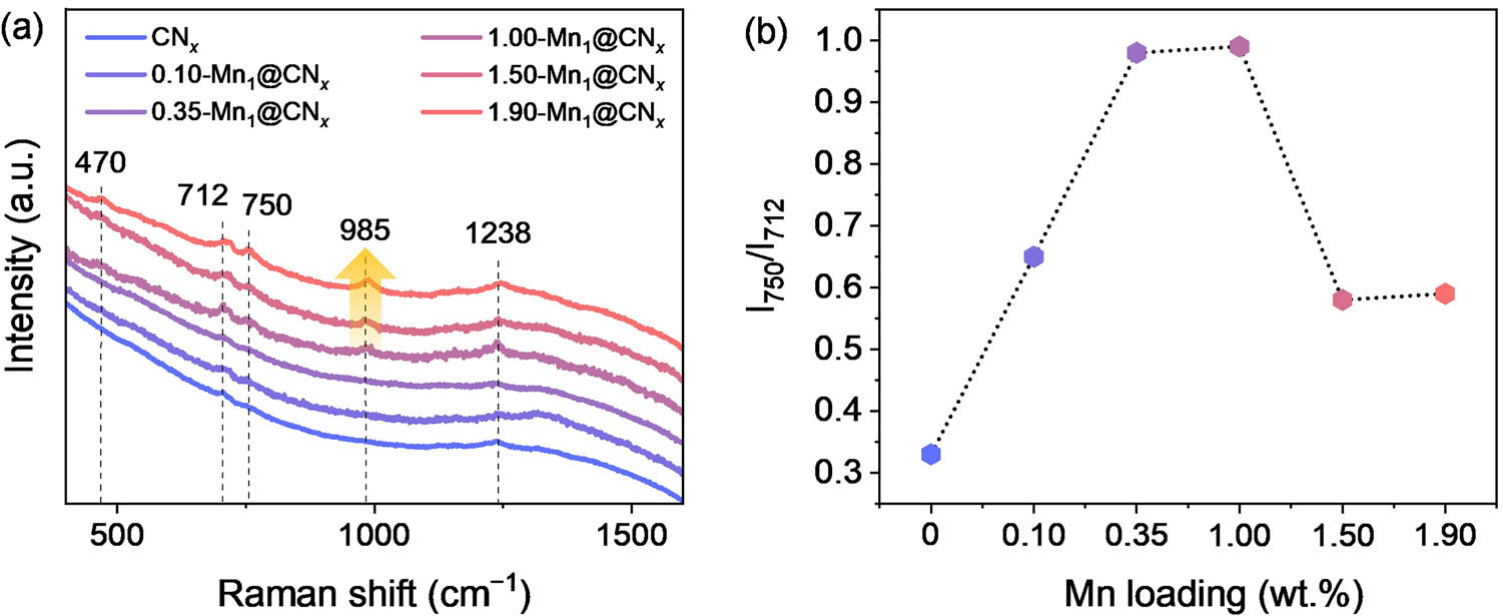
Raman spectra (a) and I_750_/I_712_ Raman ratio (b) for selected Mn_1_@CN*_x_* catalysts.

The structural evolution inferred from XRD, Raman spectroscopy, and nitrogen physisorption suggests that increasing Mn site density progressively reorganizes the CN*_x_* framework. To further evaluate this structural interpretation, we revisited the XAS data and performed a detailed analysis of the XANES and EXAFS regions. The XANES spectra exhibit a systematic increase in white‐line intensity with Mn loading (Figure 2a), with an edge position that places the average Mn oxidation state between metallic Mn and Mn(IV) oxide [53], indicating a higher density of unoccupied states. This trend is accompanied by a gradual shift of the first‐derivative maximum toward higher energies (Figure 2b). Based on these reference compounds, this shift corresponds to an estimated increase in the average Mn oxidation state from approximately +2.7 to +2.9. However, given the small energy differences involved, these values should be regarded as indicative of a modest evolution toward a more oxidized Mn environment rather than absolute formal oxidation states. Together, these observations suggest that increasing site density progressively modifies the electronic structure of the isolated Mn centers [54]. Quantitative EXAFS fitting (Table S1 and Figure S5) shows excellent agreement with the experimental spectra (R‐factor = 0.001‐0.002) and further reveals that the local coordination environment evolves with loading. While the first coordination shell remains dominated by a Mn–N coordination number close to 4, two different Mn–N bond lengths are required to reproduce the experimental spectra at high loadings, indicating that the local geometry is somewhat distorted within the CN*_x_* cavity. We additionally detected Mn–C interatomic distances at 2.5 to 2.8 Å, which correspond to second‐ or third‐shell neighbors. The coordination number for the Mn–C interaction also rises from about 3.6 to 4.2 as the Mn content increases, in accordance with the tendency of the CN*_x_* framework to adopt more distorted structures at higher Mn loadings. These changes point to stronger coupling between the isolated Mn centers and the surrounding carbon nitride framework as the site density increases. The evolution of the local Mn coordination environment is consistent with the Raman and DFT results, which indicate progressive framework corrugation and reorganization at higher Mn loadings.

Two representative Mn loadings were selected to capture the regimes observed experimentally at low and high loading (Figure 4). From the relaxed structures, increasing Mn loading progressively perturbs the layered arrangement of the carbon nitride framework. As shown in Figure 4 (left), the low‐Mn‐loading models preserve the intrinsic layered architecture of the carbon nitride support. The Mn centers are accommodated within the framework without inducing significant distortion of the host structure, maintaining an approximately tetra‐coordinated Mn–N_4_ environment. The layers remain nearly planar and weakly coupled, with an average interlayer separation of 3.13 Å, a lateral displacement of 0.92 Å, and minimal out‐of‐plane corrugation (0.32 Å). This structural picture is consistent with EXAFS analysis (Table S1 and Figure S5), which indicates a Mn–N coordination number close to four and Mn–N distances of approximately 2.2 Å, supporting the presence of isolated Mn single‐atom sites stabilized by nitrogen ligands. In contrast, the higher Mn‐loading models shown in Figure 4 (right) exhibit substantial structural reorganization of the carbon nitride framework. The Mn centers remain atomically isolated but engage more strongly with adjacent layers, promoting interlayer bridging interactions. This rearrangement is characterized by longer Mn–N interactions (2.3 Å), indicative of stronger metal‐support bonding, together with longer Mn···C contacts at ca. 2.7 Å. The formation of these interlayer connections drives a marked distortion of the support, evidenced by the reduction of the interlayer spacing from 3.13 to 2.64 Å, the increase in lateral stacking displacement from 0.92 to 1.29 Å, and the rise in out‐of‐plane corrugation from 0.32 to 2.10 Å (Table 2). These structural changes are consistent with the Raman spectral evolution, the broadening of the XRD reflections, and the trends obtained from EXAFS fitting, indicating progressively stronger framework distortion at higher Mn concentrations.

**FIGURE 4:**
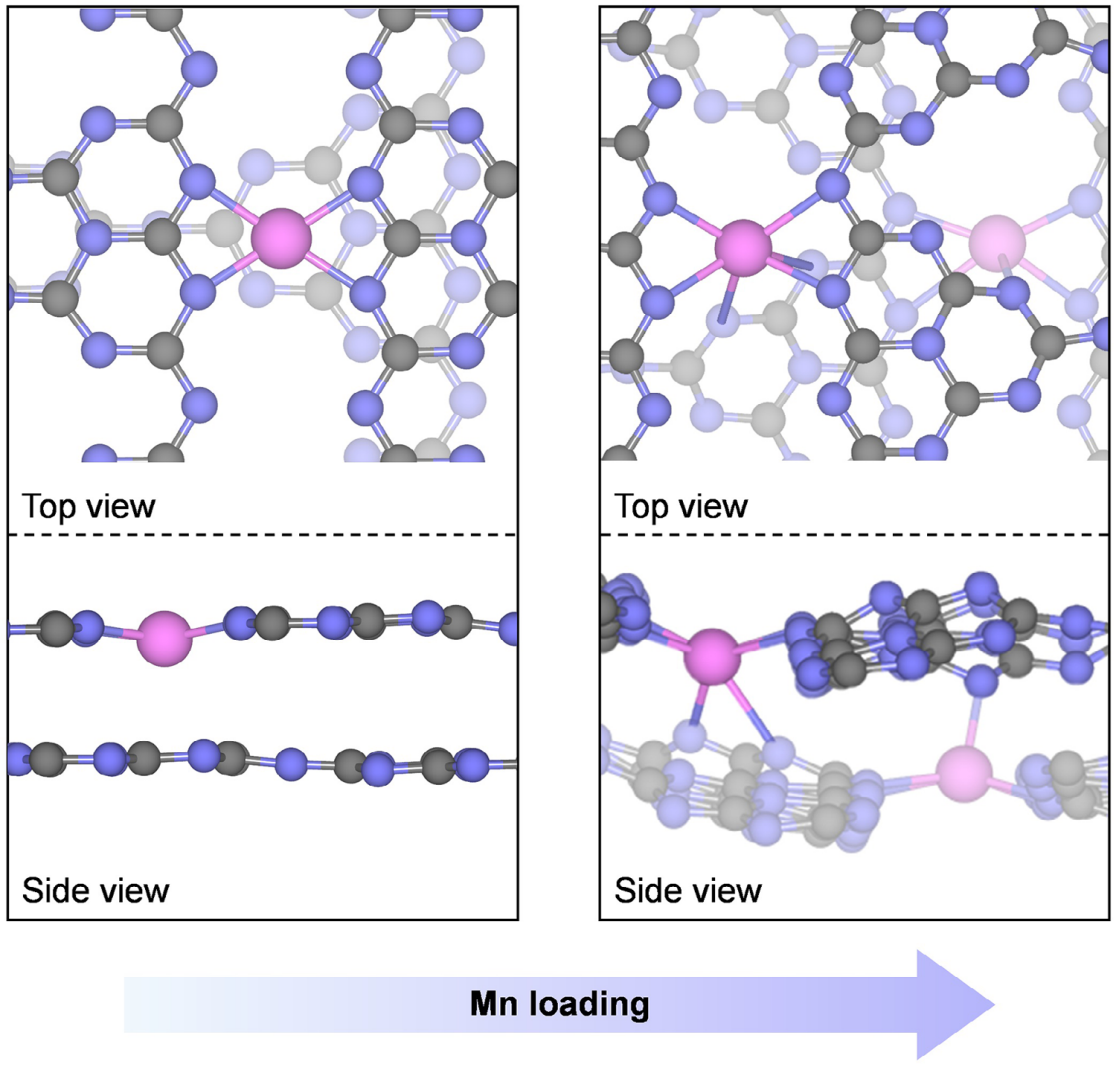
DFT‐optimized bilayer carbon nitride models used for stacking analysis at low Mn loading (left) and high Mn loading (right), shown in top and side views. Mn atoms are shown in pink, nitrogen in lilac, and carbon in grey.

**TABLE 2:** Structural parameters derived from the DFT‐optimized bilayer carbon nitride models representative of the low and high Mn loading systems shown in Figure 4.

	Left structure	Right structure
Mn–N	2.07‐2.11 Å	2.19‐2.41 Å
Mn–C	2.46‐2.48 Å	2.72‐2.73 Å
Interlayer distance^a^	3.13 Å	2.64 Å
Displacement	0.92 Å	1.29 Å
Corrugation	0.32 Å	2.10 Å

^a^ The value indicates average distance.

### 3.3 Structural Reorganization Drives an Electronic Regime Transition

We next investigated how these structural changes influence the electronic and photophysical properties of the material. Diffuse reflectance, PL, transient absorption, and X–‐ray photoelectron spectroscopies were employed to determine whether the observed structural transition is accompanied by changes in electronic disorder, charge‐carrier dynamics, and metal‐support interactions. DRS was used to estimate the optical band gaps using Tauc plots for direct allowed transitions [49]. The extracted values lie in the range of 2.86‐2.93 eV and show a monotonic increase with the Mn content (Figure S6). Interestingly, both the band gap and Urbach energy increase progressively with Mn loading (Figure 5a), in line with the XRD results, with a more pronounced rise beyond 1.00 wt.%, indicating that electronic disorder accumulates continuously but becomes dominant at higher site densities. PL spectroscopy further probed the effect of Mn incorporation on the CN*_x_* scaffold. As shown in Figure 5b, PL intensity decreases markedly with increasing Mn loading, indicating the introduction of electronic states that trap charge carriers and suppress radiative recombination [55, 56]. On the other hand, Figure 5c shows that changes in PL intensity correlate with shifts in the PL peak position, suggesting that Mn‐induced localized states influence the excited carrier dynamics. Both the optical band gap and PL peak position blue‐shift with increasing Mn content, while the Stokes shift remains nearly constant, indicating that shallow states introduced by Mn track the band‐edge shifts.

**FIGURE 5:**
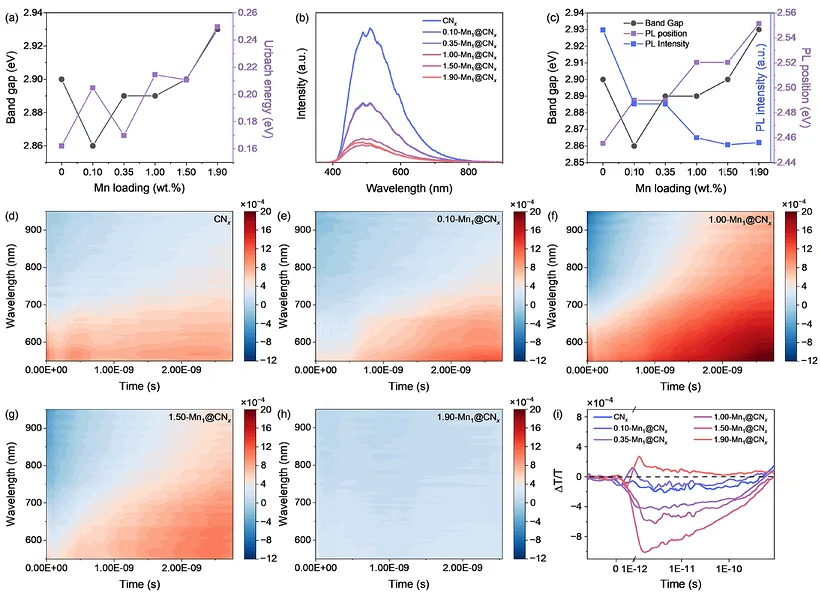
Band gaps and Urbach energies extrapolated from the absorption measurements (a), PL spectra (b), comparison of band gaps, PL position, and PL intensity (c). 2D heatmaps of transient absorption measurements up to 2.5 ns in a wavelength range between 550 and 950 nm of pristine CN_x_ (d), 0.10‐Mn_1_@CN_*x*_ (e), 1.00‐Mn_1_@CN*_x_* (f), 1.50‐Mn_1_@CN*_x_* (g), and 1.90‐Mn_1_@CN*_x_* (h). Blue shades indicate negative photoinduced signals and red shades positive photobleach signal. Transient absorption decays extrapolated from the heatmaps in a wavelength range between 900 and 950 nm (i) of selected Mn_1_@CN*_x_* catalysts.

Carrier dynamics were further investigated via TAS on acetone dispersions of the catalysts over a 550‐950 nm spectral range and 2.5 ns time scale. Pristine CN*_x_* exhibits a positive photobleach (PB) signal at shorter wavelengths (<800 nm) and a negative photoinduced absorption (PIA) at longer wavelengths (>800 nm), consistent with carriers trapped in localized states [57, 58]. Mn incorporation broadens and prolongs the PIA signal (Figure 5d–h), indicative of an increased population of trapped carriers. Figure 5i shows that increasing Mn content up to 1.50 wt.% enhances the PIA intensity, while the highest Mn loading (i.e., 1.90‐Mn_1_@CN*_x_* sample) shows a shortened PIA lifetime, which suggests that, beyond a critical density of Mn sites, trap‐assisted recombination begins to dominate over charge separation, marking a shift from beneficial to detrimental electronic perturbation [59].

The surface chemical states of the Mn species and the nitrogen environments in representative Mn_1_@CN*_x_* catalysts (i.e., 1.00, 1.50, and 1.90 wt.%) were further investigated by XPS. The Mn 2p core level spectra (Figure S7a–c) for these samples display the characteristic spin‐orbit doublet with the 2p_3/2_ component centered at around 642 eV, although for the 1.90‐Mn_1_@CN*_x_* sample this maximum was found at 640 eV due to charging effects. The Mn 2p line shape and satellite structure are consistent with predominantly Mn^2+^ at the surface. The spectral line shape provides further support, evidencing a second component at ∆E = 4.25 ± 0.25 eV, corresponding to a shake‐up satellite feature, reported to be specific for Mn^2+^ species [60, 61]. The relative intensity of this satellite decreases progressively with increasing Mn loading, suggesting a gradual evolution of the Mn surface electronic environment, consistent with the subtle changes inferred from XANES. No additional features corresponding to metallic Mn or the +3 oxidation state were detected, also suggesting the formation of isolated Mn^2+^ centers on the carbon nitride matrix. The N 1s spectra (Figure S7d) exhibit the typical components of graphitic carbon nitride materials [62, 63], with peaks at ca. 398.4 eV, associated with bridging nitrogen species from the heptazine rings (C═N–C), at ca. 400.3 eV, attributed to C–NH_2_ and C_2_–N–H species, at ca. 401.7 eV, related to N–C_3_ environments, and at 405.3 eV, which can be ascribed to satellites. Moreover, C 1s core‐level spectra (Figure S7e) show a main signal centered at around 288 eV, consistent with the fingerprint of CN*_x_*. However, both N 1s and C 1s signals are slightly broadened due to inhomogeneous surface charging, resulting from sample preparation. In the XPS spectra, a shoulder at lower binding energies is observed, increasing progressively with Mn loading and becoming particularly evident for 1.90‐Mn_1_@CN*_x_*. Although nonuniform charging effects from sample preparation may contribute to this feature, the emergence of this signal, along with the drastic loss in surface area for this sample (see Table 1) and the structural rearrangement observed by spectroscopic analyses, suggests a genuine structural rearrangement. Similar low‐binding‐energy contributions have been previously reported for carbon nitride systems undergoing interlayer reorganization and/or partial delamination, leading to modified electronic environments of the nitrogen atoms [63]. This evolution may be due to local changes in the electronic density of the nitrogen framework induced by Mn incorporation, which promote a redistribution of charge and the partial collapse of the mesoporous structure.

Overall, the optical, spectroscopic, and transient measurements reveal an electronic regime transition driven by Mn site density. At low‐to‐intermediate loadings, Mn‐induced electronic states act as productive centers that prolong carrier lifetimes and favor charge separation. Beyond approximately 1.00 wt.% Mn, continued framework distortion generates excessive disorder and promotes nonradiative recombination. Thus, the structural reorganization identified in Section 3.2 directly translates into a transition from a charge‐separation‐dominated regime to a recombination‐dominated regime.

### 3.4 Catalytic Consequences of the Site‐Density Transition

Having identified distinct structural and electronic regimes as a function of Mn site density, we evaluated their impact on the selective photocatalytic oxidation of *p*‐methoxytoluene. We first examined the effect of solvent, irradiation wavelength, reaction time, and catalyst reuse (Figure S8). These studies were initially carried out using the 0.10‐Mn_1_@CN*_x_* catalyst, as it lies within the low‐loading regime where Mn is atomically dispersed and the CN*_x_* framework retains its structural integrity, making it a representative system for evaluating intrinsic photocatalytic behavior prior to assessing loading‐dependent effects. Solvent studies revealed a strong dependence of both conversion and aldehyde yield on the reaction medium (Figure S8a). In acetonitrile, the reaction reached 50% conversion with a 36% yield of *p*‐methoxybenzaldehyde, indicating effective benzylic C–H activation; however, formation of the corresponding alcohol lowered aldehyde selectivity. In water, the conversion increased to 93%, but aldehyde selectivity decreased sharply, affording only an 8% yield. This outcome suggests that, although Mn_1_@CN*_x_* remains photoactive in water, unselective oxidation pathways dominate, likely due to the formation of highly reactive oxygen‐centered species in aqueous media [64, 65]. A 1:1 (v/v) mixture of acetonitrile and water resulted in lower conversion (20%) and low aldehyde yield (9%), reflecting poor phase compatibility. Notably, acetone provided the best balance, delivering 66% conversion with 54% aldehyde yield at the experimental conditions, suggesting that solvent polarity and oxygen solubility influence the selectivity of the oxidation process [64, 65]. Overall, these data indicate that polar aprotic solvents, such as acetone, favor selective benzylic oxidation over Mn_1_@CN*_x_*, whereas protic solvents promote overoxidation. Photocatalytic activity was also wavelength‐dependent (Figure S8b), consistent with the optical absorption profile of Mn_1_@CN*_x_*. Under violet light (395 nm), the catalyst displayed the highest performance, affording a 54% yield of *p*‐methoxybenzaldehyde. As the excitation wavelength shifted toward the blue region, the activity decreased, with yields of 43% and 36% at 420 and 457 nm, respectively. This behavior is consistent with the stronger absorption of Mn_1_@CN*_x_* in the violet region of the spectrum [66]. In contrast, the reduced yields at longer wavelengths reflect less efficient photoexcitation under lower‐energy irradiation.

The influence of reaction time was subsequently examined, revealing fast initial kinetics followed by a gradual plateau in product formation at longer irradiation times (Figure S8c). Within the first 30 min, the conversion of *p*‐methoxytoluene reached ca. 60% with a 45% yield of *p*‐methoxybenzaldehyde, indicating rapid benzylic C–H activation under violet irradiation. Extending the reaction to 60 min increased conversion to >80%, while the aldehyde yield grew only marginally to ca. 55%, suggesting that secondary oxidation processes begin to limit aldehyde accumulation [67]. At longer irradiation times, the aldehyde yield remained nearly constant, whereas conversion could continue to increase, indicating that a larger fraction of the converted substrate is directed toward secondary reaction products. This interpretation is consistent with chromatographic analyses showing the appearance of additional product peaks at extended reaction times. Based on this kinetic profile, a reaction time of 45 min was selected for comparing catalytic performance across the Mn‐loading series. To evaluate the stability and recyclability of the Mn‐based catalyst, we conducted recyclability experiments over five consecutive reaction cycles (Figure S8d). The catalyst demonstrated robust recyclability with negligible loss in activity. In terms of intrinsic activity, the turnover frequency (TOF) of 0.10‐Mn_1_@CN*_x_* (633 h^−1^) exceeds those of previously reported photocatalysts for selective benzylic C–H oxidation by approximately one order of magnitude (Table S2), reflecting the high efficiency of isolated Mn sites in this system.

With the conditions identified, control experiments confirmed that the observed oxidation indeed arises from a synergistic photocatalytic process involving Mn single atoms, the carbon nitride support, and molecular oxygen (Table S3). In the absence of catalyst, no product formation was detected, ruling out uncatalyzed photooxidation of *p*‐methoxytoluene. Likewise, reactions performed in the dark yielded negligible conversion, demonstrating that light is essential for C–H activation. Performing the photooxidation in air resulted in lower conversion and aldehyde yield, consistent with the reduced oxygen availability under these conditions and confirming the important role of molecular oxygen in the oxidation process. Pristine CN*_x_* markedly exhibited lower activity under identical conditions, highlighting the critical role of atomically dispersed Mn centers in enhancing both photocatalytic efficiency and selectivity [68]. Overall, the results confirm that selective oxidation requires the combined action of light, O_2_, and the Mn_1_@CN*_x_* catalyst. Following the identification of appropriate reaction conditions, the applicability of the 0.10‐Mn_1_@CN*_x_* photocatalyst was evaluated in the aerobic oxidation of structurally diverse benzylic substrates (Figure 6). The reaction proceeds efficiently for electron‐rich aromatic substrates, whereas lower yields are generally obtained for electron‐deficient systems. In particular, substrates containing electron‐donating substituents such as methoxy or tert‐butyl groups afforded the corresponding aldehydes in moderate isolated yields, while halogenated, cyano‐, and nitro‐substituted derivatives showed lower reactivity. Ethylbenzene and fused aromatic substrates were also successfully oxidized. Polyfunctionalized substrates, however, showed lower yields, likely due to competing overoxidation processes under the reaction conditions. The isolated yields exhibit a negative linear correlation with substituent electronegativity (*R* ^2^ = 0.7944), indicating that electron‐rich benzylic substrates are more readily oxidized under the investigated conditions. This trend is consistent with the mechanistic results discussed above, where electron transfer and oxygen activation are favored in substrates with higher electron density at the benzylic position.

**FIGURE 6:**
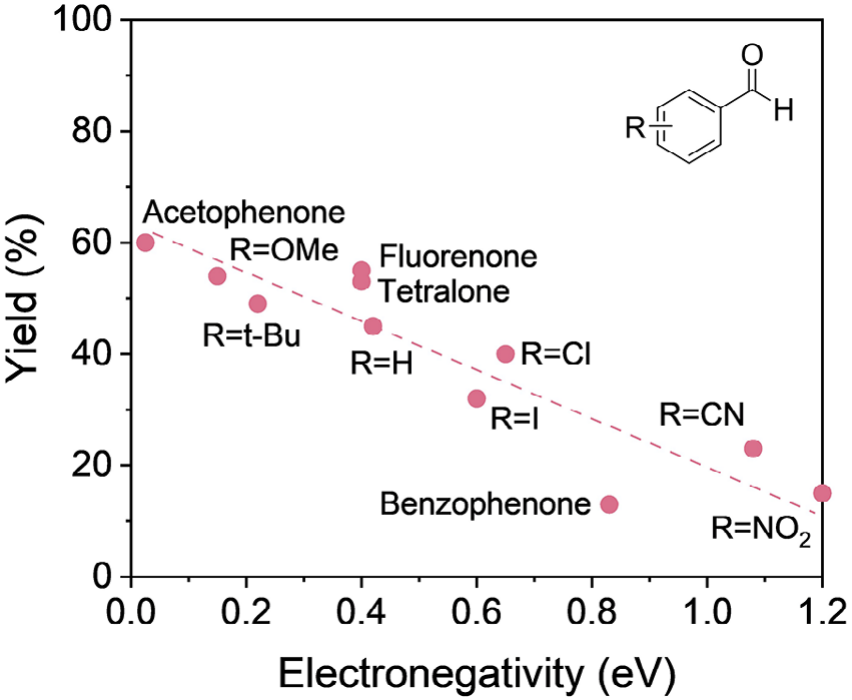
Compound compatibility and electronic effects in the photocatalytic oxidation of benzylic C–H bonds to aldehydes. Yields of the corresponding products are plotted as a function of the substituent electronegativity (eV), showing a negative linear correlation (*R* ^2^ = 0.7944). Reaction conditions: substrate (0.08 mmol), acetone (1 mL), O_2_ atmosphere, 25°C, using 0.10‐Mn_1_@CN_*x*_ (5 mg), under irradiation at 395 nm. Reported values correspond to isolated yields. The general procedure and the characterization of the isolated products are described in the Supporting Information.

Reaction scalability in flow mode was also investigated (Figure S9), and, under continuous‐flow conditions using an immobilized Mn_1_@CN*_x_* thin film, *p*‐methoxybenzaldehyde was obtained in 72% yield after a 45 min residence time, outperforming the corresponding batch reaction. Homogeneous catalyst films prepared by drop‐casting (Figures S10 and S11) were incorporated into a custom‐built microchannel reactor (Figure S12). The immobilized catalyst also showed stable performance during continuous operation and repeated reuse experiments.

Having established the reaction conditions and confirmed the photocatalytic origin of the transformation, we next investigated how the site‐density‐induced structural and electronic transitions identified in Sections 3.2 and 3.3 influence catalytic performance (Figures 7 and S13). Conversion and aldehyde yield were measured at 45, 90, and 120 min. A clear trend emerges across the series regardless of the reaction time: catalysts with Mn loading below 1.00 wt.% deliver the highest conversions and yields, whereas samples with Mn > 1.00 wt.% exhibit a decline in activity, approaching negligible catalytic activity for 1.90‐Mn_1_@CN*_x_*. For the 45‐min reactions (Figure 7a), catalysts with < 1.00 wt.% Mn display conversions in the 50%‐60% range and aldehyde yield of 40%‐50%, with 0.35‐Mn_1_@CN*_x_* achieving the best balance (50% conversion, 50% yield). Increasing the reaction time to 90 min (Figure 7b) raises conversions to 75%‐90% and yields to 50%‐60% for the most active samples. At 120 min (Figure 7c), aldehyde yields remain largely unchanged, while conversion increases due to the formation of secondary oxidation products. Although 0.35‐Mn_1_@CN*_x_* shows the highest conversion and yield in absolute terms, 0.10‐Mn_1_@CN*_x_* exhibits the highest TOF (633 h^−1^), making it the most intrinsically active catalyst in the series (Figure 7d). Moreover, the loss of activity above 1.00 wt.% Mn mirrors both the structural reorganization (Section 3.2) and the transition toward recombination‐dominated carrier dynamics (Section 3.3), indicating that support adaptation rather than metal aggregation governs the catalytic response. This nonmonotonic response distinguishes the system from conventional loading‐dependent catalysis: increasing Mn content first improves the density of accessible active sites, but beyond the threshold the adaptive restructuring of the support overrides this benefit and switches the catalyst into a low‐efficiency regime [5, 7]. Additional normalization of the catalytic activity by BET surface area and optical absorption at 395 nm yielded the same overall trend (Figure S14), further supporting that the decrease in performance at higher Mn loadings is not primarily governed by textural and/or optical effects.

**FIGURE 7:**
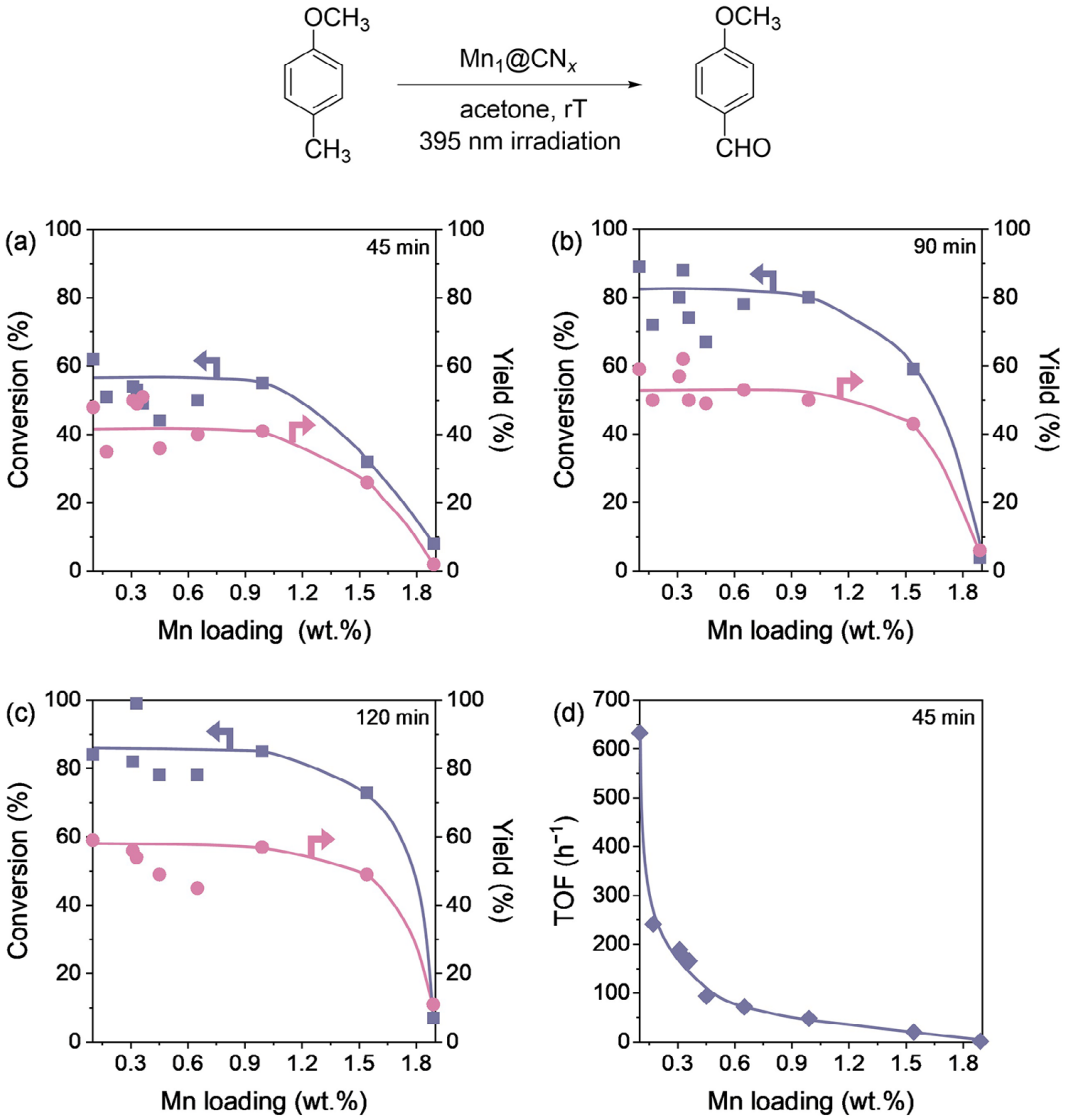
Catalytic performance of Mn_1_@CN*_x_* samples as a function of Mn loading at 45 min (a), 90 min (b), and 120 min (c) of reaction time. TOFs of Mn_1_@CN*_x_* samples after 45 min of reaction time (d). Reaction conditions: p‐methoxytoluene (10 µL), acetone (1 mL), Mn_1_@CN*_x_* catalyst (5 mg), O_2_ atmosphere, 25°C, and irradiation at 395 nm.

### 3.5 Mechanistic Origin of the Productive and Inactive Regimes

To elucidate the nature of the active oxidizing species involved in the photocatalytic benzylic oxidation, a series of scavenger experiments was conducted using selective quenchers of reactive oxygen species (reactive oxygen species, ROS [69]). The addition of benzoquinone markedly suppressed both yield (from 55% to 37%) and selectivity (from 74% to 58%), providing evidence consistent with the participation of oxygen‐derived reactive species in the electron‐transfer pathway driving benzylic oxidation. Likewise, furfuryl alcohol led to an even stronger inhibition of activity, with the yield dropping to 15% and selectivity dropping to 40%, further supporting that oxygen‐derived excited species contribute significantly to substrate conversion. Addition of isopropanol reduced the aldehyde yield to 14% and selectivity to 34%, consistent with the highly reactive and nonselective nature of oxygen‐derived oxidizing species, which promotes unproductive degradation of both substrate and product. Similarly, addition of ascorbic acid decreased the aldehyde yield to 26% and selectivity to 57%, indicating that photogenerated charge carriers play an important role in sustaining the oxidation process. These mechanistic insights are representative of the low‐loading regime of Mn_1_@CN*_x_*. In this regime, the catalyst adapts favorably to Mn incorporation by stabilizing isolated Mn–N*_x_* centers while maintaining a trap‐state landscape that supports productive charge separation and oxygen activation. TAS measurements also support this picture, showing the formation of longer‐lived photoinduced states at low‐to‐intermediate Mn loadings, consistent with improved charge separation and enhanced electron transfer toward adsorbed oxygen species. In this state, the isolated Mn–N*_x_* sites and the preserved monolayer‐like CN*_x_* framework facilitate the selective generation of oxygen‐derived reactive species. Beyond the site‐density threshold, this adaptive response becomes detrimental: framework reorganization and increased electronic disorder interfere with the energy‐ and electron‐transfer processes required for selective ROS generation, providing a possible explanation for the sharp decline in catalytic performance observed at higher loadings.

To further investigate the role of Mn single atoms in oxygen activation and ROS formation, DFT calculations were performed on the monolayer SAC model representative of the low‐loading regime, where the local Mn coordination environment remains preserved (Figure 8). The proposed catalytic cycle and the corresponding free energy surface (FES) diagram are shown in Figure 8a,b, respectively.

**FIGURE 8:**
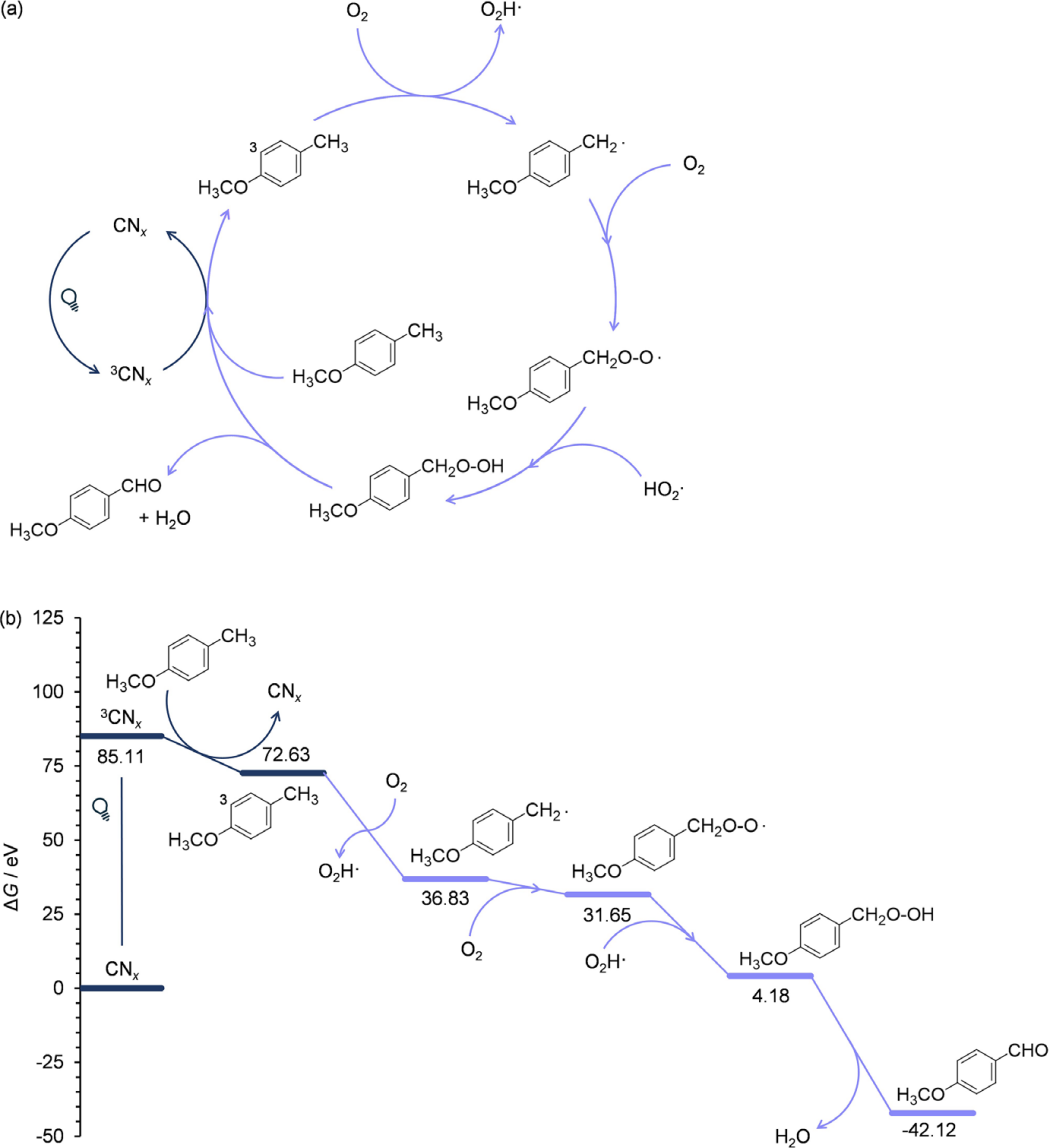
Proposed catalytic cycle (a) and free energy diagram (b) for the photocatalytic oxidation mechanism over Mn_1_@CN*_x_*.

The initiation step involves photoexcitation of the metal‐free carbon nitride (CN*_x_*) from its singlet ground state to its triplet state (^3^CN*_x_*) through the photoreaction R1 (Δ*G* = 85.11 kcal mol^−1^), which provides the energy required to sustain the system reactivity. This is consistent with the experimental observation that the reaction does not proceed in the absence of photoexcitation (Table S3, entry 4). The ^3^CN*_x_* state is the lowest‐energy triplet excited state of CN*_x_*. Its formation is consistent with previous experimental evidence [70] and, given the longer lifetime with respect to singlet states, it is reasonable to assume that it plays a key role in initiating the catalytic process. As the scavenger experiments suggest that reactive oxygen species (ROS) have an active contribution in the reaction, their possible contribution to the oxidation of *p*‐methoxytoluene, here labeled as Ar–CH_3_, was initially investigated through the following reaction cycle:

(R1)

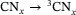



(R2a)

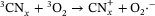

(R3a) $$O_{2}\cdot^{-}+\mathrm{Ar}\text{--}{\mathrm{CH}}_{3}\to O_{2}H^{-}+\mathrm{Ar}\text{--}{\mathrm{CH}}_{2}\cdot$$

(R3b)

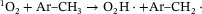




The calculations show that ^3^CN*_x_* is able to activate molecular oxygen to form O_2_•^–^ (Δ*G* = –0.65 kcal mol^−1^, R2a). However, hydrogen abstraction from the aromatic substrate is thermodynamically unfavorable (Δ*G* = +28.70 kcal mol^−1^, R3a). Along with the superoxide formation, ^1^O_2_ may also be generated under irradiation. Nevertheless, hydrogen abstraction by singlet oxygen is energetically unfavorable (Δ*G* = +17.96 kcal mol^−1^, R3b). We therefore investigated whether substrate activation through an energy transfer mechanism could promote oxidation, finding that the process is exothermic (Δ*G* = –12.49 kcal mol^−1^, R2b):
(R2b)

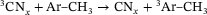




Once formed, triplet‐state *p*‐methoxytoluene readily undergoes hydrogen abstraction by oxygen (Δ*G* = –35.79 kcal mol^−1^, R3c), producing the benzyl Ar–CH_2_• radical and O_2_H•:

(R3c)

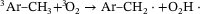




Reactions R1, R2b, and R3c therefore constitute a thermodynamically favorable sequence that explains the initiation of *p*‐methoxytoluene oxidation up to the formation of the benzylic radical. Interestingly, this mechanism is also consistent with the observed effect of furfuryl alcohol, widely used as a singlet oxygen scavenger but also reported to quench excited triplet states [70, 71].

Once formed, the benzyl Ar–CH_2_• radical rapidly reacts with oxygen to generate a peroxyl radical (Δ*G* = –5.19 kcal mol^−1^, R4), which subsequently reacts with O_2_H• to form the Ar–CH_2_OOH intermediate and oxygen (Δ*G* = –27.49 kcal mol^−1^, R5):

(R4)

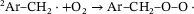



(R5)

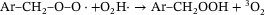




The final step of the reaction sequence is the dehydration of Ar–CH_2_OOH to form the aldehyde product (Δ*G* = –46.29 kcal mol^−1^, R6):

(R6) $$\mathrm{Ar}\text{--}{\mathrm{CH}}_{2}\mathrm{OOH}\to \mathrm{Ar}\text{--}\mathrm{CHO}+H_{2}O$$

This step is likely to be the one most strongly influenced by synergy with the Mn single‐atom catalyst. Indeed, Mn^2+^ species are known to react with O_2_H• [72], generating additional ROS, including O_2_•^–^. It is therefore reasonable to propose that ROS contribute to the oxidation of the hydroperoxide intermediate, thereby facilitating conversion to the aldehyde product.

The proposed reaction mechanism provides a molecular‐level explanation for the experimentally observed decrease in activity with increasing Mn loading. As shown in Figure 9, the catalytic cycle is initiated by excitation of CN*_x_* from the singlet ground state (S_0_) to the excited triplet state (T_1_), followed by a triplet‐triplet energy transfer to the substrate. For this process to be energetically favorable, the T_1_/S_0_ excitation energy of CN*_x_* must be higher than that of the substrate, and the T_1_ lifetime must be sufficiently long to allow energy transfer to occur. However, increasing the Mn loading activates two parallel, competing mechanisms. The first is a decrease in the lifetime of the photoexcited state caused by the presence of a Mn atom in the vicinity of the photocatalytic center. When incorporated into the CN*_x_* matrix, Mn leads to closely spaced high‐spin states. Indeed, in the Mn‐melem dimer used to model the catalyst at high Mn loadings, the minimum energy spin state is a quartet, with the doublet being 37 kcal mol^−1^ and the sextet 1 kcal mol^−1^ higher in energy, respectively. These relatively small energy gaps imply that, following photoexcitation of CN*_x_* in the presence of a Mn atom, the excited state lies significantly higher in energy than the corresponding ground spin state, and that the different spin states are efficiently coupled through spin‐state accessibility. Consequently, photoexcitation provides access to multiple nonradiative relaxation pathways in which the electronic excitation energy is dissipated through internal conversion followed by thermal relaxation, thereby shortening the excited‐state lifetime and reducing the efficiency of energy transfer to the substrate. In contrast, at low Mn loadings, the lifetime of the ^3^CN*_x_* T_1_ state is expected to be longer because it represents the lowest‐energy excited state of the system. Its principal deactivation pathways are either intersystem crossing to singlet states, which is spin‐forbidden and therefore slow, or energy transfer to the substrate. The second aspect to be considered originates from a decrease in the T_1_–S_0_ energy gap caused by the structural reorganization of the catalyst at high Mn loadings. It is reasonable that a distortion of the planar structure of CN*_x_*, as observed in the periodic DFT simulations, reduces the stability of the singlet state with respect to the triplet state. To measure the extent by which a structural deformation can affect the T_1_–S_0_ energy gap, we performed simulations using the plane wave bilayer atomic model shown in Figure 4 and atom‐centered Gaussian basis sets simulations. It is found that the energy gap decreases significantly from 85.1 to 31.1 kcal mol^−1^, thus making the energy transfer to the substrate energetically unfavorable. While the adopted molecular model is representative of high loading conditions, it can be reasonably assumed that a smaller loading, leading to a more limited structural reorganization, may be sufficient to decrease the T_1_‐S_0_ energy gap enough to make the energy transfer to the substrate unfavorable. Overall, the combined scavenger, TAS, and DFT results support a mechanism in which isolated Mn centers promote oxygen activation and ROS generation, while the carbon nitride framework sustains photoinduced charge separation under irradiation. Only within the low‐loading regime, this interplay promotes selective benzylic oxidation via oxygen activation pathways [73]. Increasing Mn incorporation drives a transition toward a high‐loading regime characterized by reduced T_1_–S_0_ excitation energies and enhanced nonradiative relaxation pathways, both of which diminish the efficiency of substrate activation.

**FIGURE 9:**
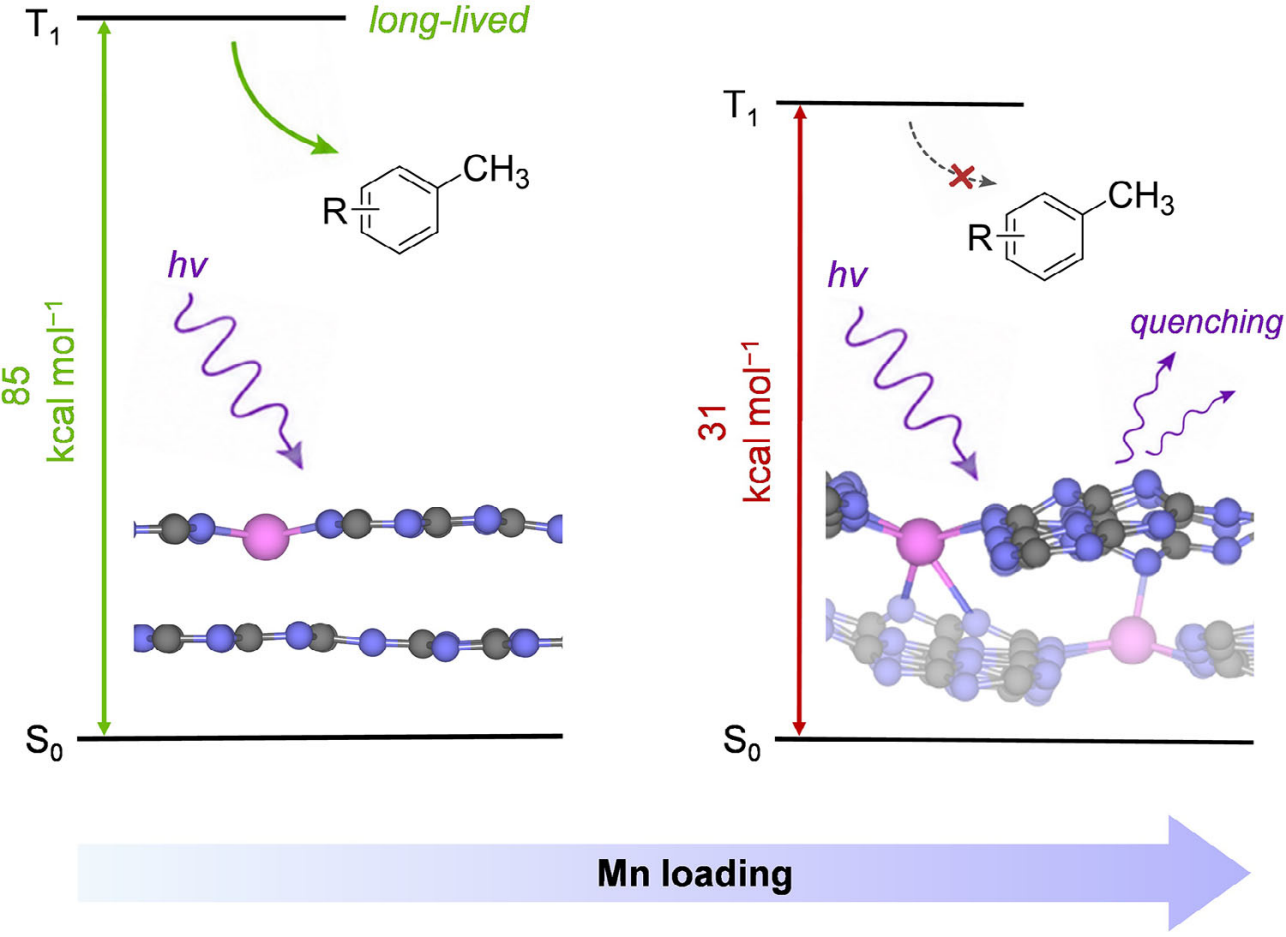
Mechanistic illustration of the adaptive response of Mn_1_@CN*_x_* to increasing single‐atom site density. At low Mn loadings, the carbon nitride support remains weakly stacked and preserves a long‐lived, high‐energy triplet excited state (T_1_–S_0_ = 85 kcal mol^−1^), enabling efficient energy transfer to the substrate and selective benzylic oxidation. Above a critical Mn loading, support reorganization decreases the T_1_–S_0_ energy gap (31 kcal mol^−1^) and promotes nonradiative deactivation pathways through Mn‐induced electronic states. The resulting loss of triplet‐state reactivity suppresses substrate activation and drives a transition from a highly active to a low‐efficiency photocatalytic regime.

## 4 Conclusion

This work establishes Mn site density as a structural and photophysical design parameter in carbon nitride‐supported single‐atom photocatalysts. By systematically varying Mn loading while maintaining atomic dispersion, we show that the number of isolated metal sites does not simply determine the density of active centers; instead, it controls the organization of the CN*_x_* host framework and the electronic landscape that governs photocatalytic function. At low‐to‐intermediate loadings, Mn–N*_x_* sites are accommodated within a partially delaminated CN*_x_* architecture, where productive carrier trapping, preserved porosity, and favorable metal‐support interactions enable efficient oxygen activation and selective aerobic benzylic C–H oxidation. Above a critical site density, however, further Mn incorporation drives framework densification, interlayer reorganization, increased electronic disorder, and recombination‐dominated carrier dynamics. This transition sharply suppresses catalytic activity despite the continued absence of detectable Mn aggregation. The nonmonotonic catalytic response demonstrates that maximizing single‐atom loading is not necessarily beneficial in structurally adaptive supports. Mechanistic experiments and theoretical calculations support a regime‐dependent pathway in which isolated Mn centers and the CN*_x_* framework cooperate to generate reactive oxygen species and promote carbonyl formation only when the support maintains a favorable excited‐state landscape. Once support reorganization disrupts this balance, additional isolated Mn sites become detrimental rather than beneficial. More broadly, these findings reveal that support restructuring can precede metal aggregation and become the dominant factor controlling performance in polymer‐supported SACs.

## Author Contributions

VV was responsible for catalyst preparation and performed the mechanistic experiments, substrate scope, and flow catalytic reactions. AA was responsible for catalyst preparation and characterization and performed time and loading dependent catalytic experiments. AM carried out optimization studies. AO and AP performed the TAS, DRS and PL experiments. BPR and JLS contributed through Raman analyses. EI and CC conducted DFT calculations. ISK performed XAS. AT and VDR contributed by performing XPS analyses. AR and RA carried out STEM measurements. GV coordinated and supervised the project. The manuscript was written through contributions of all authors. All authors have given approval to the final version of the manuscript.

## Conflicts of Interest

The authors declare no conflicts of interest.

## Supporting information



Additional characterization and catalytic results of the materials; EXAFS fitting results; additional DFT calculations; continuous flow procedure; and NMR characterizations of the reaction products.**Supporting File**: anie74796‐sup‐0001‐SuppMat.pdf.

## Data Availability

The data that support the findings of this study are available from the corresponding author upon reasonable request.
