# Supplementary material for: Site Density Governs Switching Reactivity Regimes During Aerobic C─H Oxidation in Mn Single‐Atom Photocatalysts
Source: Angew Chem Int Ed Engl. 2026 Sep 24;65(46):e3256491. doi: 10.1002/anie.3256491 (PMC13749291; doi:10.1002/anie.3256491)
Supplement: Supplementary file 1 [file ANIE-65-e3256491-s001.pdf]

Supporting Information

Site Density Governs Switching Reactivity Regimes During Aerobic C–H Oxidation in Mn Single-Atom Photocatalysts

Viktoria Velichko,[a,†] Agustín de Arriba,[a,†] Areti Moutsiou,[a] Elisabetta Inico,[a] Andrea Olivati,[b] Berta Pérez-Román[c], Jesús López-Sánchez,[c] Ik Seon Kwon,[d] Andrea Tonelli,[e,f] Abhijit Roy,[g,h] Valentina De Renzi,[e,f] Raul Arenal,[g,h,i] Annamaria Petrozza,[b] Carlo Cavallotti,[a] and Gianvito Vilé,[a]*

[a] *Department of Chemistry, Materials, and Chemical Engineering "Giulio Natta", Politecnico di Milano, Piazza Leonardo da Vinci 32, 20133 Milano, Italy.*

[b] *Center for Nanoscience and Technology, Istituto Italiano di Tecnologia, Via Raffaele Rubattino 81, 20134 Milano, Italy.*

[c] *Electroceramics Department, Institute of Ceramics and Glass (ICV-CSIC), Consejo Superior de Investigaciones Científicas, Calle Kelsen 5, 28049 Madrid, Spain.*

[d] *Department of Energy Science and Engineering, Kunsan National University, 558 Daehak-ro, Gunsan-si, Republic of Korea.*

[e] *Department of Physical, Computer and Mathematical Sciences, University of Modena and Reggio Emilia, Via Campi 213a, 41125 Modena, Italy.*

[f] *Nanoscience Institute, National Research Council (CNR-NANO), 41125 Modena, Italy.*

[g] *Institute of Nanoscience and Materials of Aragón, CSIC-University of Zaragoza, Calle Pedro Cerbuna 12, 50009 Zaragoza, Spain.*

[h] *Advanced Microscopy Laboratory (LMA), University of Zaragoza, Calle Mariano Esquillor, 50018 Zaragoza, Spain.*

[i] *Aragonese Agency for Research and Development Foundation (ARAID), 50018 Zaragoza, Spain.*

† *Viktoria Velichko and Agustín de Arriba contributed equally to this work.*

* *Corresponding author. E-mail: gianvito.vile@polimi.it (Gianvito Vilé).*

General Information

Unless otherwise noted, the reagents and solvents were purchased from commercial suppliers and used without further purification. Also, unless specified, the reactions were performed without inert gas or anhydrous solvent, using a commercial PhotoCube™ reactor (ThalesNano Inc.), equipped with LEDs, operating at 395 nm, magnetic stirring, and a cooling system to control the reaction temperature. Thin layer chromatography (TLC) was performed on pre-coated silica gel 60 F254 plates (Merck) and visualized using UV-light. Organic solutions were concentrated under reduced pressure on a Büchi rotary evaporator using a water bath. The crude products were analyzed via high-performance liquid chromatography (HPLC). The conversion of the limiting reagent was deduced using an Agilent 1200 instrument, with an ultraviolet-visible detector (G1315D) set at 210 nm. A 50 µL aliquot of the filtrate was diluted in 650 µL of acetonitrile before injection. Samples and standards (5 µL) were injected directly into a 250 mm × 4.6 mm Hypersil GOLD™ aQ 5 µm 175 Å column (Thermo Scientific). The mobile phase was comprised of a 60:40 (v:v) acetonitrile:water mixture, with a total flow rate of 0.7 mL min^{-1} at 40 °C. Product identification and quantification were carried out after calibration with commercial standards of *p*-methoxytoluene, *p*-methoxybenzaldehyde, and *p*-anisic acid. The turnover frequency (TOF) was calculated according to the following equation:

$$TOF = \frac{n_{product}}{n_{Mn} \cdot t} \qquad \text{(Equation S1)}$$

where $n_{product}$ is the amount of aldehyde formed, n_{Mn} is the amount of Mn determined by ICP-OES, and t is the reaction time. The calculation assumes that all Mn atoms quantified by ICP-OES are potentially accessible and catalytically active. Because the actual number of active Mn sites is not easily quantifiable experimentally in single-atom catalysts, the use of the total Mn loading represents an upper-bound estimate of the number of catalytic sites and, consequently, yields a conservative lower-bound estimate of the apparent TOF. If only a lower fraction of the total Mn sites is catalytically active, the corresponding site-normalized intrinsic TOF would necessarily be higher than the reported value. Importantly, the same normalization approach was applied consistently across the entire catalyst series; therefore, the reported TOF values provide an internally consistent and conservative basis for comparing the relative catalytic performance of the different catalysts. The BET-normalized reaction rate was calculated according to:

$$r_{BET} = \frac{n_{product}}{t \cdot m_{cat} \cdot S_{BET}} \quad \text{(Equation S2)}$$

where m_{cat} is the catalyst mass and S_{BET} is the specific surface area. The influence of light absorption at the irradiation wavelength employed for photocatalysis (395 nm) was examined by normalizing the aldehyde yield according to:

$$Y_{395} = \frac{Yield}{A_{395}} \quad \text{(Equation S3)}$$

where A_{395} corresponds to the value extracted from the diffuse reflectance spectra at 3.14 eV (Figure S6a), corresponding to the irradiation wavelength used for photocatalysis (395 nm). NMR measurements of the isolated products were carried out using a 400 MHz Bruker AVANCE II with a BBO probe at 298 K. Chemical shifts are given in ppm relative to the residual solvent peak (^{1}H-NMR δ: $CDCl_3$ = 7.26. ^{13}C-NMR δ: $CDCl_3$ = 77.16) and all coupling constants (J) are given in Hertz (Hz) with their multiplicity: (s = singlet, d = doublet, t = triplet, q = quartet, p = quintet, m = multiplet, dd = doublet of doublets, dt = doublet of triplets, br = broad).

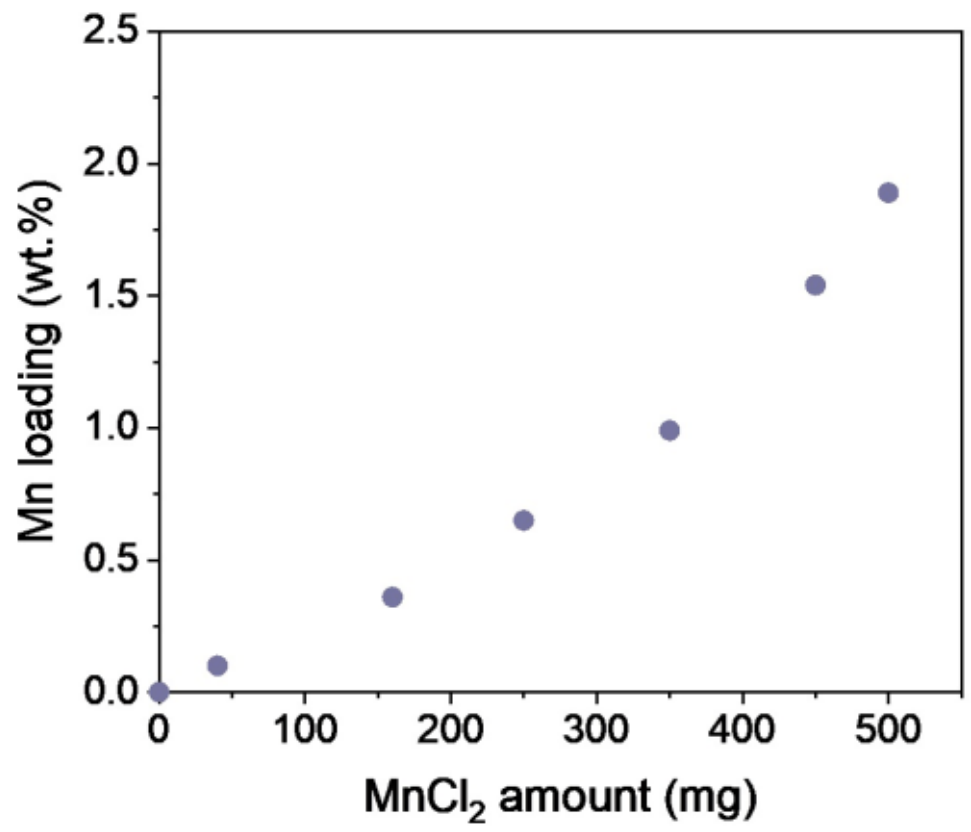

Figure S1. Metal loading determined by ICP-OES in the prepared catalysts as a function of the $MnCl_2$ precursor amount used during catalyst preparation.

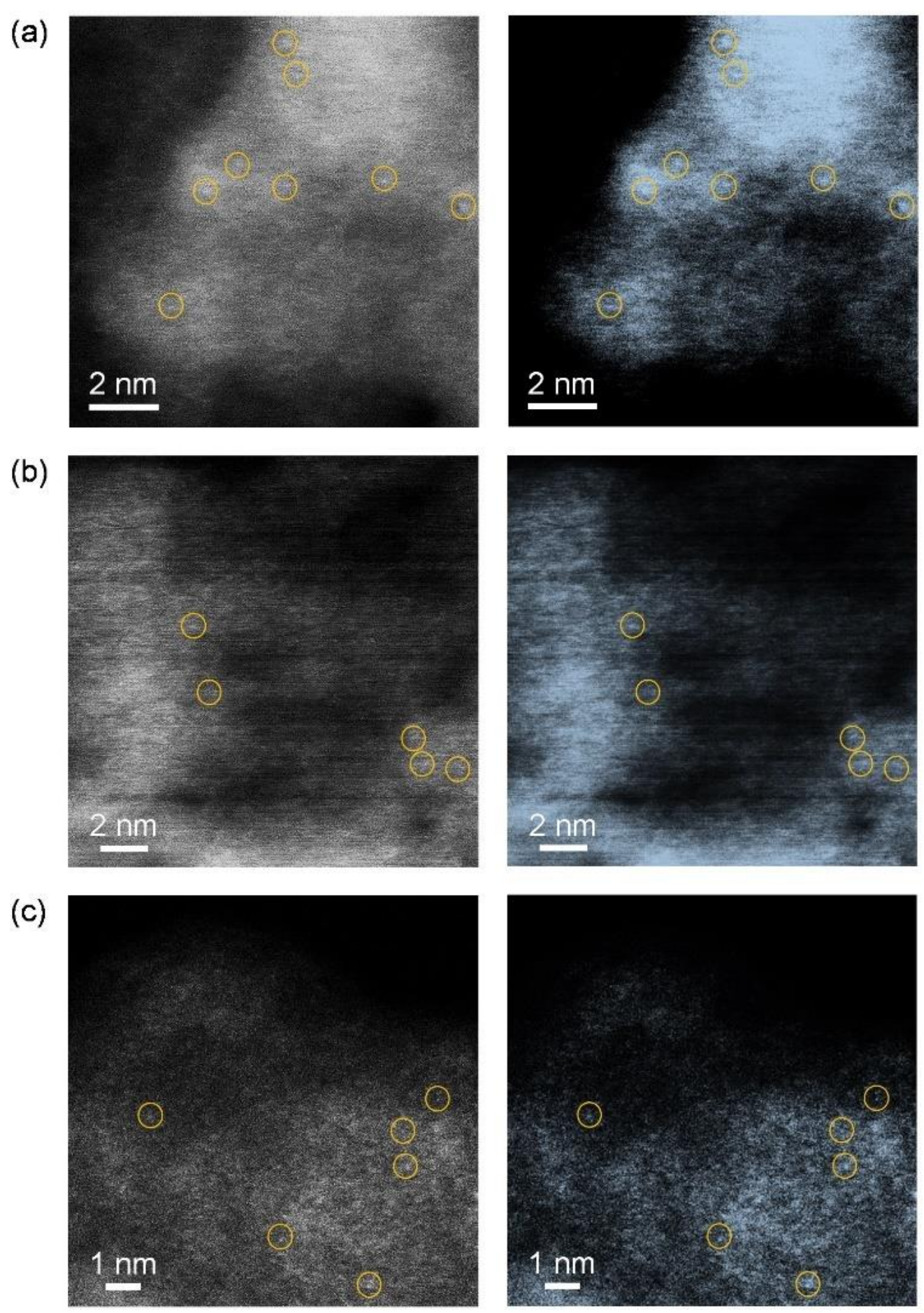

Figure S2. Additional HAADF-STEM images acquired from different regions of (a) 0.35-Mn_1@CN_x, (b) 1.00-Mn_1@CN_x, and (c) 1.90-Mn_1@CN_x. The images on the right correspond to processed versions of the original micrographs (left) to facilitate visualization of representative bright features, highlighted by yellow circles.

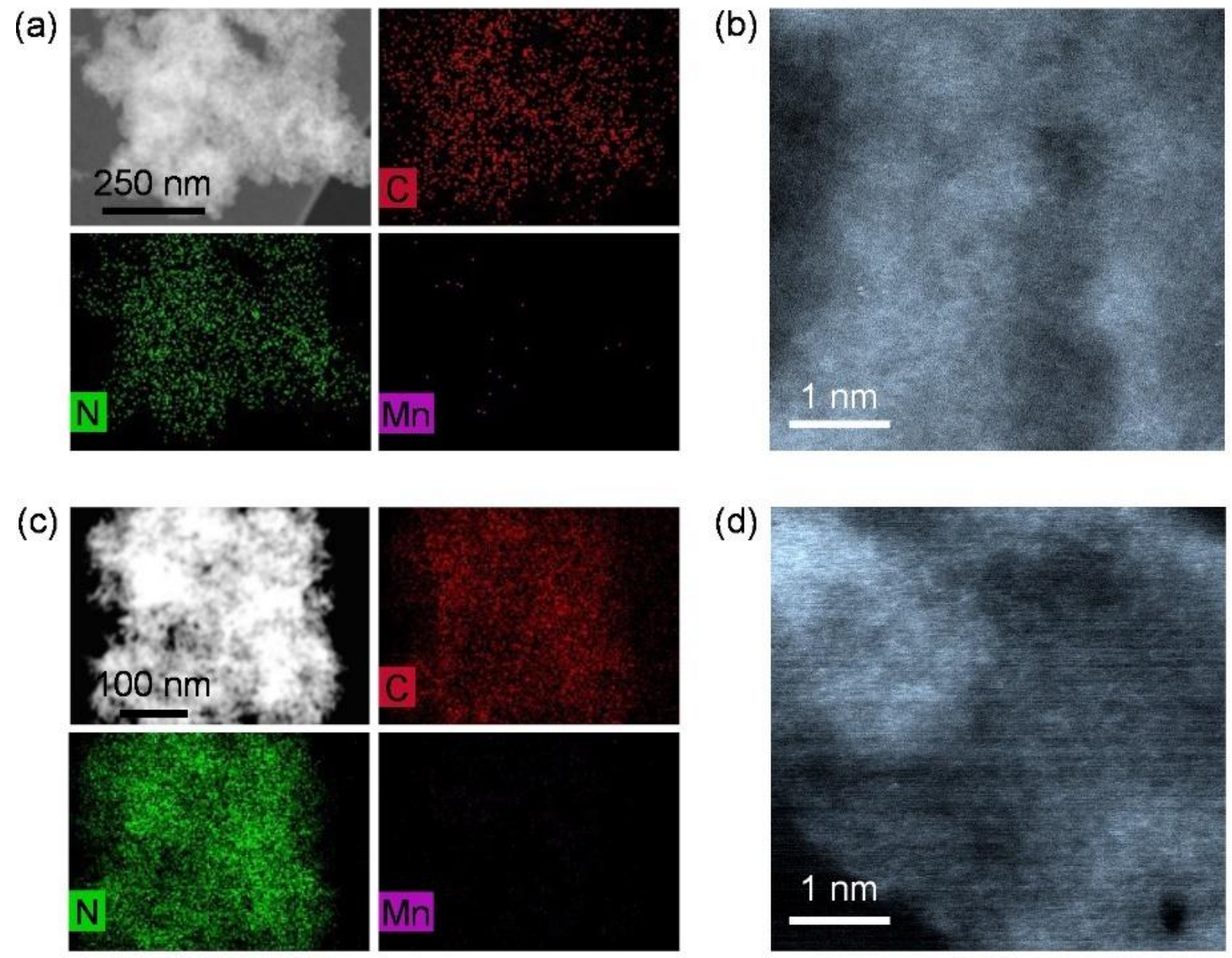

Figure S3. HAADF-STEM image and corresponding elemental maps (C, N, Mn) (a) and high-resolution HAADF-STEM micrograph showing atomically dispersed Mn sites (b) of 0.35-Mn_1@CN_x. HAADF-STEM image and corresponding elemental maps (C, N, Mn) (c) and high-resolution HAADF-STEM micrograph showing atomically dispersed Mn sites (d) of 1.00-Mn_1@CN_x.

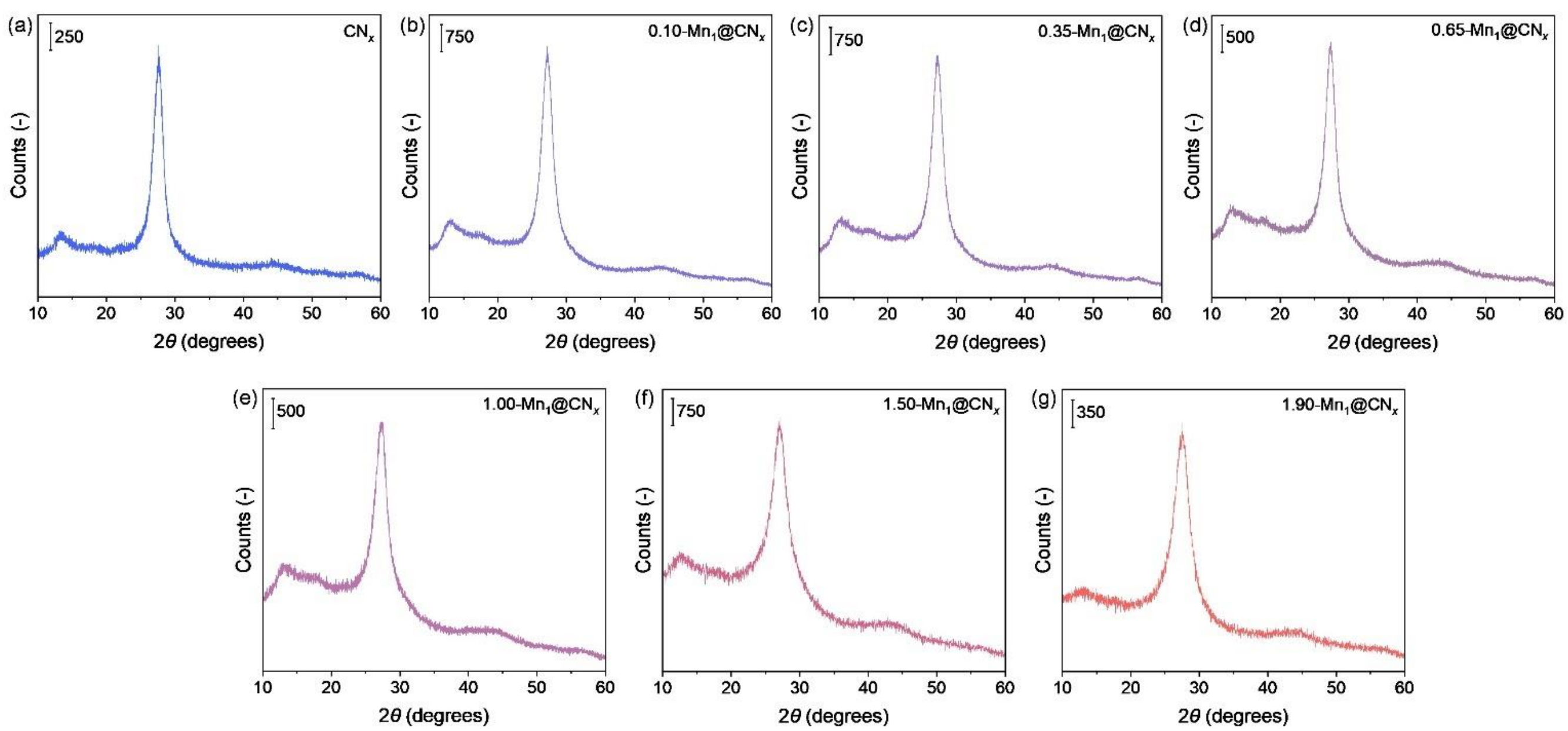

Figure S4. X-ray diffractograms of pristine CN_x (a), 0.10-Mn_1@CN_x (b), 0.35-Mn_1@CN_x (c), 0.65-Mn_1@CN_x (d), 1.00-Mn_1@CN_x (e), 1.50-Mn_1@CN_x (f), and 1.90-Mn_1@CN_x (g).

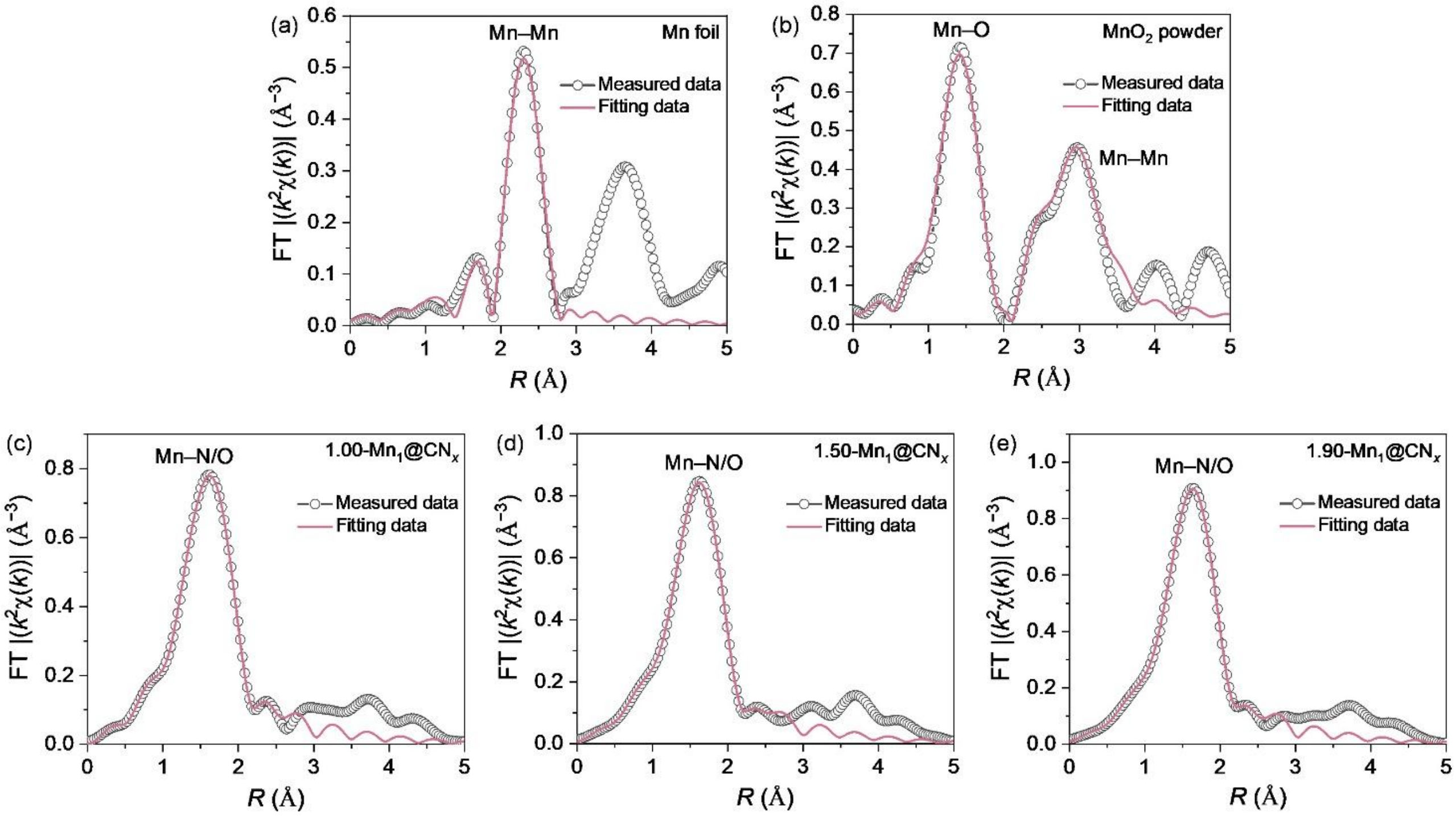

Figure S5. EXAFS fitting curves of Mn foil (a), MnO_2 powder (b), 1.00-Mn_1@CN_x (c), 1.50-Mn_1@CN_x (d), and 1.90-Mn_1@CN_x (e).

Table S1. Structural information derived from EXAFS fitting of Mn foil, MnO_2, 1.00-Mn_1@CN_x, 1.50-Mn_1@CN_x, and 1.90-Mn_1@CN_x.

Sample	Scattering path	N^a	R^b (Å)	σ^{2c} (10^{-3} Å^2)	$\Delta E_0{}^d$ (eV)	R-factor^e
Mn foil	Mn–Mn(1)	3.0	2.423±0.021	20.1±4.94	0.74±2.51	0.012
	Mn–Mn(2)	3.0	2.634±0.019	6.14±2.69		
MnO_2	Mn–O	6.0	1.880±0.013	1.23±0.96	1.53±1.75	0.008
	Mn–Mn(1)	4.0	2.923±0.023	5.87±2.30		
	Mn–Mn(2)	4.0	3.452±0.019	2.56±1.19		
1.00-Mn_1@CN_x	Mn–N(1)	2.0	2.134±0.025	5.89±2.70	0.84±0.76	0.002
	Mn–N(2)	1.9	2.265±0.010	6.48±3.24		
	Mn–C(1)	1.7	2.502±0.011	7.66±3.78		
	Mn–C(2)	1.9	2.738±0.032	8.25±4.05		
1.50-Mn_1@CN_x	Mn–N(1)	1.5	2.130±0.022	5.90±3.56	1.01±0.76	0.002
	Mn–N(2)	2.2	2.232±0.015	6.49±4.28		
	Mn–C(1)	1.8	2.466±0.028	7.68±4.89		
	Mn–C(2)	2.1	2.725±0.036	8.27±5.35		
1.90-Mn_1@CN_x	Mn–N(1)	1.8	2.134±0.018	5.93±3.65	1.03±0.76	0.001
	Mn–N(2)	2.4	2.247±0.019	6.52±4.39		
	Mn–C(1)	2.0	2.491±0.029	7.70±5.12		
	Mn–C(2)	2.2	2.743±0.038	8.30±5.49		

[a]Coordination number. [b]Interatomic distance. [c]Debye-Waller factor. [d]Edge-energy shift. [e]The fit was carried out in R-space, and the R-factor is the measure of the goodness of fit. The lower the R-factor, the better the fit is.

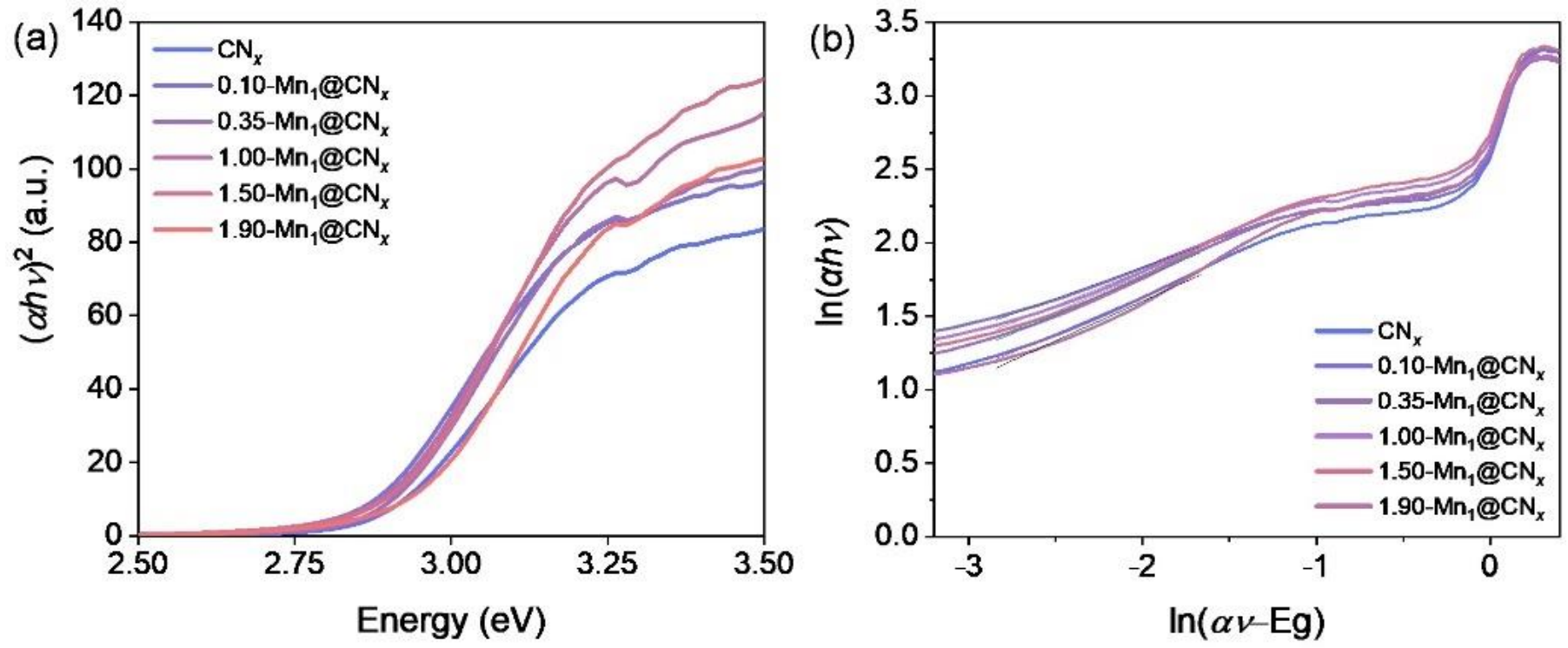

Figure S6. Tauc plot derived from DRS measurements on selected Mn_1@CN_x catalysts (a) and ln(αhν) against ln($h\nu - E_g$), where linear fitting was applied near the band-gap region (b).

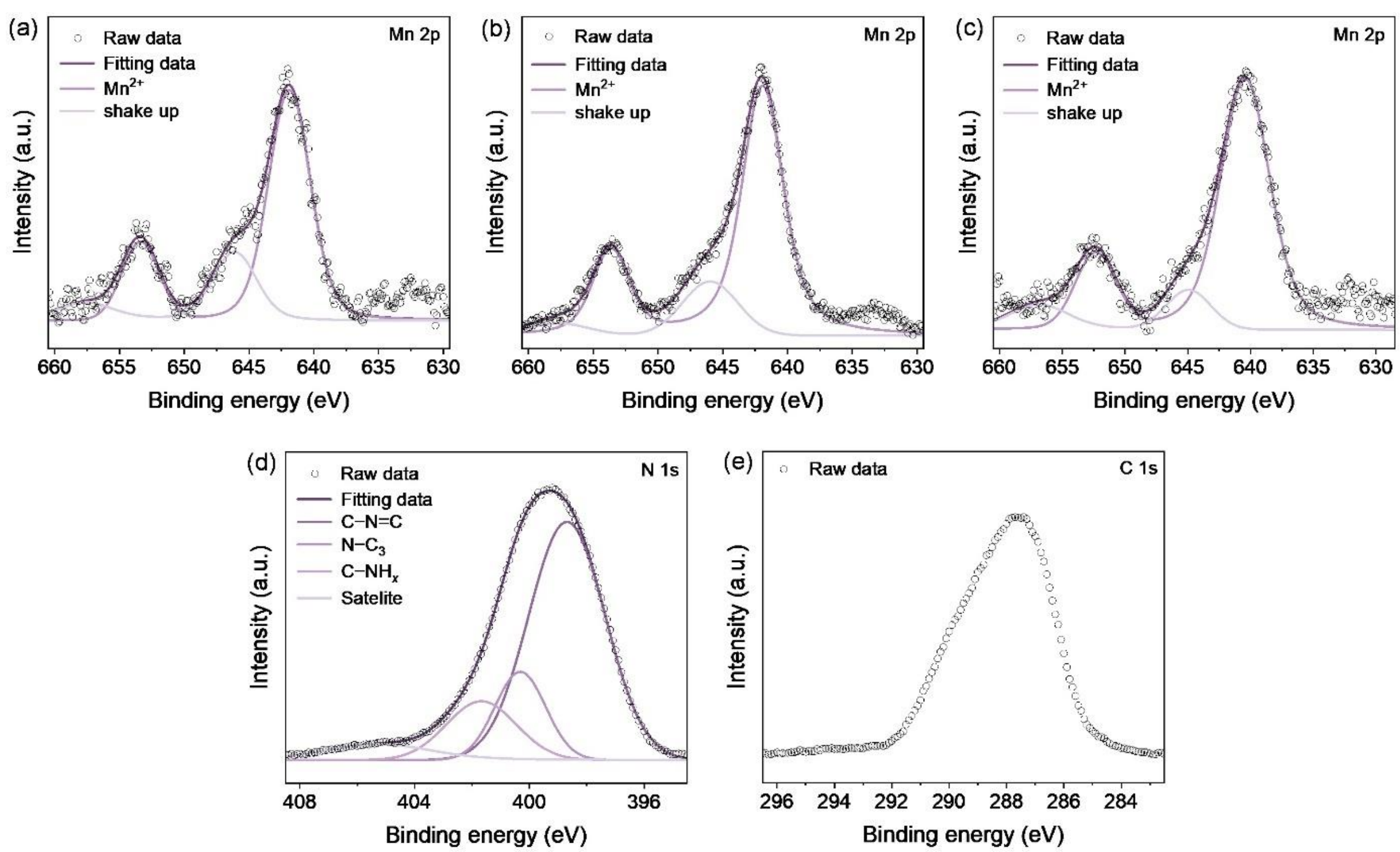

Figure S7. Mn 2p XPS measurements of 1.00-Mn_1@CN_x (a), 1.50-Mn_1@CN_x (b), and 1.90-Mn_1@CN_x (c). N 1s (d) and C 1s (e) XPS of 1.90-Mn_1@CN_x.

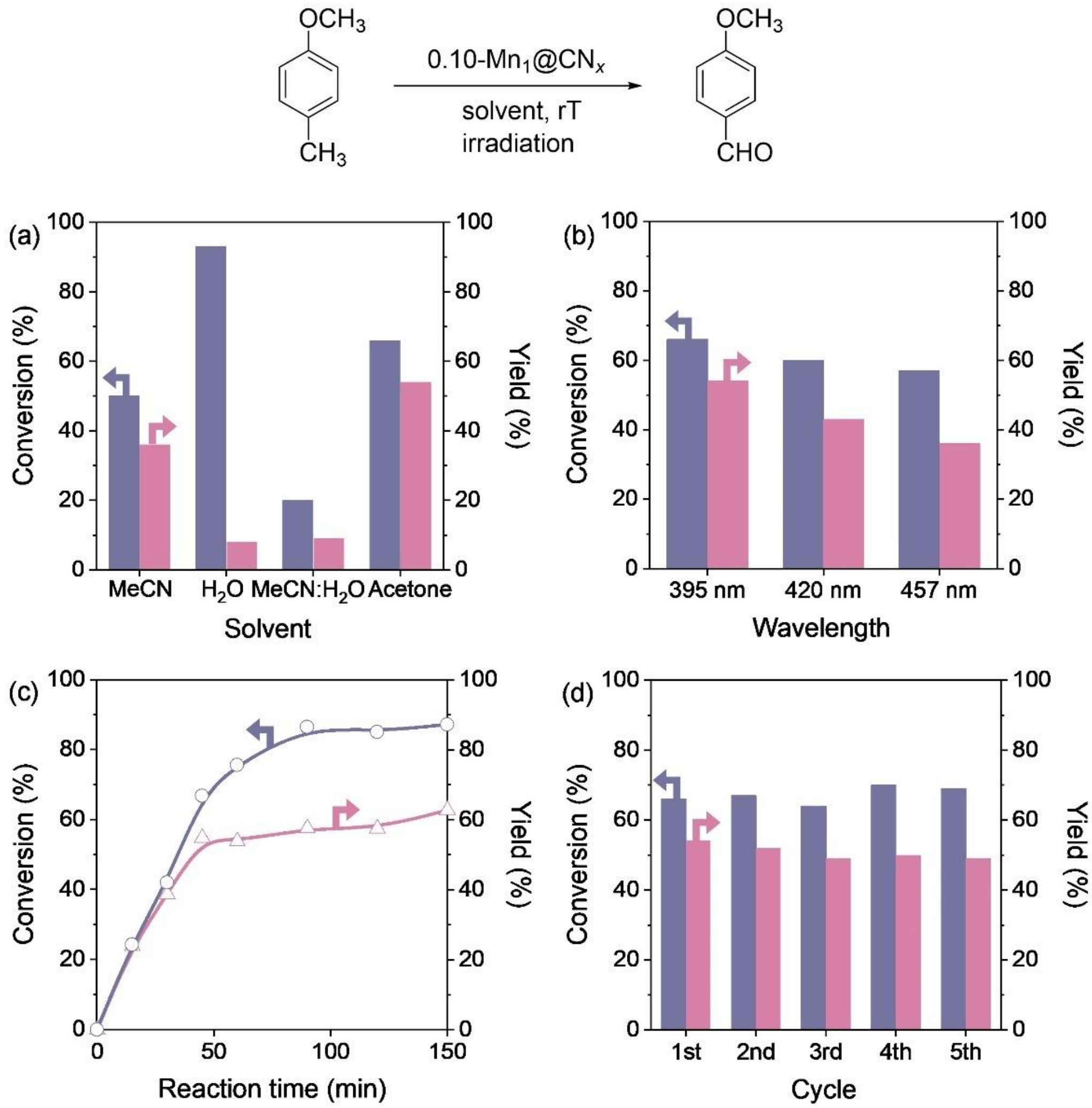

Figure S8. Effect of the solvent type (a), irradiation wavelength (b), reaction time (c), and catalyst recycling (d) for the light-driven oxidation of *p*-methoxytoluene to *p*-methoxybenzaldehyde. Unless otherwise specified, the reaction conditions were *p*-methoxytoluene (10 μL), solvent (1 mL, various solvents in (a) or acetone in (b-d)), 0.10-Mn_1@CN_x photocatalyst (5 mg), O_2 atmosphere, 25 °C, and irradiation at 395 nm (a,c,d) or different wavelengths (b).

Table S2. Comparison of catalytic activities for the selective photo-oxidation of *p*-methoxytoluene to *p*-methoxybenzaldehyde over various catalysts.

Entry	Catalyst	Reaction conditions	Rate ($\mu mol_{product}$ $mg_{catalyst}^{-1}$ h^{-1})	Ref.
1	0.10-Mn_1@CN_x	O_2 1 atm, Acetone, 25 ºC, 45 min	11.5	This work
2	MnO_2	Air, H_2SO_4, 25 ºC, 12 h	0.41	1
3	CeBTTD-A	O_2 1 atm, Acetonitrile, 25 ºC, 12 h	1.65	2
4	Co (II)/NHPI co-polymer	O_2 1 atm, neat, 80 ºC, 6 h	3.9	3
5	TiO_2	Air, neat, 25 ºC, 2.3 h	2.1	4
6	Pt/Bi_2WO_6	Air, Water, 25 ºC, 12 h	0.20	5
7	$CoMn_3Al_n$-Na	Air, Acetic acid, 105 ºC, 12 h	3.35	6
8	Bi_2MoO_6/$Cs_3Sb_2Br_9$	O_2 1 atm, neat, 25 ºC, 4 h	2.98	7
9	V_2O_5/MgO-Al_2O_3	Air, neat, 400 ºC, 4 h	0.76	8
10	CN_x	Air, water, 20 ºC, 1.5 h	0.35	9

Experimental procedures

Aerobic photo-oxidation in batch. The photocatalytic aerobic oxidation was performed using a PhotoCube™ reactor (ThalesNano Inc.) operating at 395 nm and 25 °C. In a typical procedure, the benzylic substrate (10 µL, 0.08 mmol), photocatalyst (5 mg, 0.10-Mn_1@CN_x), and solvent (1 mL, acetone) were added to a 4 mL quartz vial, which was sealed with a screw-cap septum. The sealed vial was purged with O_2 for 1 min by inserting an oxygen-filled balloon needle through the septum while venting through a second needle. After purging, both needles were removed, leaving the vial sealed with an oxygen-rich headspace. The vial was placed in the PhotoCube™ reactor, maintaining a constant distance (1 cm) between the vial and LED source, and irradiated while stirring (800 rpm) for 45 minutes. After completion, the reaction mixture was filtered through a PTFE syringe filter (0.45 µm) to remove catalyst particles, and with no additional work-up or extraction, reaction crude was concentrated in vacuo and submitted for analysis and purification. In order to obtain enough product for the purification, 8 vials were combined. The crude was thus purified via silica gel column chromatography (Hexane: Ethyl Acetate, 9:1 gradient) to give final products **2a-2k**.

Table S3. Control experiments for the photocatalytic oxidation of p-methoxytoluene.[a]

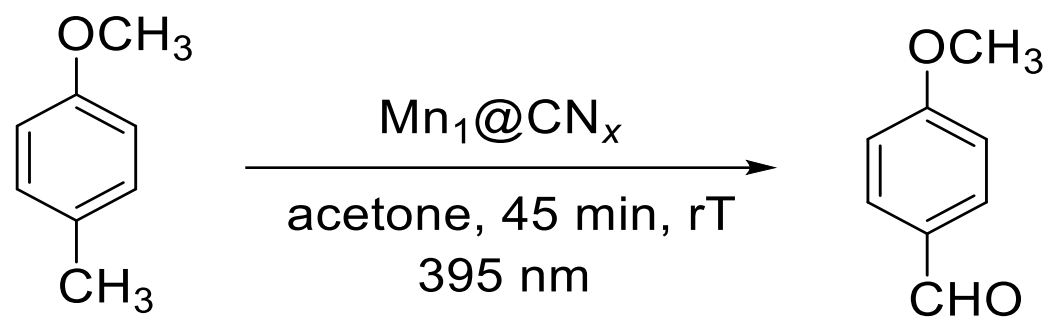

Entry	Deviation	Conversion (%)	Yield (%)
1	None	66	54
2	CN_x	35	16
3	In air	29	15
4	No light	0	0
5	No catalyst	0	0

[a]Reaction conditions: p-methoxytoluene (10 μL), acetone (1 mL), 25 °C, light irradiation (395 nm), catalyst (5 mg, 0.10-Mn_1@CN_x), O_2 atmosphere.

Aerobic photo-oxidation in flow.

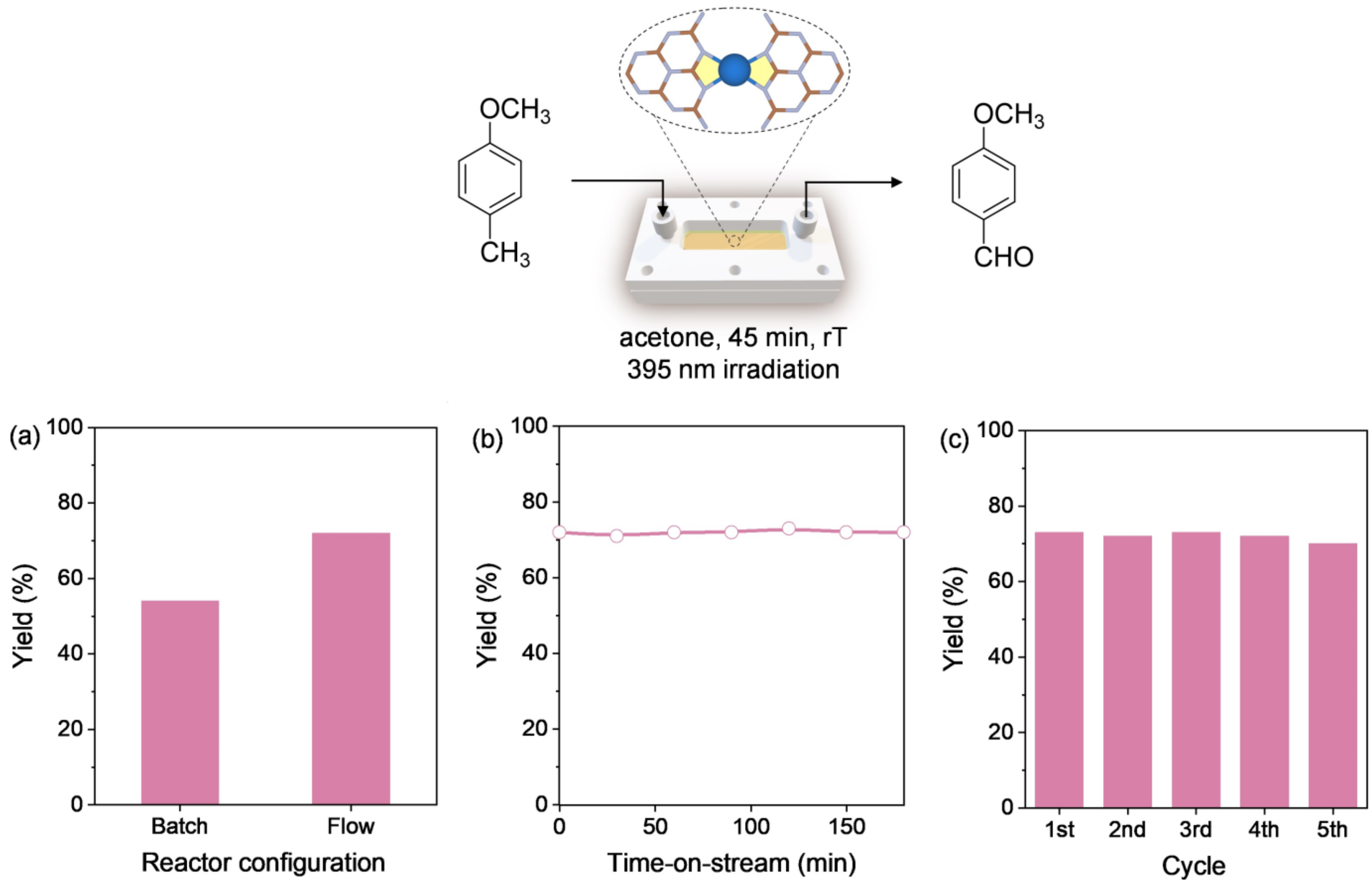

Figure S9. Comparison of p-methoxybenzaldehyde yield obtained in batch and continuous-flow configurations (a). Time-on-stream performance of the 0.10-Mn_1@CN_x thin film under continuous operation (b). Recyclability of the immobilized catalyst over five consecutive flow cycles (c). Reaction conditions: p-methoxytoluene (80 mmol L^{-1}) in acetone at 25 °C under O_2 and 395 nm irradiation, using 0.10-Mn_1@CN_x (5 mg). To perform the flow experiments, a stock solution of starting material was prepared using an 80 mmol L^{-1} concentration of substrate in acetone. Before each reaction run, the stock solution was flushed with an oxygen balloon for 5 minutes. The mixture was transferred to a 24 mL syringe and pumped with a syringe pump (PHD ULTRATM, Harvard apparatus) through the microchannel reactor (**Figure S12**) containing a 0.10-Mn_1@CN_x coated thin film (5 mg catalyst), with a flow rate of 19.1 µL/min at ambient conditions. To reach steady-state conditions, two reactor volumes (850 µL × 2) were flushed through the system and discarded. The reactor was placed under a violet LED lamp at 7 cm distance. After completion, the collected reaction mixture was concentrated in vacuo and submitted for analysis and purification. The crude was thus purified by silica gel column chromatography (Hexane: Ethyl Acetate, 9:1 gradient) to afford p-methoxybenzaldehyde.

Thin-film preparation for flow experiments. To prepare the catalyst-coated thin films, glass slides were cleaned by sonication in acetone and isopropanol for 15 min each, followed by drying under a stream of N_2. The cleaned slides were placed in a Petri dish and further dried in an oven at 130 °C, then removed immediately prior to the drop-casting process. A solution containing the catalyst and the binder (catalyst:binder 75:25 ratio) was then prepared:

N-Methyl-2-pyrrolidone (NMP, 0.5 mL; Sigma-Aldrich, 99%) was added to the catalyst (0.10-Mn_1@CN_x, 37.5 mg), and the mixture was sonicated for 1 h. Poly(vinylidene fluoride) (PVDF, 12.5 mg; Thermo Fisher Scientific) was then added to the dispersion, which was stirred at 60 °C for 16 h. The resulting solution was drop-cast onto the pre-cleaned glass slide. After drop-casting, the slide was dried in an oven for 20 min and subsequently used for photocatalytic experiments (**Figure S10**).

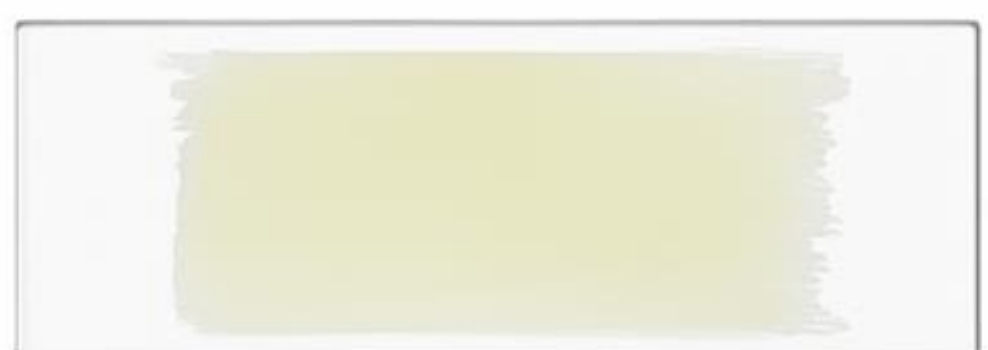

Figure S10. Visual representation of the catalytic film after drop-casting and drying the catalyst (0.10-Mn_1@CN_x).

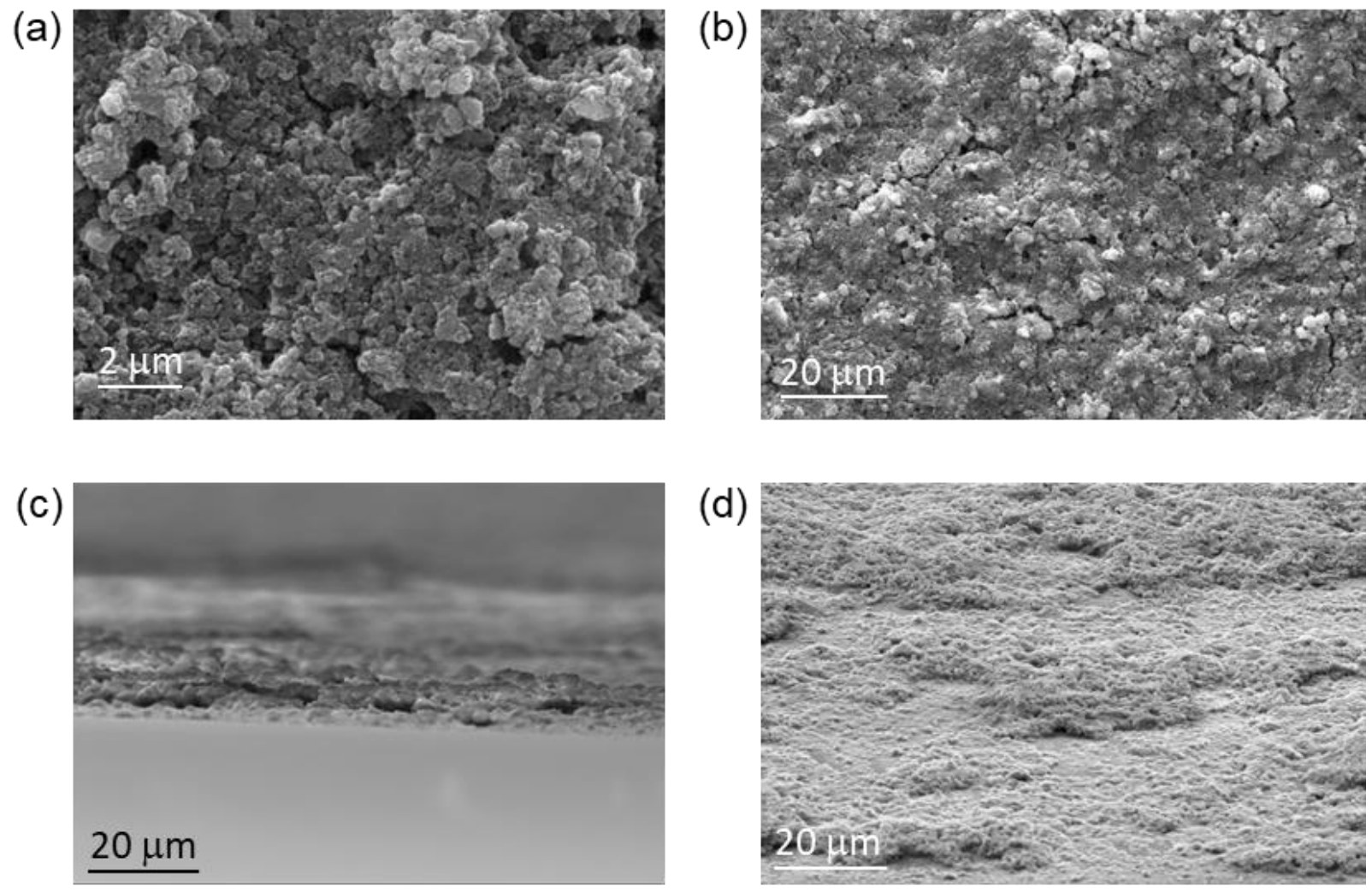

Figure S11. SEM images of the 0.10-Mn_1@CN_x thin film prepared by drop-casting onto a glass slide. Top-view micrographs showing uniform surface morphology of the catalyst layer (a,b). Cross-sectional view of the coated slide, displaying film thickness, uniformity, and internal contact with the glass (c,d).

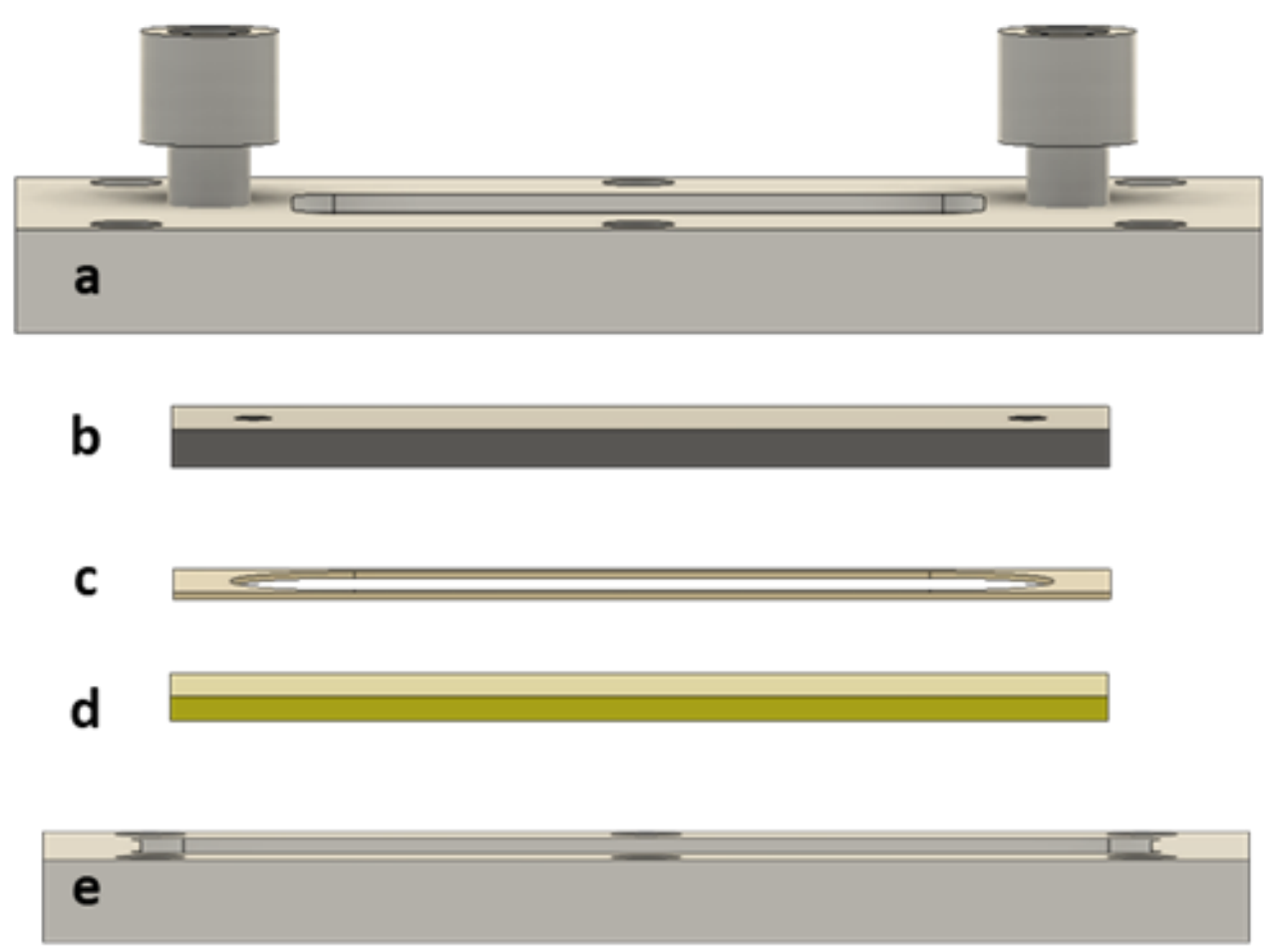

Figure S12. 3D-cut model of the microchannel reactor: stainless steel upper (**a**) and lower (**e**) reactor support, glass film (**b**), hollow Teflon channel (**c**), and catalytic film (**d**).

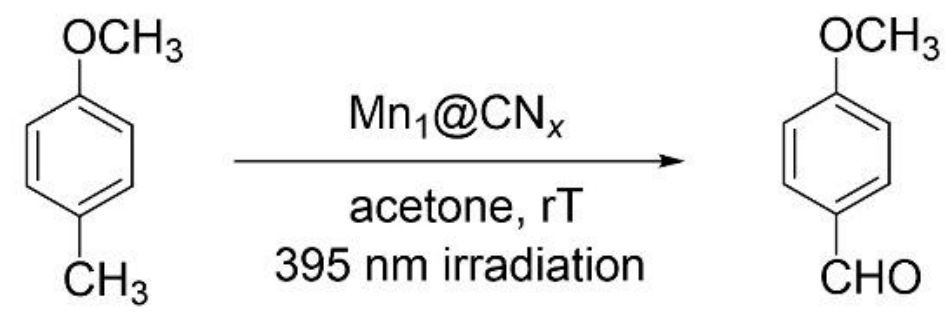

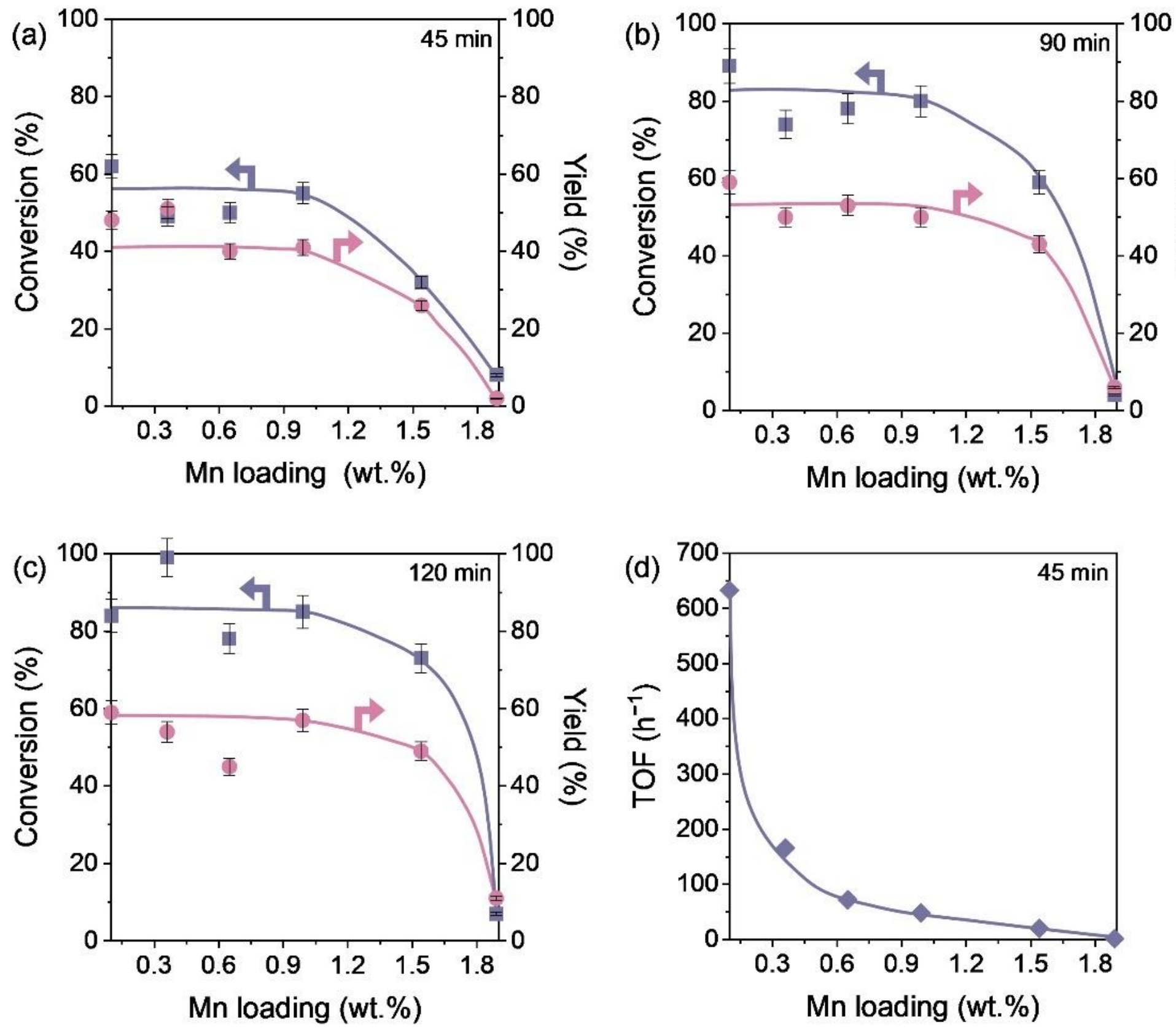

Figure S13. Catalytic performance of $Mn_1@CN_x$ samples as a function of Mn loading at 45 min (a), 90 min (b), and 120 min (c) of reaction time. TOFs of $Mn_1@CN_x$ samples after 45 min of reaction time (d). Reaction conditions: *p*-methoxytoluene (10 μL), acetone (1 mL), $Mn_1@CN_x$ catalyst (5 mg), O_2 atmosphere, 25 °C, and irradiation at 395 nm. This figure corresponds to the same catalytic data presented in **Figure 7** of the main manuscript, but including the estimated analytical uncertainty associated with chromatographic product quantification (±5%).

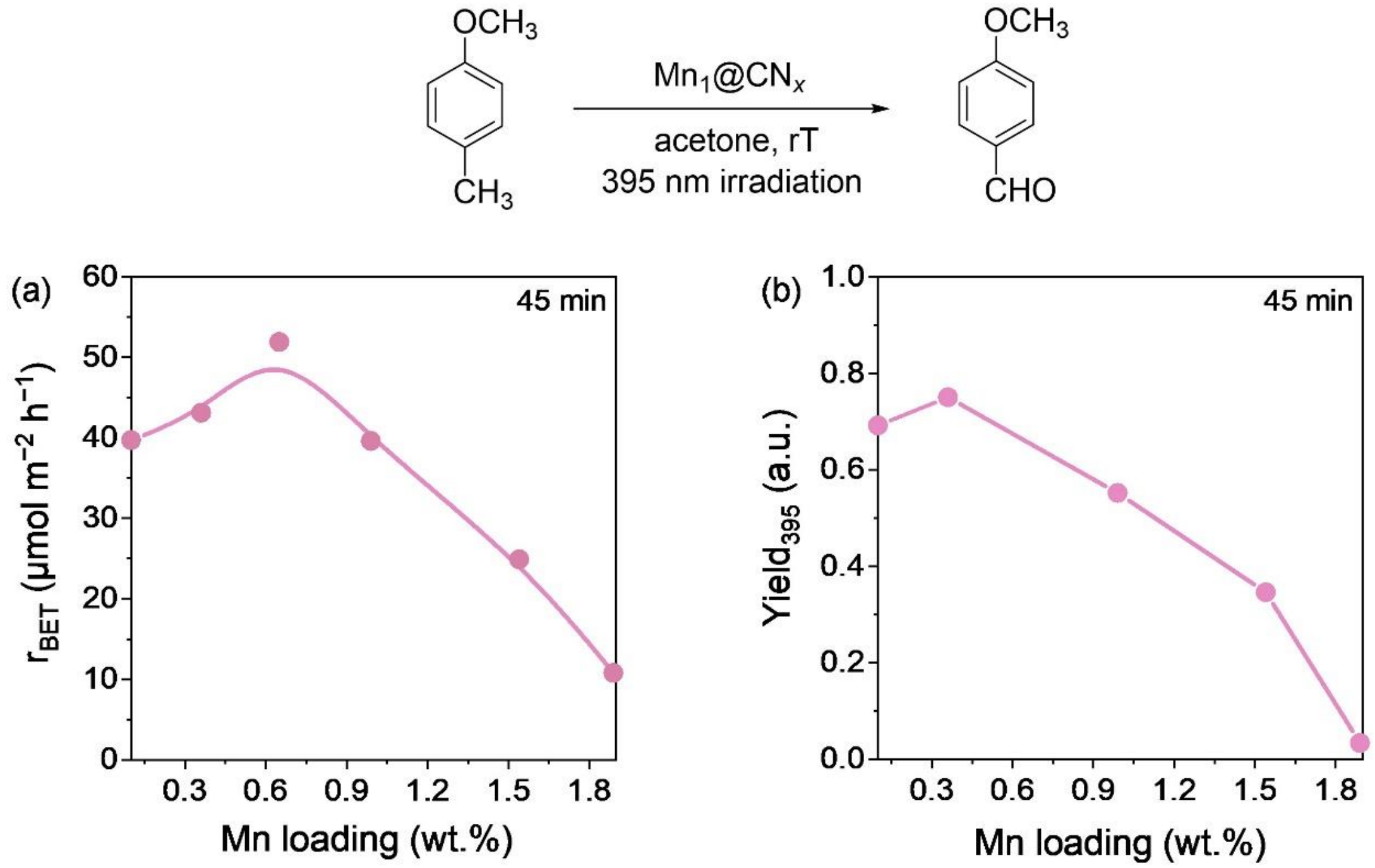

Figure S14. BET-normalized reaction rate (r_{BET}) (a) and aldehyde yield normalized by the optical absorption at 395 nm ($Yield_{395}$) (b) as a function of Mn loading for the $Mn_1@CN_x$ series after 45 min of reaction time. The same volcano-type dependence on Mn loading observed for the unnormalized catalytic data is retained after normalization, indicating that the decrease in activity at high Mn loadings is not primarily governed by textural or optical effects. Reaction conditions: *p*-methoxytoluene (10 µL), acetone (1 mL), $Mn_1@CN_x$ catalyst (5 mg), O_2 atmosphere, 25 °C, and irradiation at 395 nm.

XYZ coordinates of DFT-optimized structures

- **Mn@CN$_x$ low-loading**

C	3.917233	2.617349	10.140613
C	6.062088	3.869581	10.131543
C	6.071819	1.563209	10.125671
C	6.060993	6.174850	10.125710
C	3.943509	5.081379	10.199425
C	1.986910	3.858240	10.154594
C	12.989566	5.033745	9.959999
C	10.915175	3.845177	9.996146
C	10.835272	6.138570	10.003944
C	10.846342	1.532548	9.986696
C	12.997967	2.593269	9.963137
C	14.925865	3.804233	9.908948
C	3.901178	9.514512	10.142382
C	6.003017	10.779083	10.144642
C	6.051487	8.448872	10.125226
C	6.043789	13.092966	10.127622
C	3.916395	11.994295	10.200636
C	1.965899	10.757587	10.156713
C	12.973943	11.934899	9.962144
C	10.915330	10.740402	9.999930
C	10.826116	13.059516	10.016446
C	10.871007	8.393389	10.036866
C	12.986110	9.495444	9.977565
C	14.951944	10.733402	9.961234
C	3.886360	16.416098	10.145249

C	6.031525	17.680416	10.134057
C	6.043598	15.359717	10.128147
C	6.029470	19.990103	10.130384
C	3.913547	18.884913	10.198913
C	1.955101	17.657381	10.159057
C	12.957923	18.833085	9.964739
C	10.883397	17.649386	10.001842
C	10.803712	19.942319	10.008106
C	10.816277	15.334928	9.992149
C	12.967488	16.392490	9.967809
C	14.894899	17.602918	9.913361
C	8.917388	2.607743	7.133065
C	10.992668	3.863058	7.099728
C	11.079427	1.544801	7.109205
C	11.069910	6.180672	7.112156
C	8.914933	5.127134	7.139981
C	7.025350	3.880040	7.139176
C	2.379583	5.096409	6.935338
C	0.287922	3.859693	7.008371
C	0.252094	6.181726	7.003300
C	0.257988	1.563508	6.991540
C	2.405352	2.618030	6.991823
C	4.314243	3.851426	6.961060
C	8.898832	9.506594	7.129834
C	10.978971	10.762679	7.101554
C	11.064225	8.443644	7.111633
C	11.054651	13.080590	7.114580

C	8.893016	12.025042	7.131048
C	6.923320	10.767883	7.121697
C	2.350685	11.982524	6.936514
C	0.270915	10.757748	7.010139
C	0.236144	13.081372	7.005939
C	0.242873	8.462738	6.993769
C	2.390199	9.516596	6.993863
C	4.310201	10.757439	6.965630
C	8.885726	16.406063	7.135750
C	10.961204	17.661457	7.103387
C	11.048363	15.343698	7.113477
C	11.039358	19.979386	7.117013
C	8.883873	18.925079	7.143394
C	6.994489	17.678606	7.143727
C	2.334743	18.882537	6.940342
C	0.256000	17.657019	7.012360
C	0.221654	19.979829	7.008157
C	0.227666	15.362117	6.996298
C	2.374080	16.415584	6.996410
C	4.282270	17.651104	6.965765
N	4.626704	3.863096	10.127985
N	4.721096	1.563368	10.142649
N	6.744115	2.734006	10.138640
N	6.741767	5.018794	10.131272
N	4.711345	6.168130	10.146006
N	2.630078	2.652788	10.206921
N	2.600842	5.052652	10.269759

N	0.676344	3.892728	9.993269
N	6.773253	0.437633	10.127363
N	12.280814	3.843152	9.971833
N	12.224641	6.142208	9.990652
N	10.171158	4.956331	10.047849
N	10.212395	2.703372	9.978084
N	12.237643	1.532497	9.988941
N	14.298105	5.021081	9.846805
N	14.333726	2.619415	9.911680
N	10.166803	0.364216	10.048861
N	4.611119	10.764815	10.131411
N	4.706398	8.462767	10.145256
N	6.728939	9.640637	10.144922
N	6.723295	11.902470	10.145411
N	4.695690	13.082036	10.147447
N	2.616241	9.550598	10.207589
N	2.587051	11.953332	10.271812
N	0.662449	10.791156	10.008425
N	6.756179	7.328708	10.116851
N	12.263808	10.741999	9.974341
N	12.208861	13.040620	9.993750
N	10.161816	11.845357	10.053513
N	10.201375	9.597699	9.993782
N	12.221406	8.430344	9.993117
N	14.280490	11.925063	9.851187
N	14.292147	9.493064	9.979670
N	10.151572	7.264168	10.054469

N	4.596542	17.661436	10.132010
N	4.692511	15.354070	10.150274
N	6.712483	16.528417	10.133309
N	6.709415	18.820698	10.136761
N	4.679362	19.982385	10.150356
N	2.600096	16.449793	10.210870
N	2.570518	18.850972	10.274319
N	0.645688	17.690710	9.998037
N	6.762945	14.206341	10.127200
N	12.250824	17.640181	9.976237
N	12.196358	19.939333	9.994260
N	10.141005	18.753309	10.052438
N	10.180866	16.498997	9.981941
N	12.204769	15.333308	9.995212
N	14.267981	18.816856	9.850634
N	14.302903	16.417528	9.916315
N	10.135310	14.161897	10.053697
N	9.625845	3.874572	7.114508
N	9.684434	1.543626	7.115219
N	11.701080	2.731970	7.113004
N	11.699798	4.999356	7.116385
N	9.675042	6.188774	7.116316
N	7.585632	2.648960	7.195425
N	7.580159	5.094168	7.197006
N	5.643611	3.860333	6.999859
N	11.802982	0.419722	7.117122
N	1.696796	3.863565	6.990882

N	1.604027	6.177883	6.972919
N	15.211012	4.952866	7.041699
N	15.171288	2.696599	6.968313
N	1.606263	1.564584	6.987234
N	3.717017	5.052701	6.863775
N	3.692194	2.648870	6.924626
N	15.168726	0.385927	6.966412
N	9.611201	10.773483	7.117294
N	9.668468	8.442438	7.117780
N	11.685992	9.631006	7.115525
N	11.684564	11.897836	7.118796
N	9.659112	13.087878	7.118531
N	7.570753	9.544400	7.199782
N	7.564368	11.993234	7.199958
N	5.625182	10.760404	6.999843
N	11.787506	7.319278	7.119447
N	1.679293	10.758449	6.990280
N	1.588001	13.076168	6.975257
N	15.195903	11.852141	7.044050
N	15.155595	9.595787	6.971083
N	1.590593	8.463330	6.989746
N	3.697721	11.952334	6.867277
N	3.676505	9.549012	6.927565
N	15.152922	7.284503	6.968713
N	9.590301	17.664420	7.106498
N	9.653320	15.341385	7.120014
N	11.670420	16.529959	7.117716

N	11.668669	18.796890	7.121026
N	9.644085	19.986943	7.120996
N	7.554839	16.446951	7.200195
N	7.549337	18.892398	7.201660
N	5.613033	17.658347	7.005095
N	11.772202	14.217672	7.122016
N	1.663637	17.658041	6.992672
N	1.573046	19.976225	6.977293
N	15.180526	18.751470	7.046412
N	15.140420	16.494365	6.972882
N	1.576333	15.360795	6.991300
N	3.683573	18.850243	6.868834
N	3.661443	16.447404	6.929268
N	15.137935	14.184487	6.971047
Mn	8.438532	10.736868	9.787838
N	-0.381649	4.981889	7.067726
N	-0.421372	2.725621	6.994340
N	-0.396758	11.881164	7.070077
N	-0.437066	9.624810	6.997110
N	-0.439739	7.313526	6.994740
N	-0.412135	18.780493	7.072439
N	-0.452241	16.523388	6.998909
N	-0.454726	14.213510	6.997074

- **Mn@CN$_x$ high-loading**

C	3.863767	2.631574	10.371946
C	5.880249	3.843369	10.969763

C	5.915514	1.543810	10.658321
C	5.662667	6.146977	11.211071
C	3.704692	4.896710	11.225083
C	1.868692	3.737007	10.540102
C	13.165844	5.345860	9.852368
C	11.116892	4.118777	9.387770
C	11.122730	6.438136	9.451590
C	11.185853	1.807995	9.622237
C	13.000639	2.996246	10.436772
C	14.970183	4.107180	10.436735
C	3.623436	9.624499	10.419190
C	5.752009	10.757722	10.719764
C	5.599425	8.457570	10.828064
C	5.715803	13.076643	10.597235
C	3.671855	12.048581	10.719734
C	1.765503	10.935441	10.162296
C	12.908990	12.185307	9.728078
C	10.877728	10.974358	10.217334
C	10.968071	13.318000	10.170442
C	11.008914	8.675482	9.964742
C	13.042740	9.820994	10.191169
C	14.900175	11.036640	9.638065
C	3.867813	16.505842	9.943349
C	5.928980	17.653115	10.505249
C	5.877219	15.374038	10.170349
C	5.839965	19.948045	10.771630
C	3.777158	18.807126	10.669393

C	1.864551	17.572276	10.299305
C	13.189796	18.974857	9.939324
C	11.018880	17.887683	9.735616
C	11.262703	20.168436	9.438127
C	10.888817	15.648212	10.299058
C	12.861007	16.736092	10.804538
C	14.960757	17.652931	10.614799
C	8.724586	2.360224	6.680113
C	10.731910	3.584636	6.029352
C	10.746252	1.272259	6.169049
C	10.668929	5.914232	5.942931
C	8.638901	4.778770	6.364530
C	6.863446	3.580830	7.094683
C	2.421438	4.984128	7.481100
C	0.320138	3.792175	7.170561
C	0.368508	6.108291	7.244809
C	0.388356	1.477828	7.090749
C	2.446409	2.606804	6.966820
C	4.342274	3.777618	7.390405
C	8.792587	9.379174	6.546611
C	11.003227	10.471470	6.566295
C	10.771663	8.211141	6.103632
C	10.982637	12.747343	7.060645
C	9.049117	11.583629	7.449343
C	7.041594	10.499374	7.503423
C	2.748744	11.770745	6.798689
C	0.562563	10.660792	6.695139

C	0.816469	12.978621	6.605065
C	0.462083	8.356671	6.886331
C	2.604963	9.339277	6.924452
C	4.551679	10.441010	7.246619
C	8.885025	16.159126	6.882458
C	10.954888	17.383482	6.510322
C	10.946094	15.104968	6.841949
C	10.806922	19.674551	6.132670
C	8.786619	18.503935	6.310312
C	6.940243	17.333856	6.921973
C	2.511257	18.879234	7.287986
C	0.449172	17.605739	7.055774
C	0.402308	19.904089	7.326615
C	0.660111	15.365532	6.593129
C	2.578647	16.609255	6.418021
C	4.457099	17.750390	6.979355
N	4.481842	3.793164	10.836540
N	4.629908	1.553963	10.181791
N	6.579147	2.710922	10.922051
N	6.455174	5.030084	11.154448
N	4.335061	6.028939	11.547686
N	2.566720	2.642002	10.112455
N	2.378904	4.794474	11.246521
N	0.611699	3.787196	10.149907
N	6.577808	0.396346	10.813795
N	12.444381	4.150351	9.848975
N	12.483155	6.456722	9.575836

N	10.473112	5.269920	9.179047
N	10.536743	2.935589	9.195295
N	12.289774	1.872478	10.447965
N	14.470879	5.315753	10.104760
N	14.235116	3.059689	10.934052
N	10.656959	0.632708	9.275120
N	4.345996	10.819468	10.611251
N	4.260306	8.472555	10.507475
N	6.356727	9.598978	10.862280
N	6.430742	11.920800	10.653798
N	4.386375	13.170175	10.831504
N	2.305845	9.714891	10.119499
N	2.344069	12.061224	10.668013
N	0.613064	11.247883	9.511554
N	6.266789	7.319590	11.045243
N	12.289798	11.010214	10.154630
N	12.263939	13.333320	9.766916
N	10.238225	12.156238	10.330754
N	10.256545	9.818868	10.076687
N	12.351470	8.686711	10.269285
N	14.136210	12.112575	9.189368
N	14.370549	9.905113	10.155801
N	10.393170	7.562231	9.563161
N	4.521892	17.636081	10.455392
N	4.598195	15.405810	9.705035
N	6.591196	16.486346	10.407997
N	6.570624	18.795967	10.667908

N	4.471079	19.929486	10.890217
N	2.570522	16.526399	9.713831
N	2.445670	18.755249	10.654347
N	0.605019	17.261907	10.508513
N	6.486081	14.159511	10.330480
N	12.365216	17.850386	10.116186
N	12.624800	20.122592	9.576899
N	10.477140	19.038807	9.359863
N	10.308887	16.739286	9.755709
N	12.061483	15.683668	11.005592
N	14.501565	18.852773	10.169115
N	14.125517	16.720043	11.208892
N	10.234060	14.440579	10.222740
N	9.360790	3.578591	6.375167
N	9.431525	1.252001	6.547796
N	11.394934	2.442329	5.928506
N	11.346372	4.747886	5.823993
N	9.302484	5.884466	6.034901
N	7.447012	2.383885	7.085962
N	7.336054	4.762548	6.653798
N	5.661834	3.692374	7.772774
N	11.476233	0.152307	6.076500
N	1.739598	3.799015	7.178785
N	1.698358	6.093393	7.599495
N	15.261025	4.920916	7.139076
N	15.272512	2.601808	7.142067
N	1.739683	1.498531	6.789791

N	3.741627	4.946178	7.682374
N	3.788251	2.639755	6.936062
N	15.325268	0.298224	7.214683
N	9.596515	10.508969	6.780364
N	9.401173	8.289015	6.126464
N	11.561749	9.324797	6.240623
N	11.687750	11.632141	6.708188
N	9.745133	12.690015	7.615259
N	7.471302	9.475063	6.745617
N	7.820098	11.518517	7.975891
N	5.752406	10.525166	7.902842
N	11.388687	7.042456	5.945434
N	1.975739	10.594870	6.791999
N	2.155712	12.945890	6.699629
N	0.000395	11.877102	6.564211
N	15.428739	9.532881	6.738059
N	1.831631	8.269995	6.830917
N	4.075607	11.661944	6.946379
N	3.920729	9.266299	7.148731
N	15.314805	7.228799	6.995543
N	9.541156	17.357529	6.603332
N	9.596085	15.046579	6.892632
N	11.653489	16.249136	6.673869
N	11.558292	18.546106	6.281852
N	9.447534	19.598041	5.963703
N	7.569095	16.166471	7.126456
N	7.454742	18.451471	6.350677

N	5.700868	17.499251	7.450415
N	11.658845	13.917980	6.896345
N	1.851983	17.695286	6.925033
N	1.758526	19.943337	7.532025
N	15.338046	18.682342	7.234208
N	15.469088	16.370480	6.940632
N	1.938937	15.486222	6.132786
N	3.837416	18.860797	7.424487
N	3.910606	16.737841	6.265062
N	0.084831	14.125596	6.647527
Mn	8.295819	13.095180	9.711808
Mn	13.770965	13.175892	7.163564
N	15.593056	11.848079	6.538184
N	15.677492	14.096573	6.621500
N	-0.331636	4.949939	7.165103
N	-0.320149	2.630831	7.168094
N	-0.163922	9.561904	6.764086
N	-0.277855	7.257822	7.021570
N	-0.254615	18.711365	7.260235
N	-0.123572	16.399503	6.966659

- CN_x

N	-0.00013	-0.00026	0.06635
C	-0.00027	0.00062	1.39485
H	0.87159	0.00129	-0.43601
H	-0.87181	-0.00268	-0.43607
N	-1.19727	-0.00171	1.99883

N	1.19645	0.00358	1.99922
C	-1.20765	-0.00147	3.31691
C	1.20634	0.00460	3.31738
N	-2.34441	-0.00407	3.98456
N	-0.00074	0.00181	4.01418
N	2.34291	0.00814	3.98535
C	-2.26930	-0.00422	5.32311
C	-0.00097	0.00239	5.40775
C	2.26735	0.00925	5.32390
N	-1.14775	-0.00104	6.05788
N	-3.41973	-0.00741	5.98766
N	1.14556	0.00627	6.05825
N	3.41758	0.01322	5.98876
H	-4.29136	-0.00979	5.48519
H	-3.41855	-0.00731	6.99356
H	4.28930	0.01571	5.48644
H	3.41624	0.01455	6.99465

- $^{3}CN_{x}$

N	-0.07971	0.18397	0.13048
C	0.04090	-0.03343	1.44282
H	0.73820	0.35456	-0.42979
H	-0.98609	0.14128	-0.30386
N	-1.08234	-0.26893	2.12592
N	1.29131	-0.02767	1.94757
C	-0.94660	-0.56468	3.46771
C	1.41056	-0.04172	3.27317

N	-2.07222	-0.27332	4.21191
N	0.30146	-0.19527	4.06057
N	2.59942	0.10362	3.82903
C	-1.89174	-0.03910	5.51410
C	0.39251	-0.04094	5.41765
C	2.64149	0.12488	5.17136
N	-0.70983	-0.02830	6.16335
N	-2.98491	0.17312	6.25192
N	1.57494	0.10520	5.98745
N	3.84018	0.19926	5.73965
H	-3.89543	0.12308	5.82718
H	-2.90030	0.33913	7.24056
H	4.66650	0.24083	5.16699
H	3.92136	0.24349	6.74155

- **CN_x^+**

N	0.00025	0.00210	0.06620
C	-0.00082	-0.00359	1.38215
H	0.87315	0.03133	-0.43773
H	-0.87176	-0.02433	-0.43941
N	-1.19667	-0.04368	2.00537
N	1.19393	0.03179	2.00773
C	-1.20024	-0.05218	3.32216
C	1.19558	0.01935	3.32450
N	-2.33971	-0.08923	3.98210
N	-0.00281	-0.02347	4.01582
N	2.33403	0.04718	3.98668

C -2.28373 -0.09546 5.32961

C -0.00370 -0.03852 5.39916

C 2.27626 0.02517 5.33394

N -1.14571 -0.07457 6.05473

N -3.42347 -0.12507 5.98713

N 1.13740 -0.01583 6.05691

N 3.41502 0.04689 5.99347

H -4.29752 -0.14160 5.48467

H -3.42503 -0.13146 6.99512

H 4.28958 0.07772 5.49258

H 3.41535 0.03035 7.00134

- **Ar–CH_3 (p-methoxytoluene)**

C 0.02387 -0.00000 0.00884

C 0.02584 0.00000 1.40317

C 1.20609 0.00000 2.12610

C 2.43331 -0.00000 1.46611

C 2.45517 -0.00000 0.07380

C 1.25823 -0.00000 -0.63196

C -1.25523 0.00000 -0.77255

H 1.19348 0.00000 3.21052

O 3.53701 -0.00000 2.24705

H 3.39150 -0.00000 -0.46887

H 1.29448 -0.00000 -1.71753

H -0.91980 0.00000 1.93666

H -2.12162 -0.00001 -0.10873

H -1.32732 0.87955 -1.41917

H	-1.32732	-0.87954	-1.41918
C	4.79596	-0.00000	1.60508
H	5.54201	-0.00000	2.39885
H	4.93079	-0.89306	0.98655
H	4.93079	0.89305	0.98655

- **^{3}Ar–CH$_3$**

C	0.02702	-0.00000	0.01129
C	0.02337	-0.00000	1.38290
C	1.21394	0.00000	2.11546
C	2.50338	0.00000	1.36975
C	2.53673	0.00000	-0.00086
C	1.33369	-0.00000	-0.72287
C	-1.20306	-0.00000	-0.80516
H	1.24364	0.00000	3.19626
O	3.55411	0.00000	2.18837
H	3.48249	0.00000	-0.52893
H	1.32625	-0.00000	-1.80476
H	-0.92715	-0.00000	1.90830
H	-2.10695	-0.00000	-0.19440
H	-1.22224	0.87429	-1.47113
H	-1.22224	-0.87429	-1.47113
C	4.85114	0.00000	1.61114
H	5.55362	0.00000	2.44237
H	5.00192	-0.89376	0.99994
H	5.00192	0.89376	0.99994

- **Ar–CH_2•**

C	0.01716	-0.09661	-0.00886
C	-0.00044	-0.00007	1.40318
C	1.25977	0.11707	2.04864
C	2.43174	0.13504	1.33680
C	2.41687	0.03767	-0.06217
C	1.19457	-0.07866	-0.72919
C	-1.19680	-0.01864	2.12841
O	3.61782	0.06512	-0.67135
C	3.64790	-0.02905	-2.08306
H	3.38775	0.22508	1.84096
H	1.15887	-0.15521	-1.80824
H	-0.92589	-0.18735	-0.53810
H	1.28863	0.19352	3.13075
H	-1.19267	0.05665	3.20935
H	-2.15135	-0.10804	1.62340
H	4.69866	0.01223	-2.36628
H	3.21833	-0.97420	-2.42895
H	3.11573	0.80546	-2.54996

- **Ar–CH_2 –O–O•**

C	0.01805	0.00000	-0.00719
C	0.00822	0.00000	1.37854
C	1.23161	0.00000	2.05083
C	2.42410	0.00000	1.35840
C	2.42566	0.00000	-0.03842
C	1.21433	0.00000	-0.71952

C	-1.24477	0.00000	2.19654
O	3.63670	0.00000	-0.63345
C	3.67405	-0.00000	-2.04738
H	3.37166	0.00000	1.88528
H	1.18300	0.00000	-1.80107
H	-0.91219	0.00000	-0.56181
H	1.24808	0.00000	3.13642
H	4.72788	-0.00000	-2.32243
H	3.19427	-0.89322	-2.45982
H	3.19427	0.89322	-2.45982
O	-2.39735	0.00000	1.32385
H	-1.31612	-0.88881	2.82899
H	-1.31612	0.88881	2.82899
O	-3.50572	0.00000	1.99535

- **Ar–CH_2OOH**

C	0.00561	0.00000	-0.00065
C	0.00080	0.00000	1.39214
C	1.18243	-0.00000	2.11593
C	2.38811	-0.00000	1.41241
C	2.40960	-0.00000	0.03328
C	1.21334	-0.00000	-0.68781
C	1.22903	-0.00000	3.61509
O	-0.08552	-0.00000	4.11535
O	0.06377	-0.00000	5.54033
O	1.32715	-0.00000	-2.03351
C	0.13416	-0.00000	-2.79176

H	3.34916	-0.00000	-0.50803
H	-0.93719	0.00000	-0.53177
H	-0.95175	0.00000	1.90797
H	3.32688	-0.00000	1.95815
H	0.43931	-0.00000	-3.83738
H	-0.46654	-0.89316	-2.59218
H	-0.46654	0.89316	-2.59218
H	1.76276	-0.88727	3.97996
H	1.76276	0.88727	3.97996
H	-0.86787	-0.00000	5.80358

- **Ar–CHO (p-methoxybenzaldehyde)**

C	0.02935	-0.00001	0.00563
C	0.02748	-0.00000	1.40311
C	1.20968	-0.00000	2.10842
C	2.42831	-0.00000	1.42202
C	2.44386	-0.00000	0.02253
C	1.24933	-0.00000	-0.66941
C	-1.24278	-0.00001	-0.71054
H	1.21988	-0.00000	3.19215
O	3.53050	-0.00000	2.18223
H	3.37905	-0.00000	-0.52089
H	1.25229	-0.00001	-1.75385
H	-0.91996	-0.00000	1.93353
C	4.79505	0.00000	1.53905
H	5.53562	0.00000	2.33708
H	4.92611	-0.89422	0.92329

H	4.92611	0.89422	0.92329
O	-1.37027	0.00000	-1.91713
H	-2.13571	0.00000	-0.05300

- **O_2**

O	0.00000	0.00000	0.03034
O	0.00000	0.00000	1.22286

- **$O_2\bullet^-$**

O	0.00000	0.00000	-0.03511
O	0.00000	0.00000	1.29024

- **$O_2H\bullet$**

O	-0.00205	0.00000	0.01912
O	0.01622	0.00000	1.32603
H	0.93475	0.00000	-0.26706

NMR characterizations of the final compounds

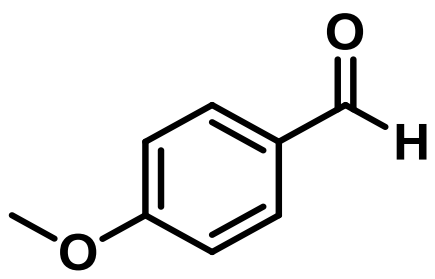

*4-Methoxybenzaldehyde (**2a**)*. Light yellow liquid, flash column chromatography (Hexane: Ethyl Acetate 9:1). ^{1}H NMR (400 MHz, $CDCl_3$) δ 9.89 (s, 1H), 7.87 (d, J = 8.8 Hz, 2H), 7.02 (d, J = 8.7 Hz, 2H), 3.90 (s, 3H); ^{13}C NMR (101 MHz, $CDCl_3$) δ 190.39, 164.2, 131.57, 129.55, 113.9, 55.17. Spectral data is in accordance with the literature.[10]

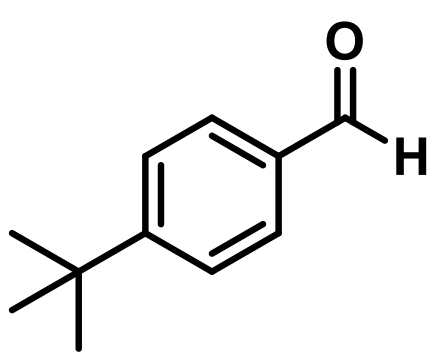

*4-(tert-Butyl)benzaldehyde (**2b**)*. Yellow oily liquid, flash column chromatography (Hexane: Ethyl Acetate 9:1). ^{1}H NMR (400 MHz, $CDCl_3$) δ 9.98 (s, 1H), 7.81 (d, J = 8.8 Hz, 2H), 7.53 (d, J = 8.8 Hz, 2H), 1.36 (s, 9H); ^{13}C NMR (101 MHz, $CDCl_3$) δ 192.39, 158.69, 134.24, 129.94, 126.20, 125.4, 35.40, 31.30. Spectral data is in accordance with the literature.[11]

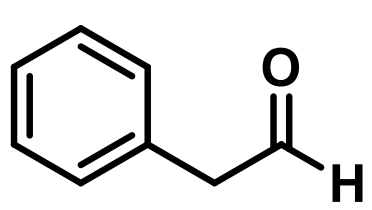

*2-Phenylacetaldehyde (**2c**)*. Colorless oil, flash column chromatography (Hexane: Ethyl Acetate 9:1). ^{1}H NMR (400 MHz, $CDCl_3$): δ 9.76 (t, J = 2.2, 1H), 7.38 (m, 2H), 7.31 (m, 1H), 7.23 (m, 2H), 3.69 (d, J = 2.2, 2H). ^{13}C NMR (101 MHz, $CDCl_3$) δ 199.4, 131.9, 129.6, 129.0, 127.4, 50.6. Spectral data is in accordance with the literature.[12]

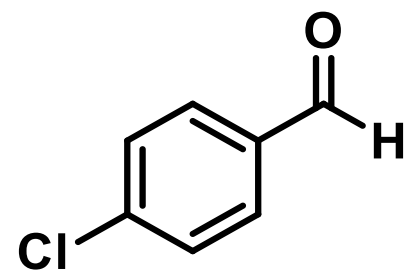

*4-Chlorobenzaldehyde (**2d**)*. Light yellow liquid, flash column chromatography (Hexane: Ethyl Acetate 9:1). ^{1}H NMR (400 MHz, $CDCl_3$) δ 9.97 (s, 1H), 7.82 (d, J = 8.4 Hz, 2H), 7.50 (d, J = 8.4 Hz, 2H); ^{13}C NMR (101 MHz, $CDCl_3$) δ 190.9, 140.9, 134.7, 130.9, 129.4. Spectral data is in accordance with the literature.[10]

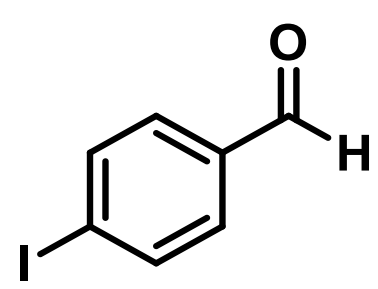

*4-Iodobenzaldehyde (**2e**)*. Pale yellow solid, flash column chromatography (Hexane: Ethyl Acetate 9:1). ^{1}H NMR (400 MHz, $CDCl_3$): δ = 9.96 (s, 1H), 7.92-7.90 (d, J = 8.37 Hz, 2H), 7.60-7.58 (d, J = 8.37 Hz, 2H); ^{13}C NMR (101 MHz, $CDCl_3$): δ = 191.51, 138.54, 135.70, 130.92, 102.94. Spectral data is in accordance with the literature.[13]

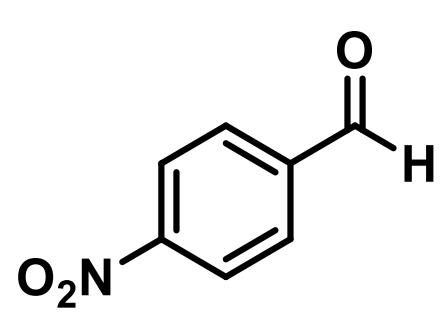

*4-Nitrobenzaldehyde (**2f**)*. Pale yellow solid, flash column chromatography (Hexane: Ethyl Acetate 9:1). ^{1}H NMR (400 MHz, $CDCl_3$) δ (ppm): 10.14 (s, 1H), 8.37 (d, J = 8.7 Hz, 2H), 8.06 (d, J = 8.7 Hz, 2H). ^{13}C NMR (101 MHz, $CDCl_3$): 190.47, 151.81, 140.11, 130.59, 124.40. Spectral data is in accordance with the literature.[14]

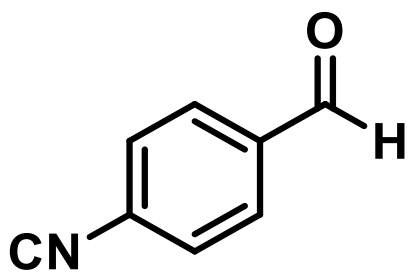

*4-Cyanobenzaldehyde (**2g**)*. White solid, flash column chromatography (Hexane: Ethyl Acetate 9:1). ^{1}H NMR (500 MHz, $CDCl_3$): δ 10.10 (s, 1H), 8.00 (d, J = 8.4 Hz, 2H), 7.85 (d, J = 8.2 Hz, 2H). ^{13}C NMR (101 MHz, $CDCl_3$): δ 190.73, 138.86, 133.05, 130.04, 117.86, 117.77. Spectral data is in accordance with the literature.[15]

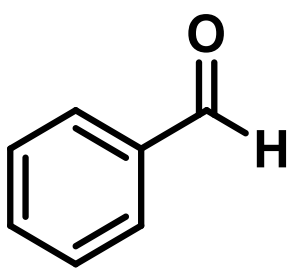

*Benzaldehyde (**2h**)*. Transparent liquid, flash column chromatography (Hexane: Ethyl Acetate 99:1). ^{1}H NMR (400 MHz, $CDCl_3$) δ 10.01 (s, 1H), 7.87 (d, J = 7.6 Hz, 2H), 7.62 (t, J = 7.4 Hz, 1H), 7.52 (t, J = 7.5 Hz, 2H); ^{13}C NMR (101 MHz, $CDCl_3$) δ 192.5, 136.4, 134.6, 129.8, 129.1. Spectral data is in accordance with the literature.[10]

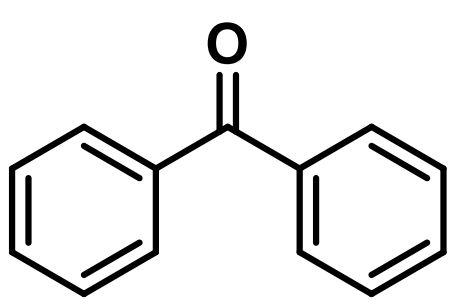

*Benzophenone (**2i**)*. White solid, flash column chromatography (Hexane: Ethyl Acetate 99:1). ^{1}H NMR (400 MHz, $CDCl_3$): 7.79 (d, J = 7.1 Hz, 4H), 7.59-7.44 (m, 6H). ^{13}C NMR (101 MHz, $CDCl_3$): 196.93, 137.64, 132.55, 130.17, 128.39. Spectral data is in accordance with the literature.[14]

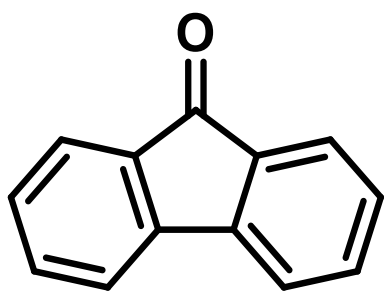

9H-Fluoren-9-one (***2j***). Pale yellow solid, flash column chromatography (Hexane: Ethyl Acetate 99:1). ^{1}H NMR (400 MHz, $CDCl_3$): δ 7.66 (d, J = 7.3 Hz, 2H), 7.55-7.46 (m, 4H), 7.29 (td, J = 7.3, 1.2 Hz, 2H); ^{13}C NMR (101 MHz, $CDCl_3$): δ 193.6, 144.1, 134.5, 133.9, 128.8, 123.9, 120.1. Spectral data is in accordance with the literature.[10]

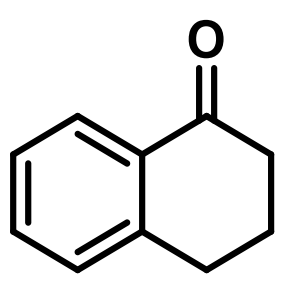

3,4-Dihydronaphthalen-1(2H)-one (***2k***). Yellow solid, flash column chromatography (Hexane: Ethyl Acetate 5:1). ^{1}H NMR (400 MHz, $CDCl_3$): δ 8.04 (d, J = 7.7 Hz, 1H), 7.46 (d, J = 7.5 Hz, 1H), 7.32 (t, J = 7.5 Hz, 1H), 7.25 (s, 1H), 2.98 (t, J = 6.0 Hz, 2H), 2.70 – 2.62 (m, 2H), 2.15 (q, 2H); ^{13}C NMR (101 MHz, $CDCl_3$) δ 198.5, 144.5, 133.4, 132.6, 128.8, 127.2, 126.7, 39.2, 29.7, 23.3. Spectral data is in accordance with the literature.[16]

*4-Methoxybenzaldehyde (**2a**)*

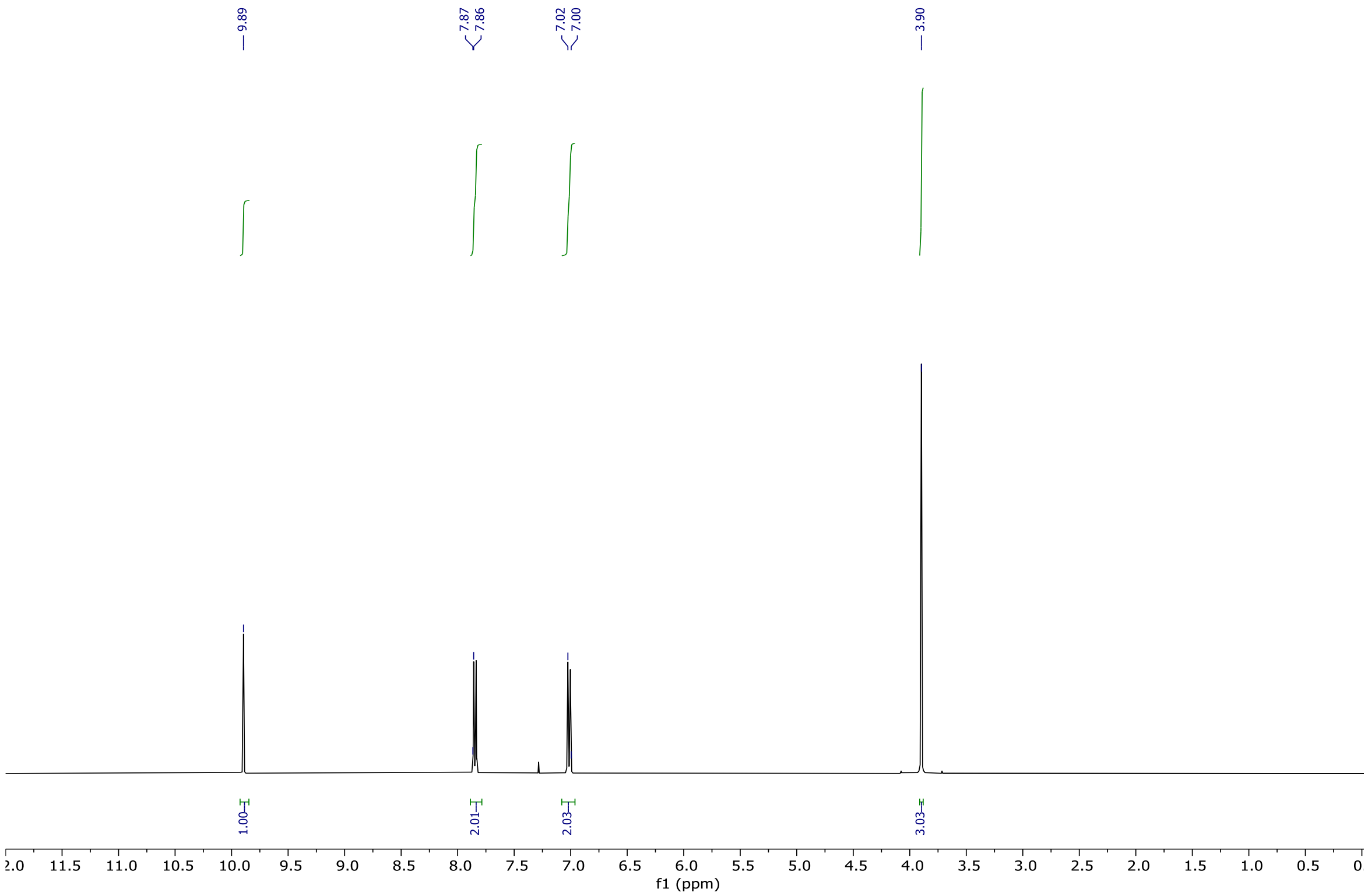

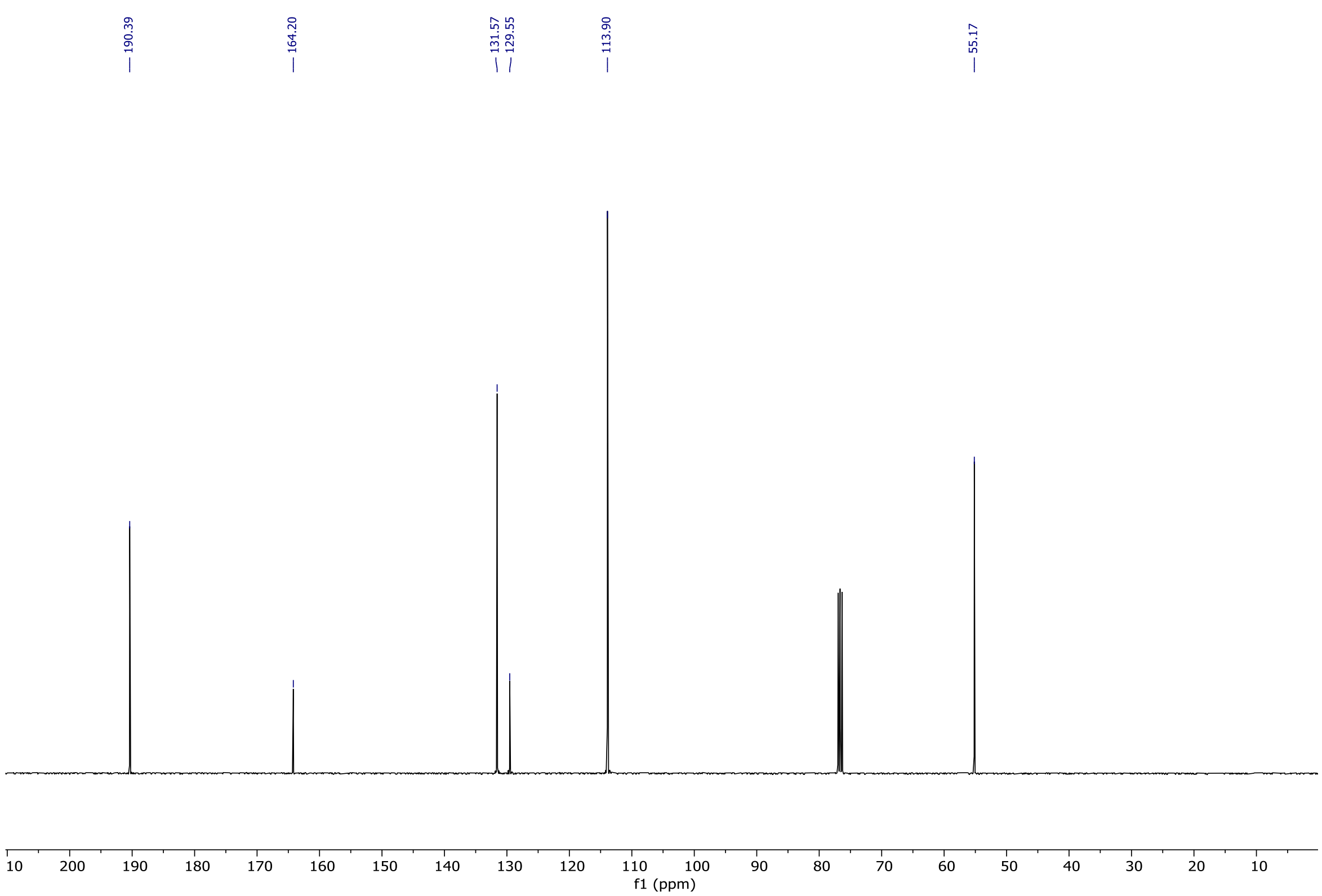

*4-(tert-Butyl)benzaldehyde (**2b**)*

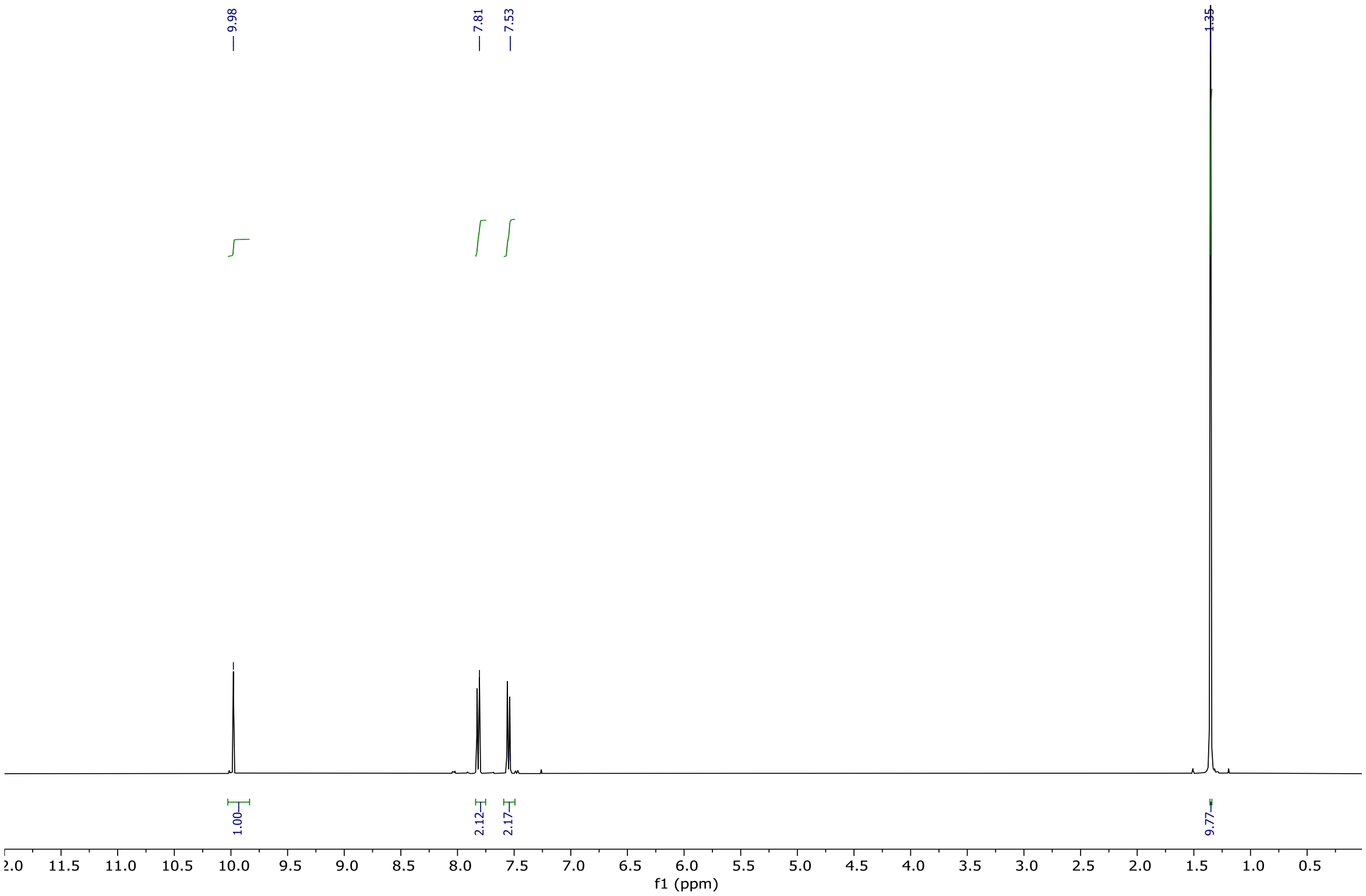

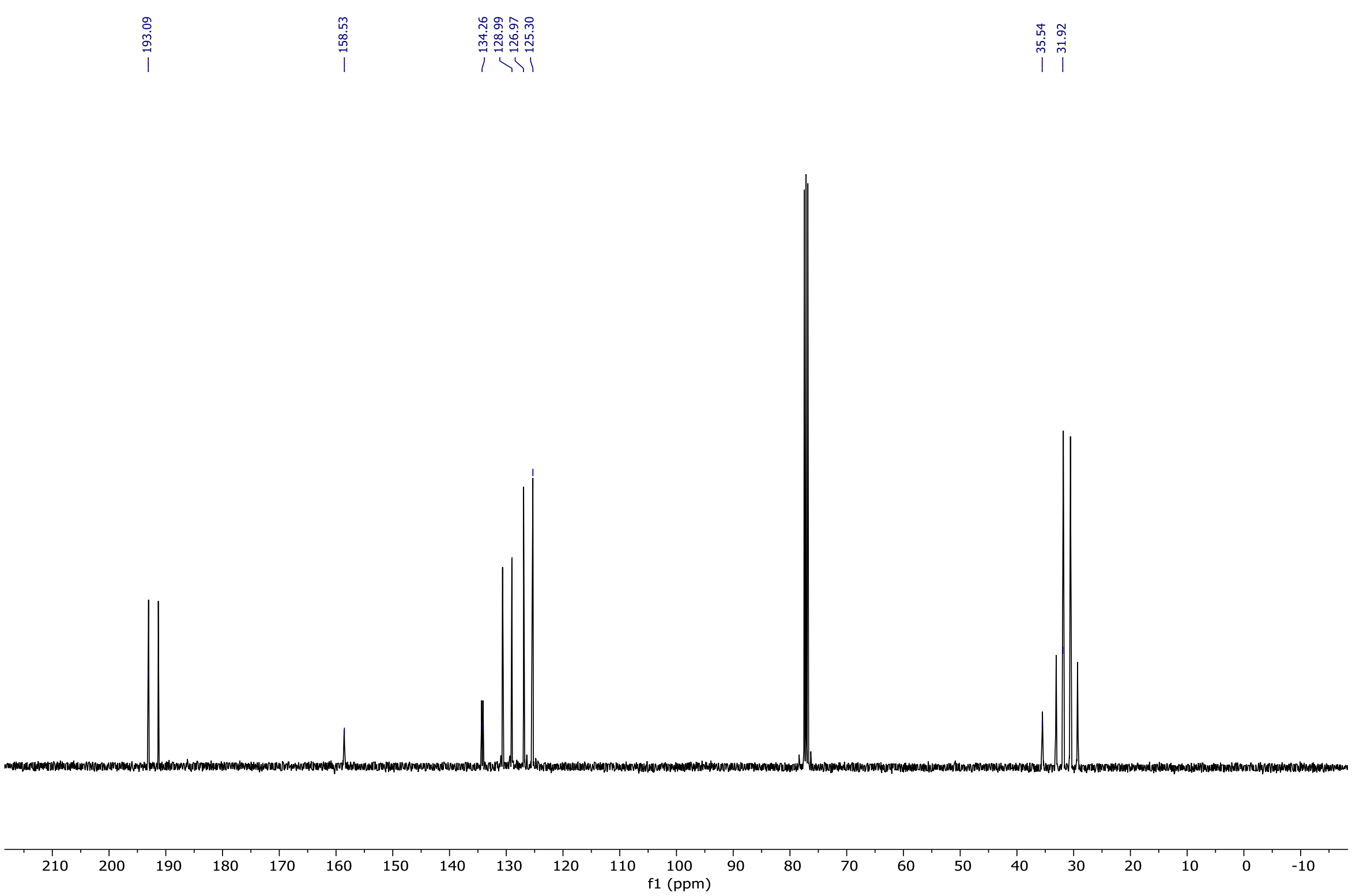

*2-Phenylacetaldehyde (**2c**)*

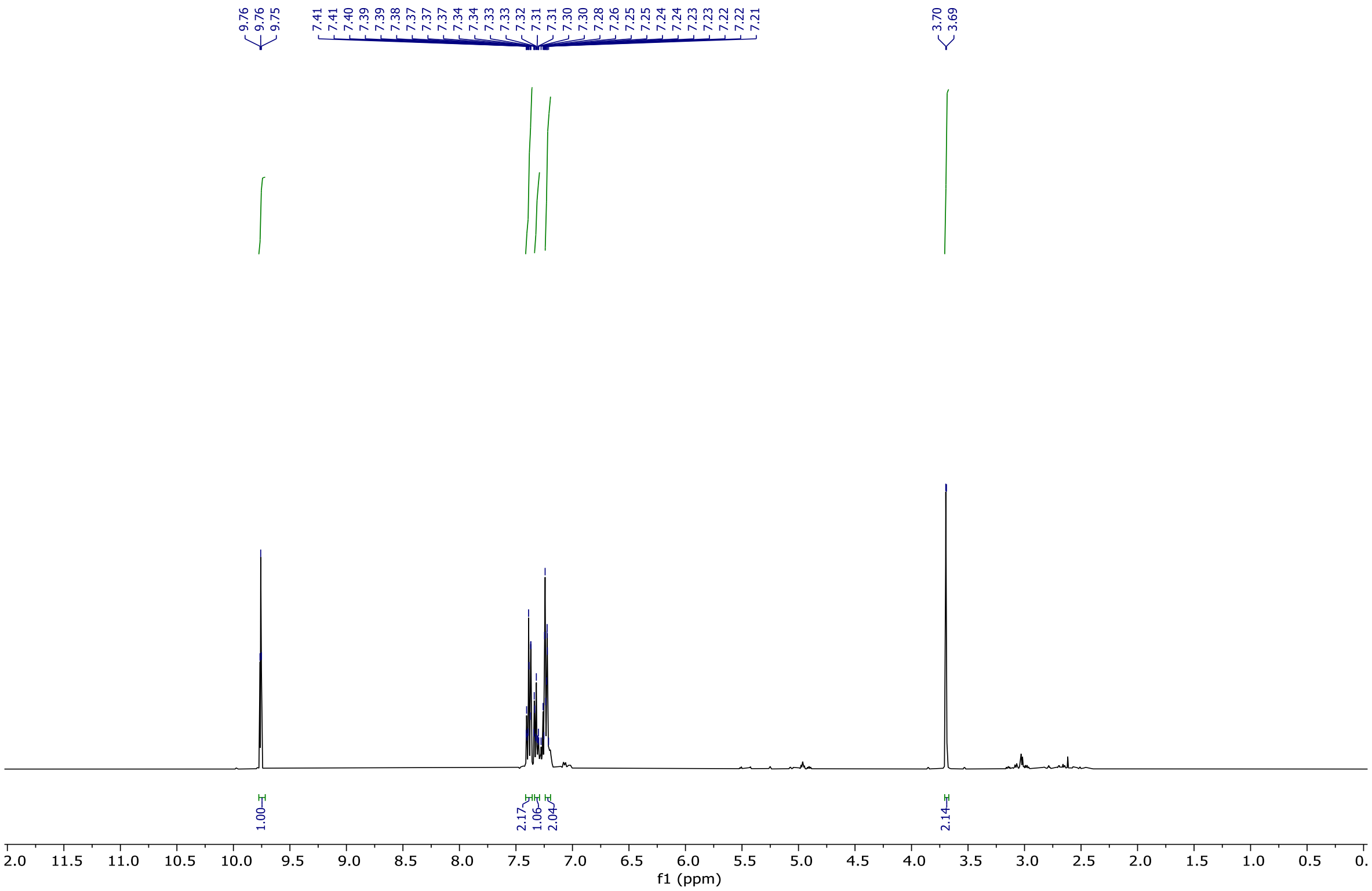

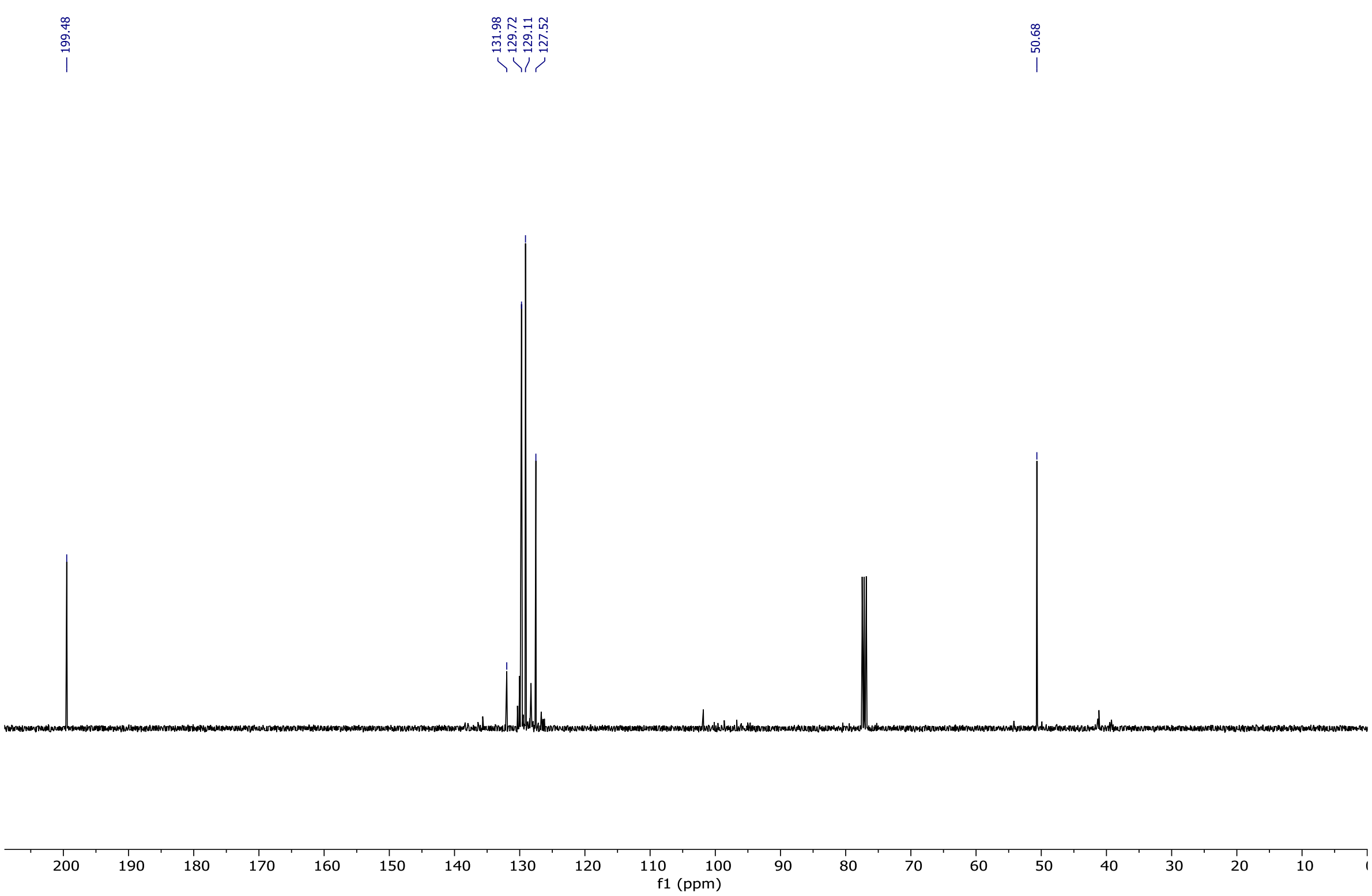

*4-Chlorobenzaldehyde (**2d**)*

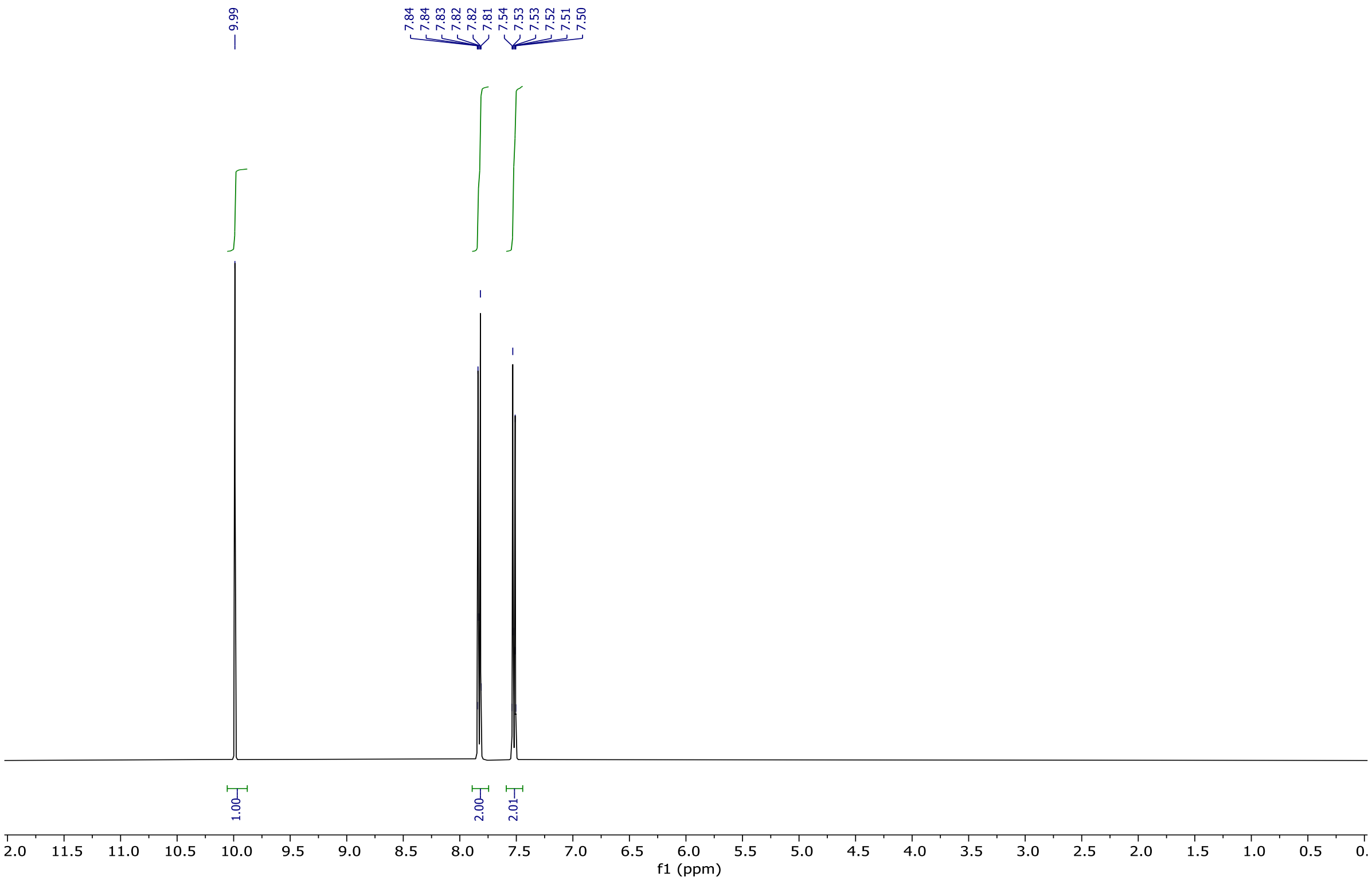

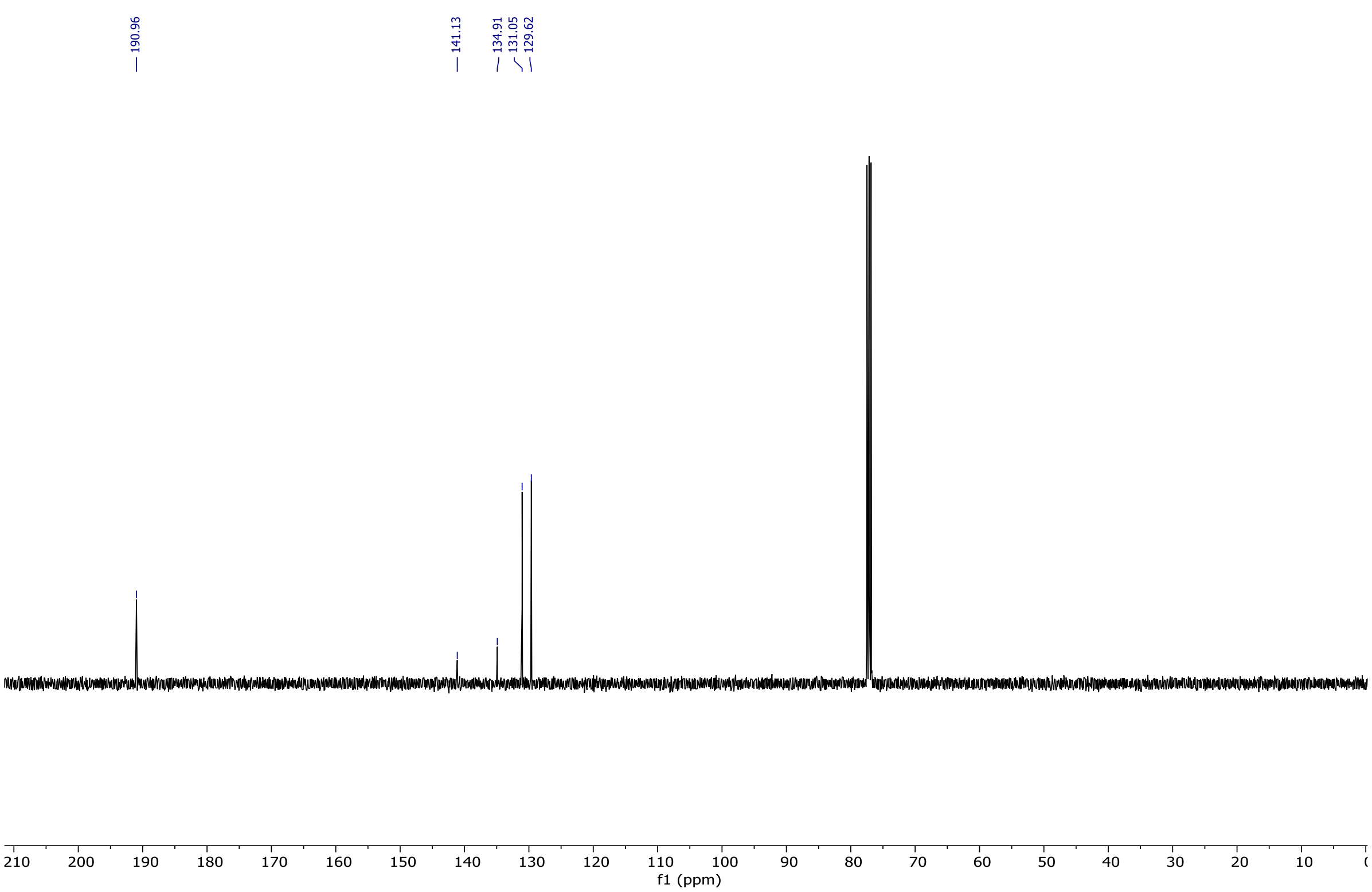

*4-Iodobenzaldehyde (**2e**)*

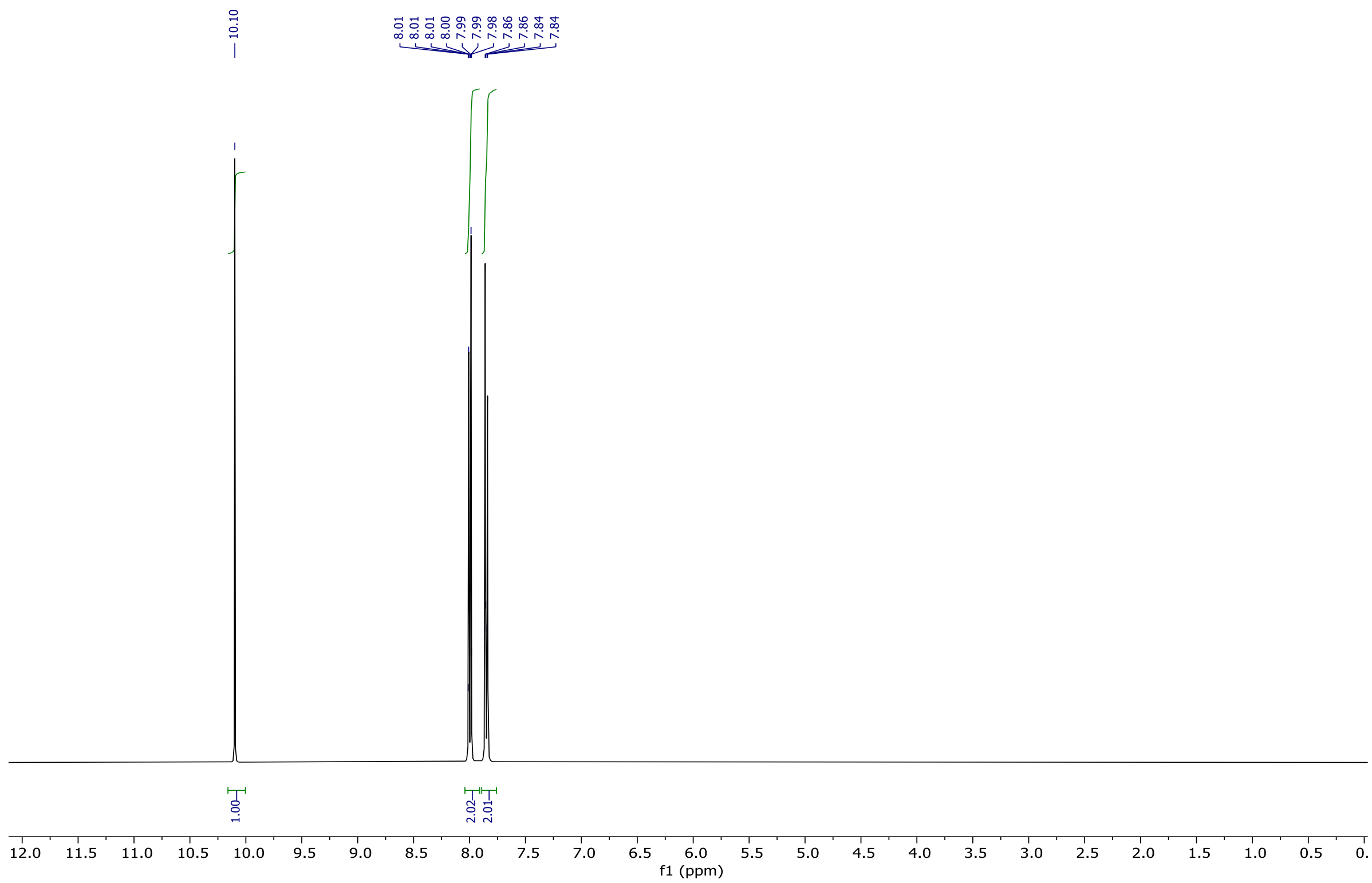

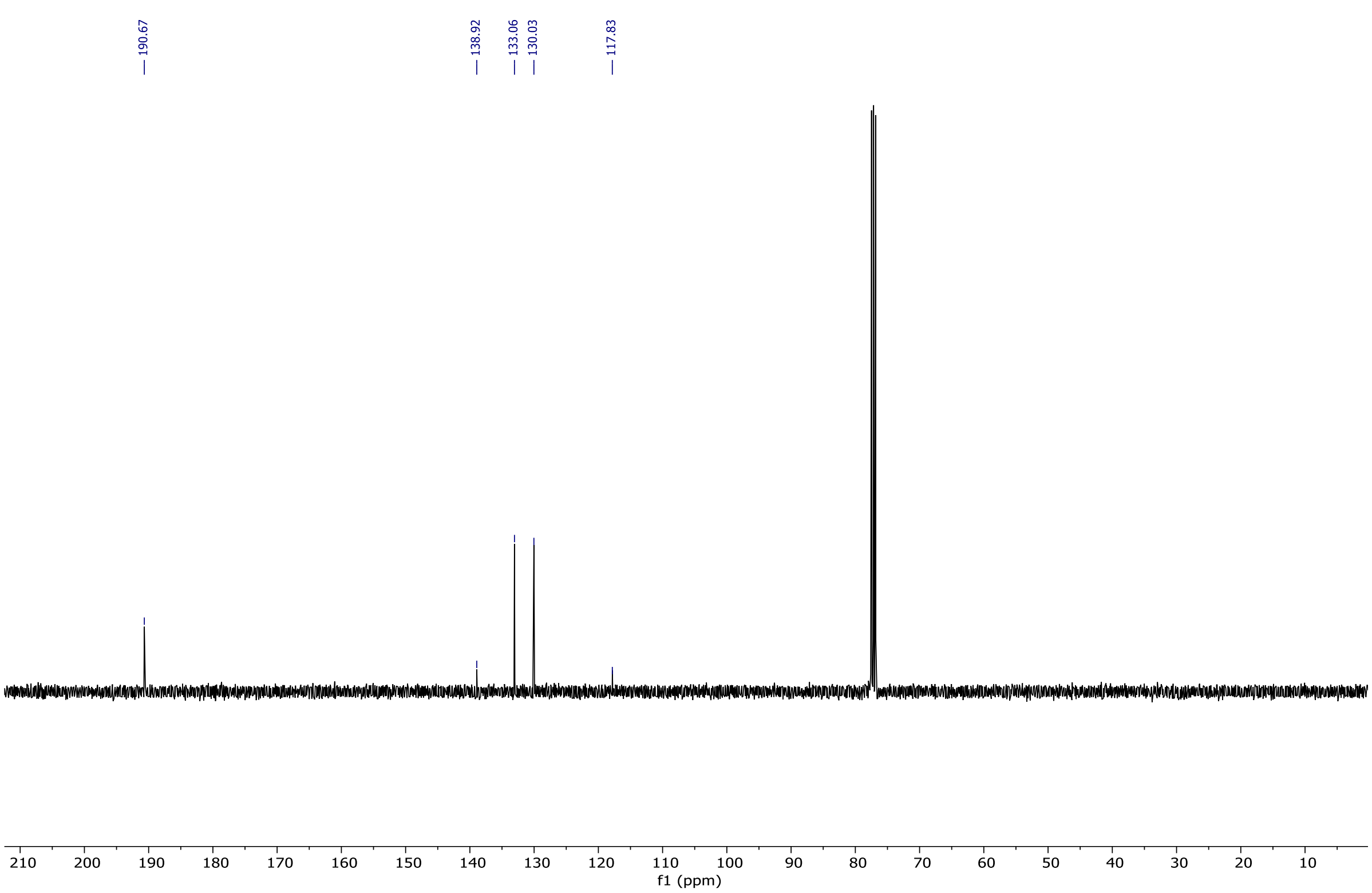

*4-Nitrobenzaldehyde (**2f**)*

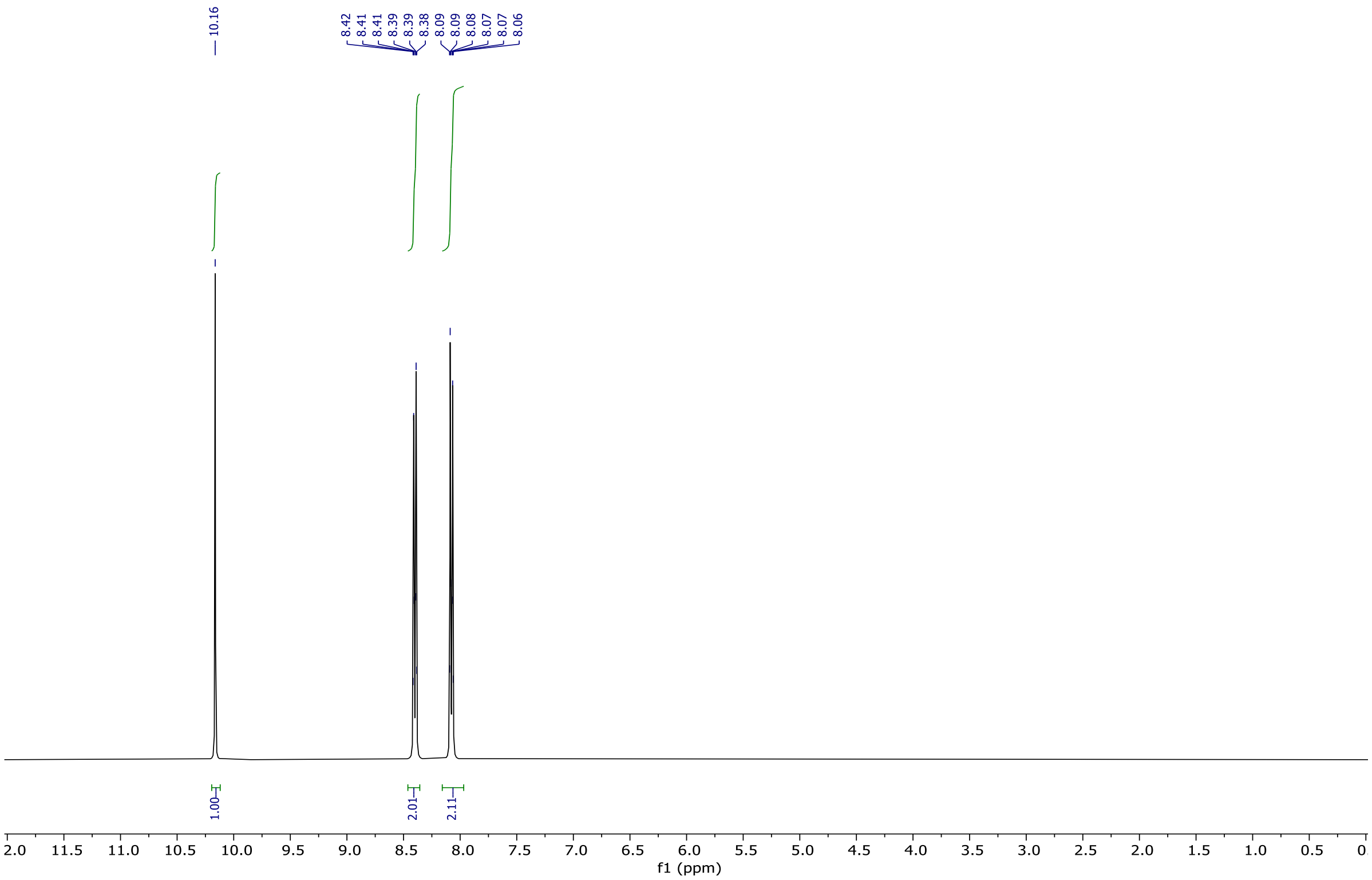

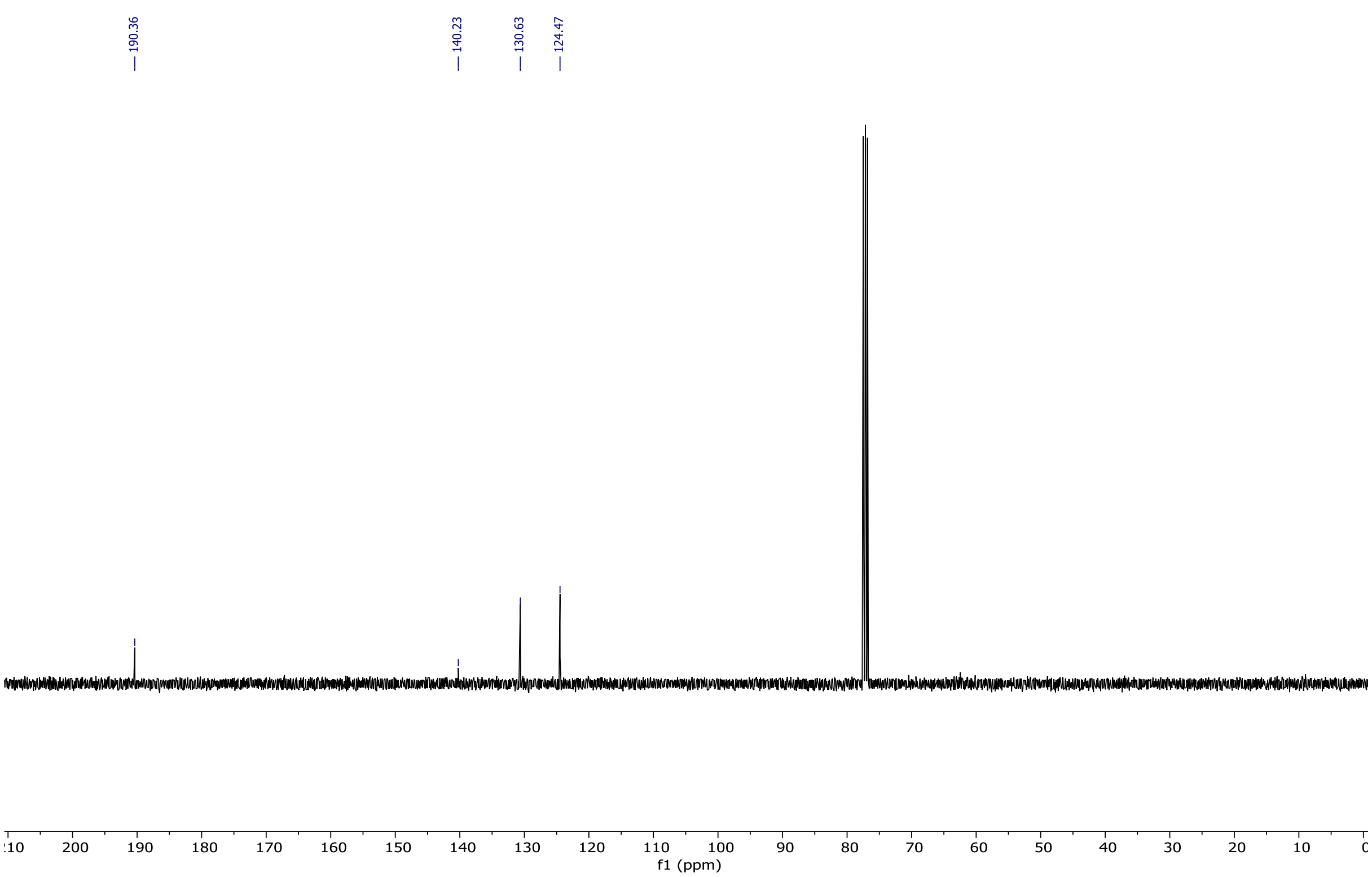

*4-Cyanobenzaldehyde (**2g**)*

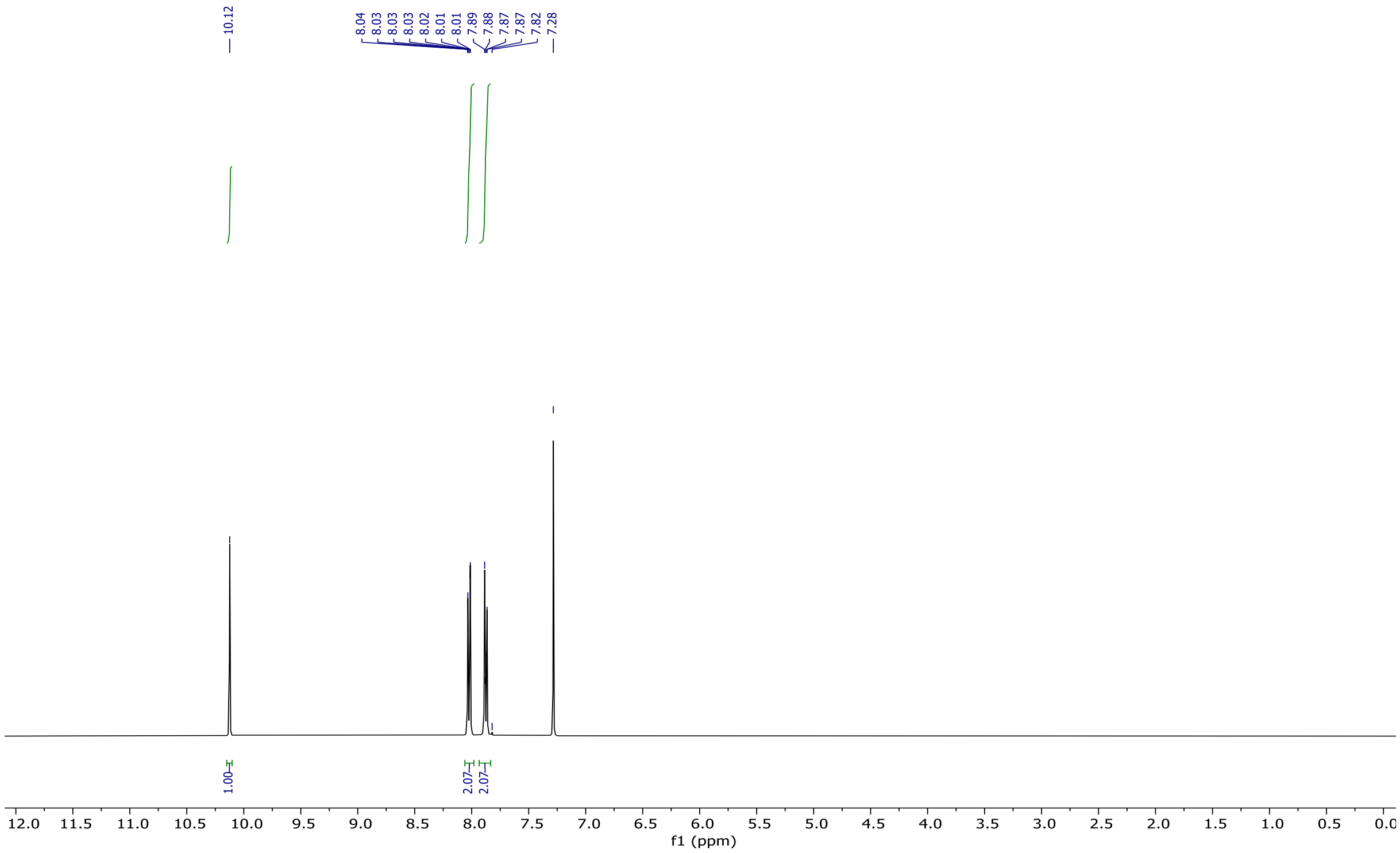

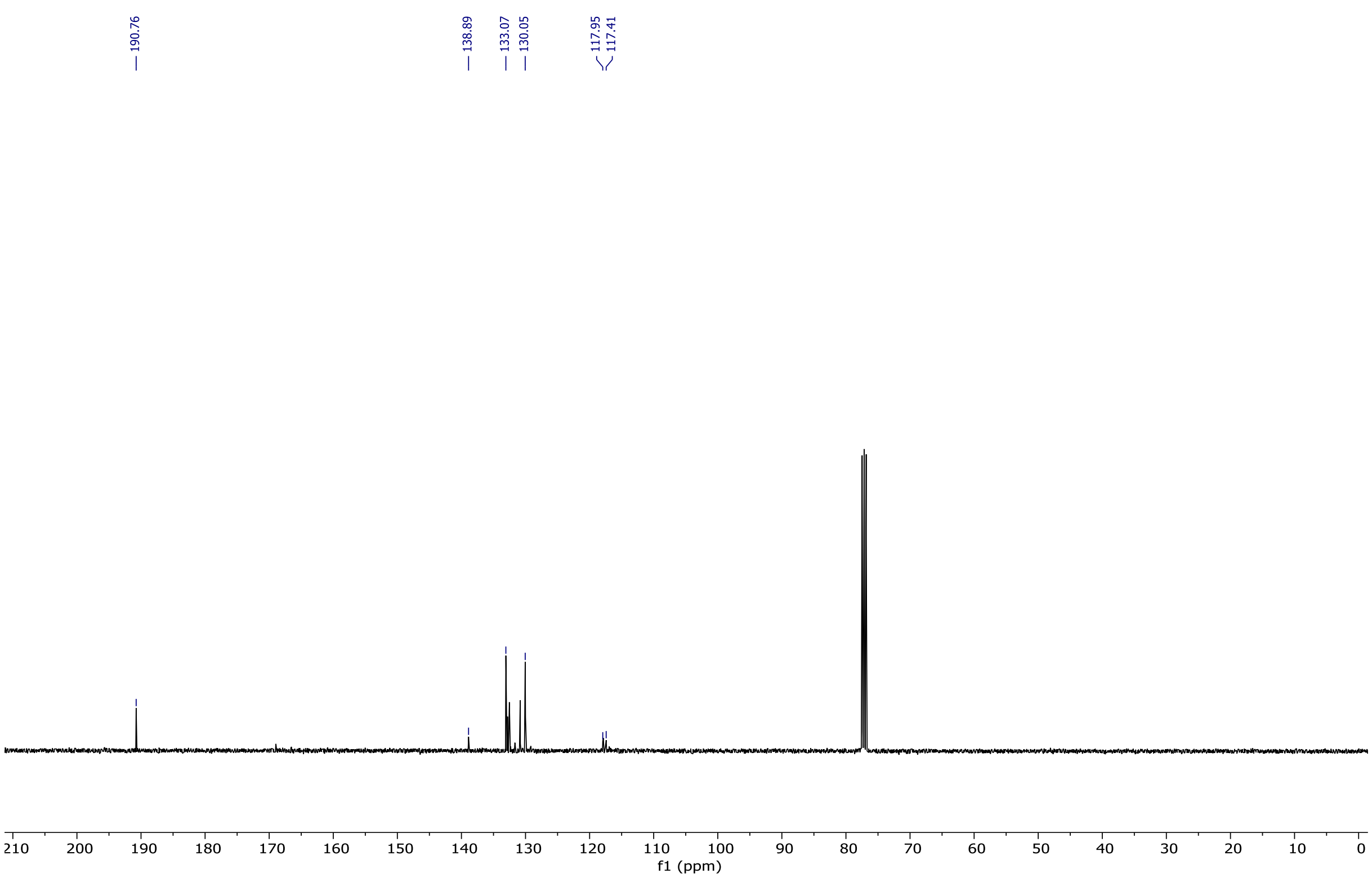

*Benzaldehyde (**2h**)*

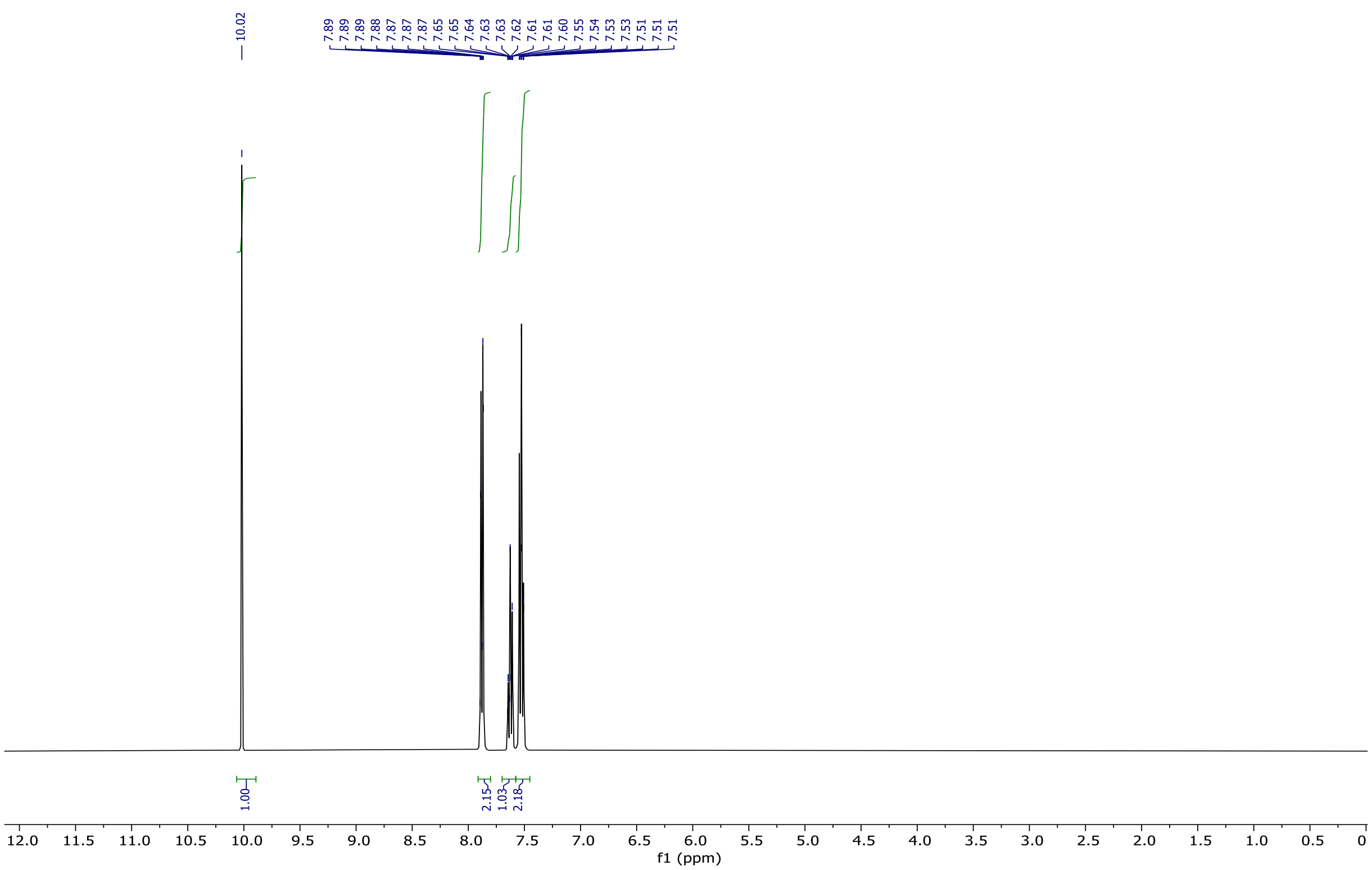

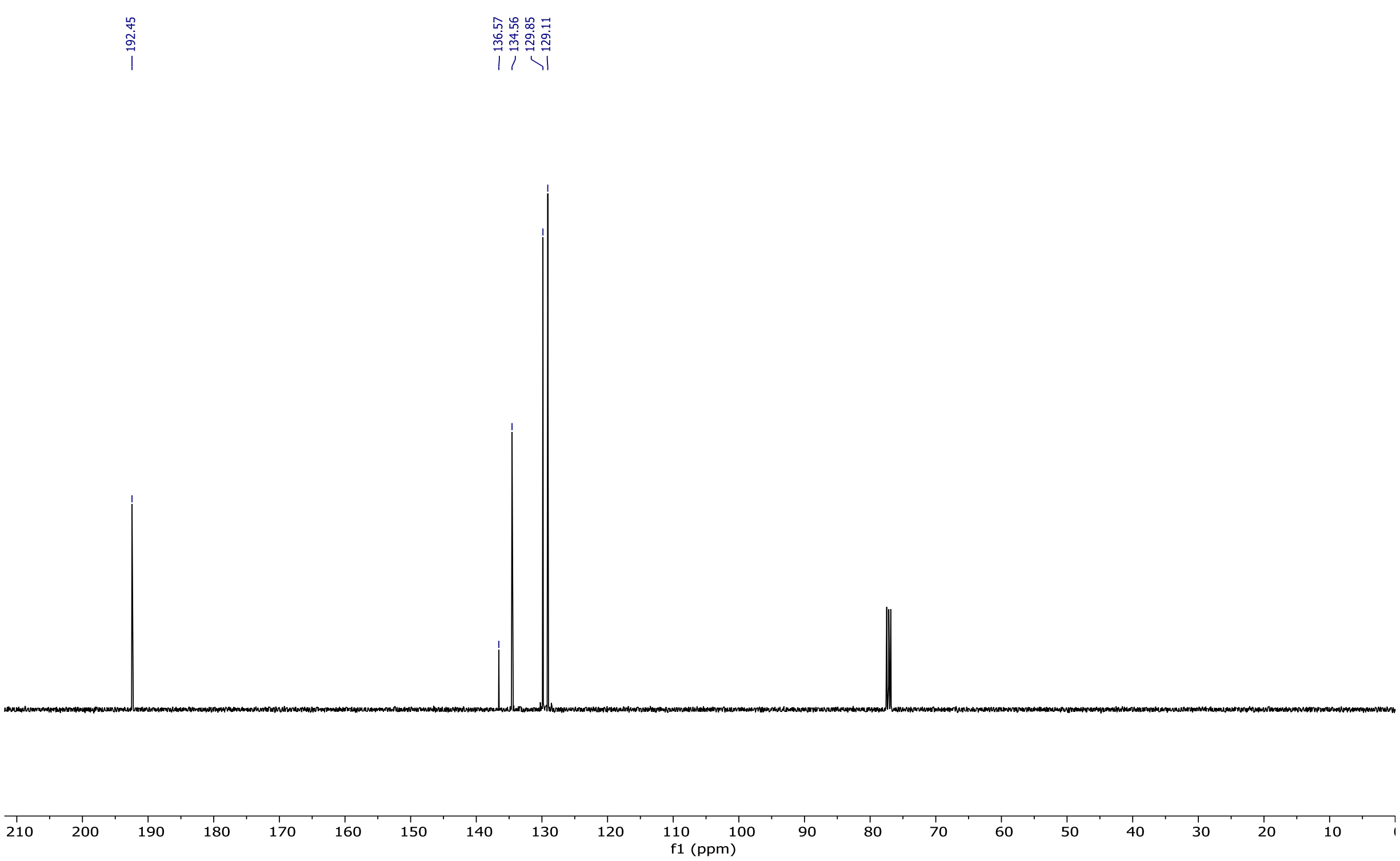

*Benzophenone (**2i**)*

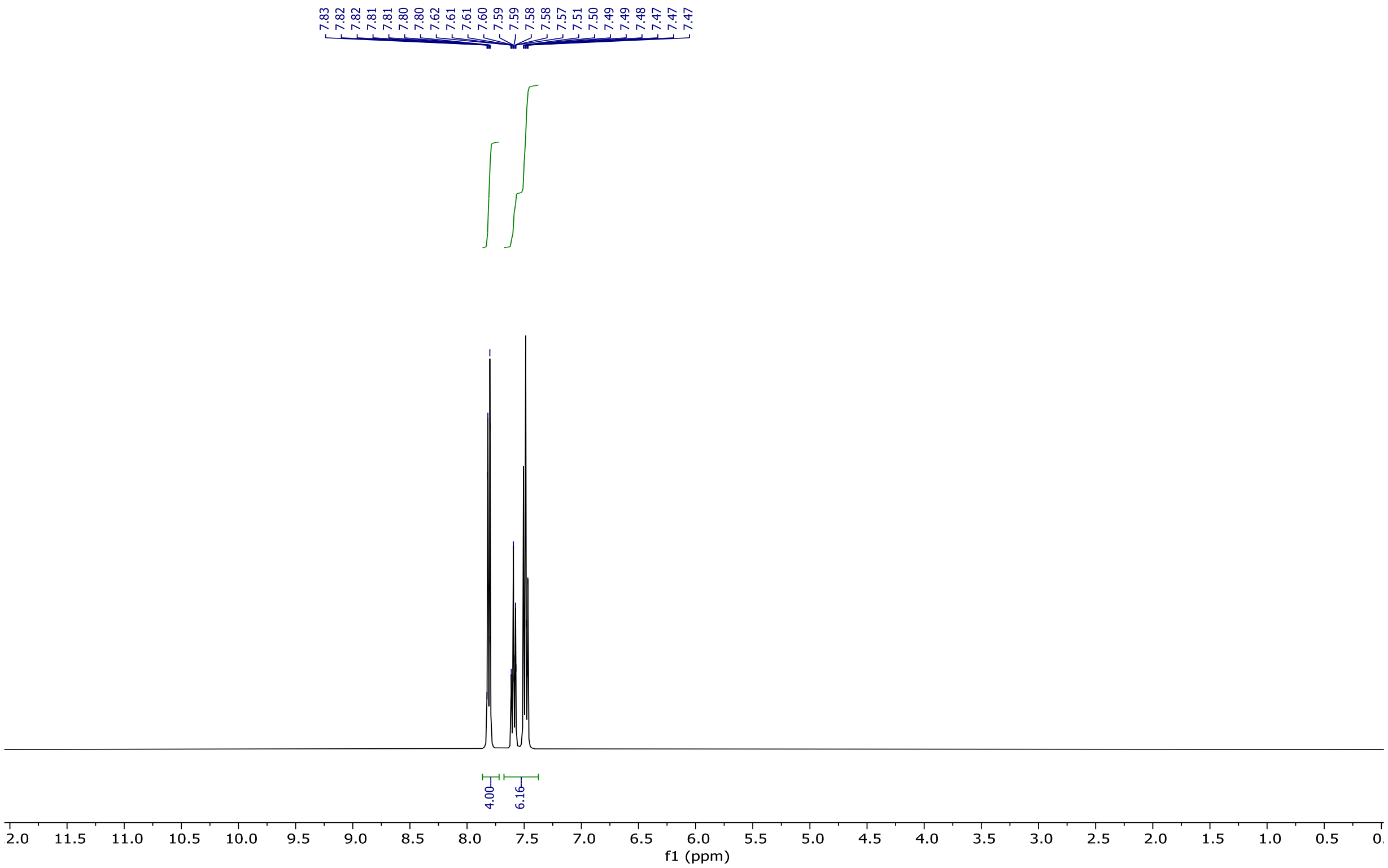

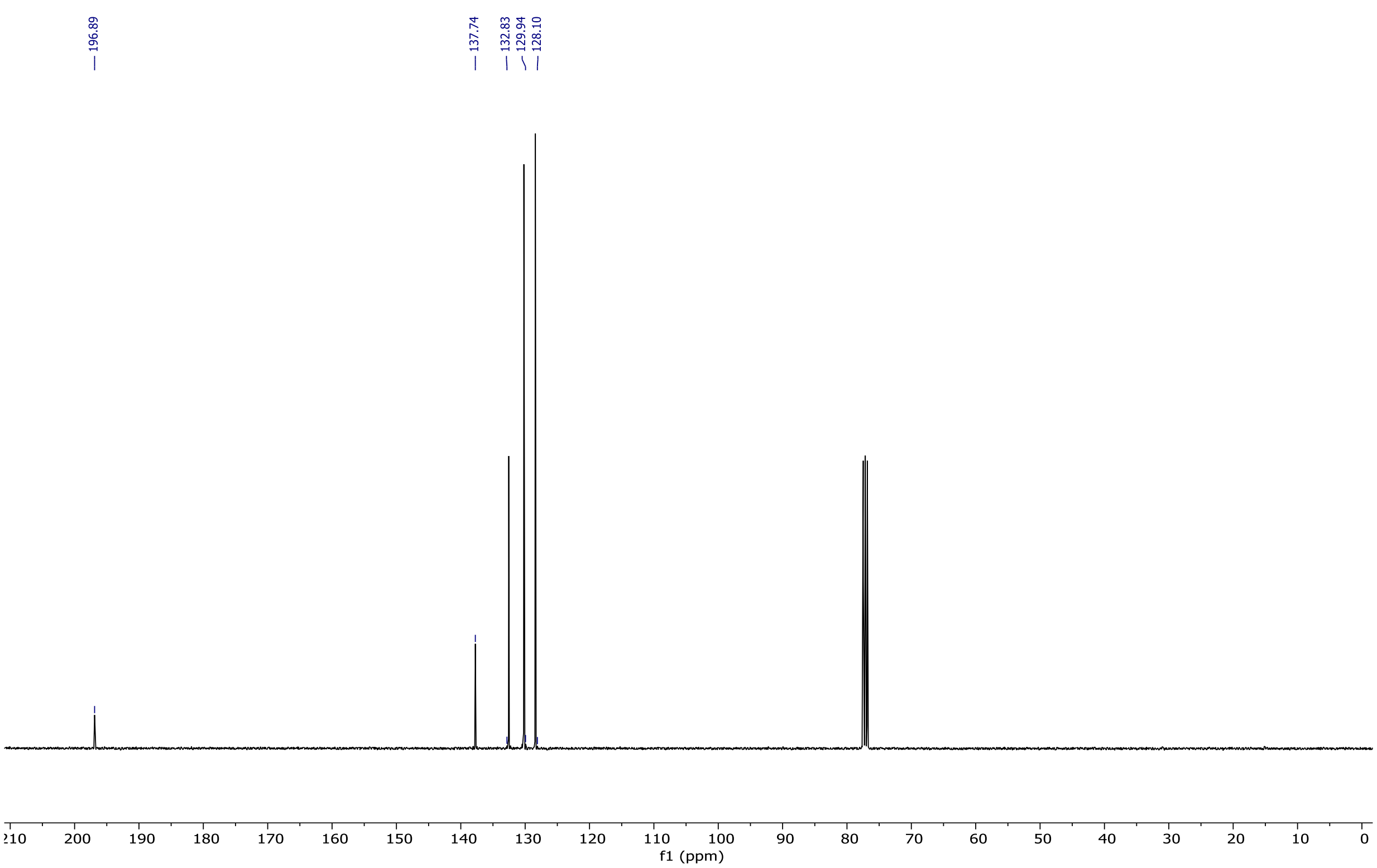

*9H-Fluoren-9-one (**2j**)*

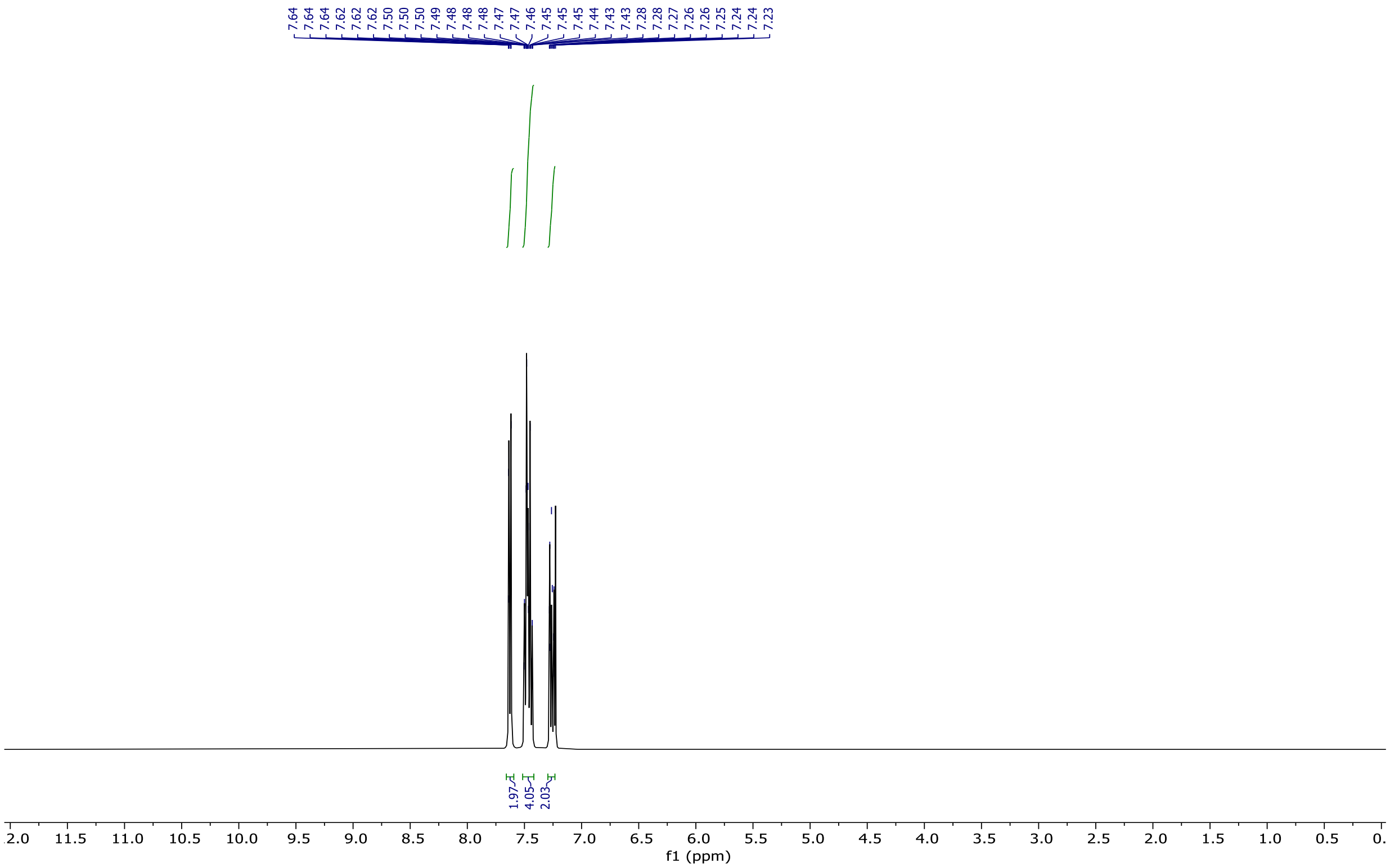

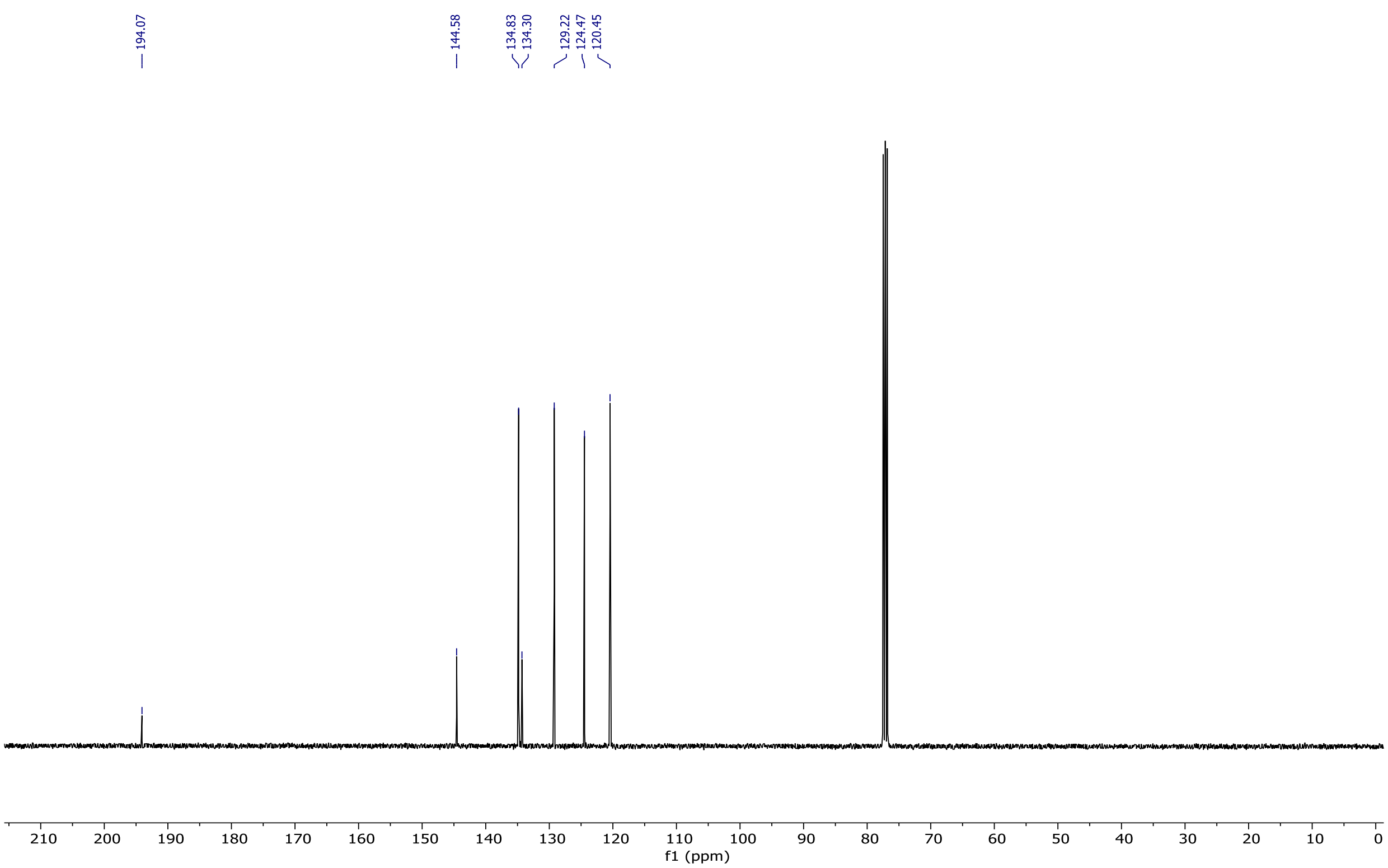

*3,4-Dihydronaphthalen-1(2H)-one (**2k**)*

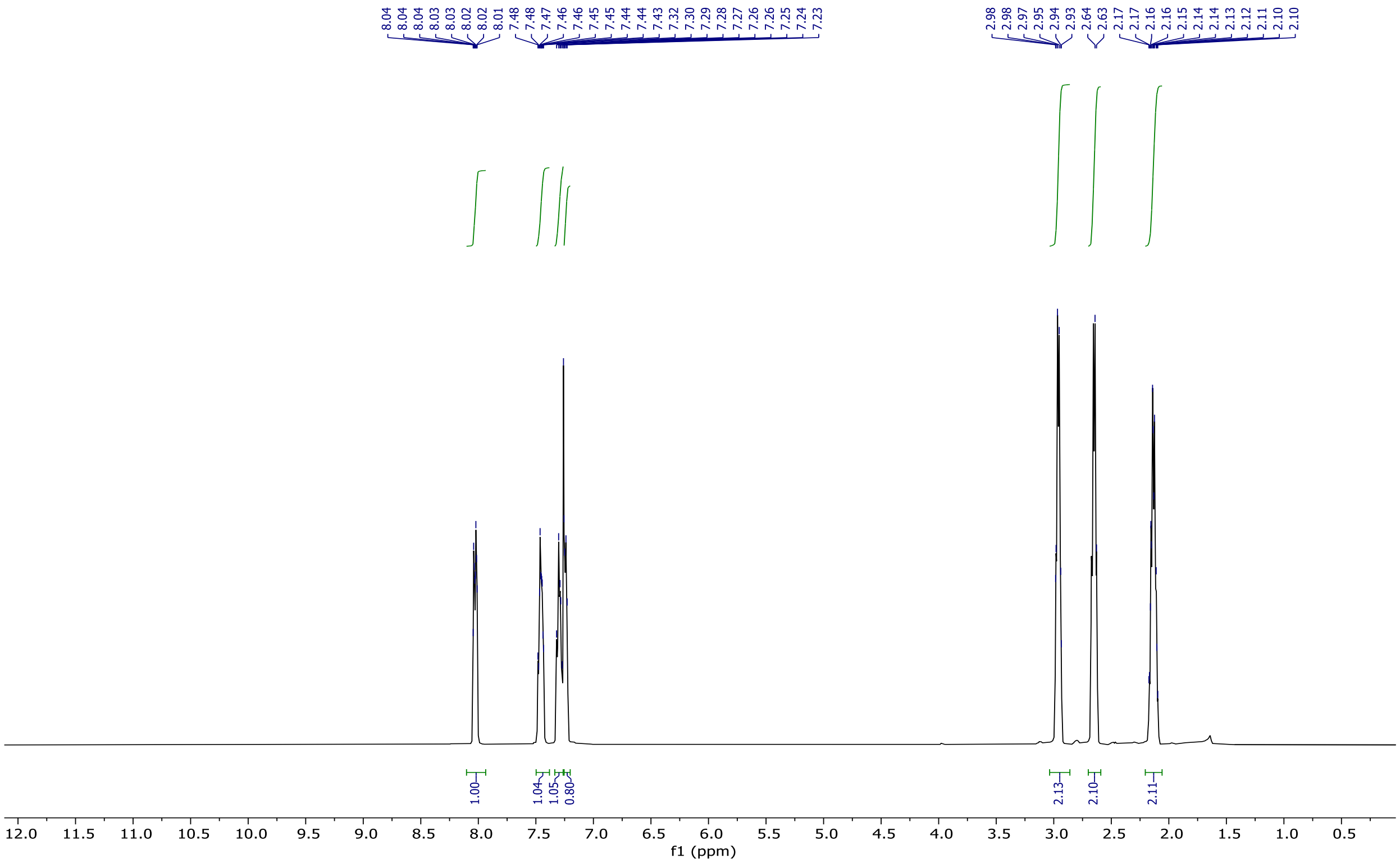

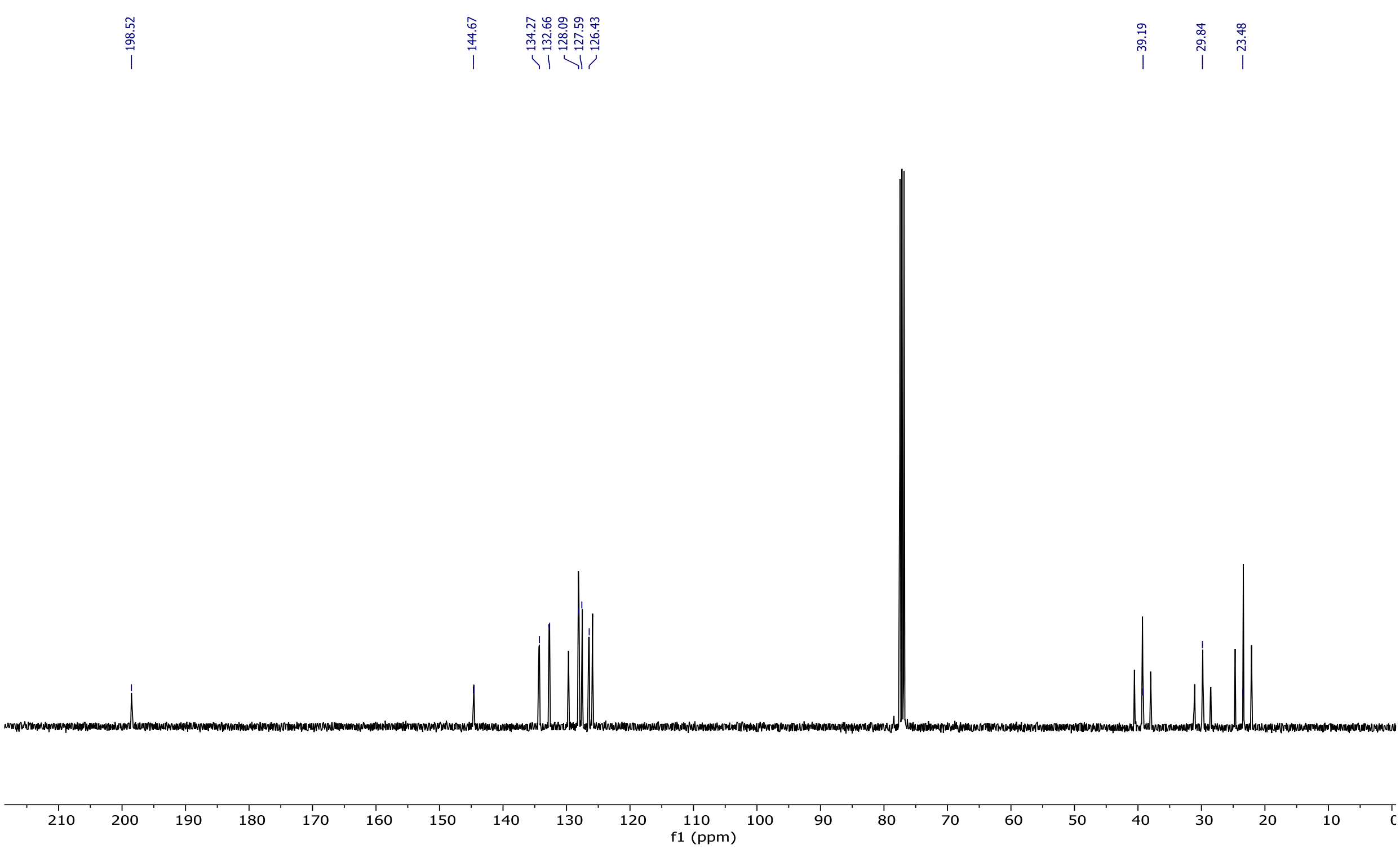

References

1. Waghmare, V. S.; Bhagwat, S. S. Engineering Aspects of Oxidation of 4-Methylanisole. *J. Indian Chem. Soc.* **2024**, *101*, 101121.
2. Zhang, L.; Li, R.-H.; Li, X.-X.; Wang, S.; Liu, J.; Hong, X.-X.; Dong, L.-Z.; Li, S.-L.; Lan, Y.-Q. Photocatalytic aerobic oxidation of $C(sp^3)$–H bonds. *Nat. Commun*. **2024**, *15*, 537.
3. Berniak, T.; Łątka, P.; Rokicińska, A.; Sidor, K.; Kuśtrowski, P. Selective Aerobic Oxidation of p-Methoxytoluene by Co(II)-Promoted NHPI Incorporated into a Cross-Linked Copolymer Structure. *Catalysts* **2021**, *11*, 1474.
4. Di Paola, A.; Bellardita, M.; Palmisano, L.; Barbieriková, Z.; Brezová, V. Influence of crystallinity and OH surface density on the photocatalytic activity of TiO_2 powders. *J. Photochem. Photobiol., A* **2014**, *273*, 59–67.
5. Qamar M.; Elsayed R. B.; Alhooshani K. R.; Ahmed, M. I.; Bahnemann, D. W. Highly Efficient and Selective Oxidation of Aromatic Alcohols Photocatalyzed by Nanoporous Hierarchical Pt/Bi_2WO_6 in Organic Solvent-Free Environment. *ACS Appl. Mater. Interfaces*, **2015**, *7*, 1257–1269.
6. Zhang, T.; Wen, Y.; Xia, C.; Xu, X.; Wang, G.; Guo, J.; Zhang, Y. Enhanced Oxidation of p-Methoxytoluene to p-Methoxybenzaldehyde Using Na-Doped Co-Mn-Al Oxide Catalysts. *Appl. Catal. A: Gen*. **2025**, *705*, 120465.
7. Wongthep, S.; Pluengphon, P.; Tantraviwat, D.; Panchan, W.; Boochakiat, S.; Jarusuphakornkul, K.; Wu, Q.; Chen, J.; Inceesungvorn, B. New Visible-Light-Driven $Bi_2MoO_6/Cs_3Sb_2Br_9$ Heterostructure for Selective Photocatalytic Oxidation of Toluene to Benzaldehyde. *J. Colloid Interface Sci*. **2024**, *655*, 32–42.
8. Reddy, B. M.; Kumar, M. V.; Ratnam, K. J. Preparation and characterization of V_2O_5/MgO catalysts for selective oxidation of 4-methylanisole to anisaldehyde. *Res. Chem. Intermed*. **1998**, *24*, 919–931.
9. Fernandes, R. A.; Sampaio, M. J.; Faria, J. L.; Silva, C. G. Aqueous Solution Photocatalytic Synthesis of p-Anisaldehyde by Using Graphite-Like Carbon Nitride Photocatalysts Obtained via the Hard-Templating Route. *RSC Adv*. **2020**, *10*, 19431–19442.

10. Zhang, J.; Du, J.; Zhang, C.; Liu, K.; Yu, F.; Yuan, Y.; Duan, B.; Liu, R. Selective Oxidation of Alkylarenes to the Aromatic Ketones or Benzaldehydes with Water. *Org. Lett.* **2022**, *24*, 1152–1157.

11. Iqbal, N; Cho, E. J. Formation of Carbonyl Compounds from Amines through Oxidative C–N Bond Cleavage using Visible Light Photocatalysis and Applications to *N*-PMB-Amide Deprotection. *Adv. Synth. Catal.* **2015**, *357*, 2187–2192.

12. Li, H.; Misal Castro, L. C.; Zheng, J.; Roisnel, T.; Dorcet, V.; Sortais, J.-B.; Darcel, C. Selective Reduction of Esters to Aldehydes under the Catalysis of Well-Defined NHC–Iron Complexes. *Angew. Chem. Int. Ed.* **2013**, *52*, 8045– 8049.

13. Tinnis, F.; Volkov, A.; Slagbrand, T.; Adolfsson, H. Chemoselective Reduction of Tertiary Amides under Thermal Control: Formation of either Aldehydes or Amines. *Angew. Chem. Int. Ed.* **2016**, *55*, 4562– 4566.

14. Mittal, R.; Awasthi, S. K. Metal–Organic Framework-Derived $Mn_3O_4/Co_3O_4/C/SiO_2$ Nanostructures for Catalytic Oxidation Reactions. *ACS Appl. Nano Mater*. **2022**, *5*, 7831– 7840.

15. Stache, E. E.; Ertel, A. B.; Rovis, T.; Doyle, A. G. Generation of Phosphoranyl Radicals via Photoredox Catalysis Enables Voltage–Independent Activation of Strong C–O Bonds. *ACS Catal*. **2018**, *8*, 11134– 11139.

16. Niu, K.; Shi, X.; Ding, L.; Liu, Y.; Song, H.; Wang, Q. HCl-Catalyzed Aerobic Oxidation of Alkylarenes to Carbonyls. *ChemSusChem* **2022**, *15*, e202102326.
